# Supplementary material for: Synthesis, characterization and reactivity of a series of alkaline earth and rare earth iminophosphoranomethanide complexes
Source: Commun Chem. 2025 Jun 5;8:175. doi: 10.1038/s42004-025-01572-5 (PMC12141727; doi:10.1038/s42004-025-01572-5)
Supplement: Supplementary file 2 — Supplementary information file [file 42004_2025_1572_MOESM2_ESM.pdf]

Synthesis, characterization and reactivity of a series of alkaline earth and rare earth iminophosphoranomethanide complexes

Matthew P. Stevens,[a†] Yu Liu,[a†] Elias Alexopoulos,[a] Shifaa S. A. Xec Daudo,[a] Rebecca R. Hawker,[a] Adam Khan,[a] Luis Lezama,[b] Daniel Reta[c,d,e]* and Fabrizio Ortu[a]*

[a]School of Chemistry, University of Leicester, University Road, LE1 7RH, Leicester, UK

[b]Departamento de Química Orgánica e Inorgánica, Facultad de Ciencia y Tecnología, Universidad del País Vasco, Bº Sarriena s/n, 48940 Leioa, Spain

[c]Faculty of Chemistry, The University of the Basque Country UPV/EHU, Donostia, 20018 Spain

[d]Donostia International Physics Center (DIPC), Donostia, Euskadi 20018, Spain

[e]IKERBASQUE, Basque Foundation for Science, Bilbao, Euskadi 48011, Spain

*email: fabrizio.ortu@leicester.ac.uk, daniel.reta@ehu.eus

[†]These authors contributed equally.

Table of Contents

S1. Supplementary NMR data S2
S2. IR data S46
S3. UV-vis data S51
S4. Crystallography S52
S5. Magnetic characterization S59
S6. Theoretical calculations S60
S7. Supplementary References S71

S1. Supplementary NMR data

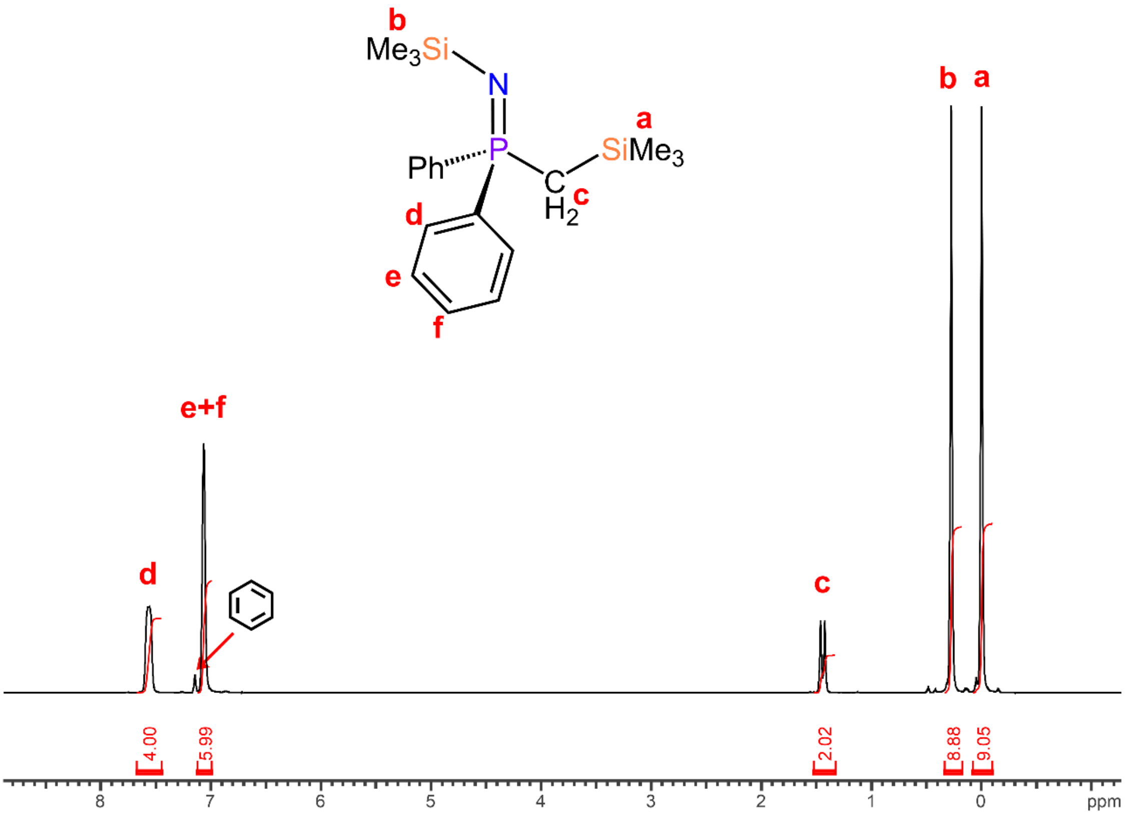

Figure S1: ^{1}H NMR (400 MHz, 298 K, C_6D_6) spectrum of **^{TMS}NPC-H$_2$**, with assignment.

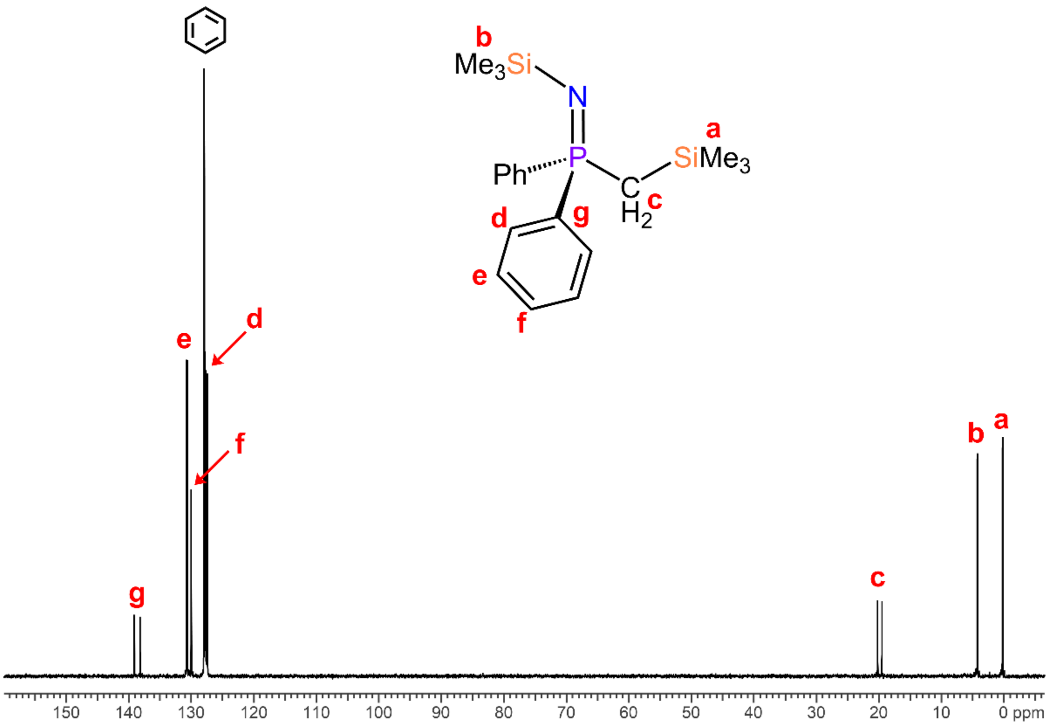

Figure S2: ^{13}C{^{1}H} NMR (101 MHz, 298 K, C_6D_6) spectrum of **^{TMS}NPC-H$_2$**, with assignment.

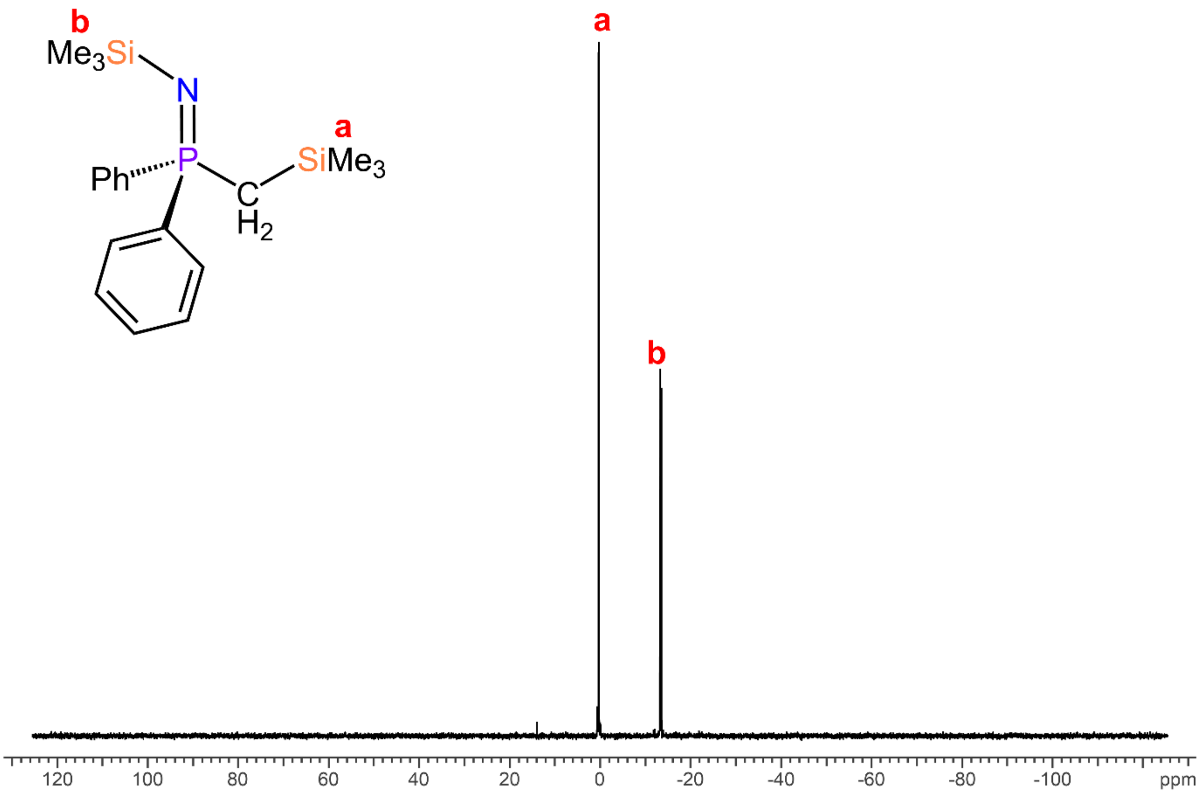

Figure S3: ^{29}Si{^{1}H} NMR (80 MHz, 298 K, C_6D_6) spectrum of **^{TMS}NPC-H$_2$**, with assignment.

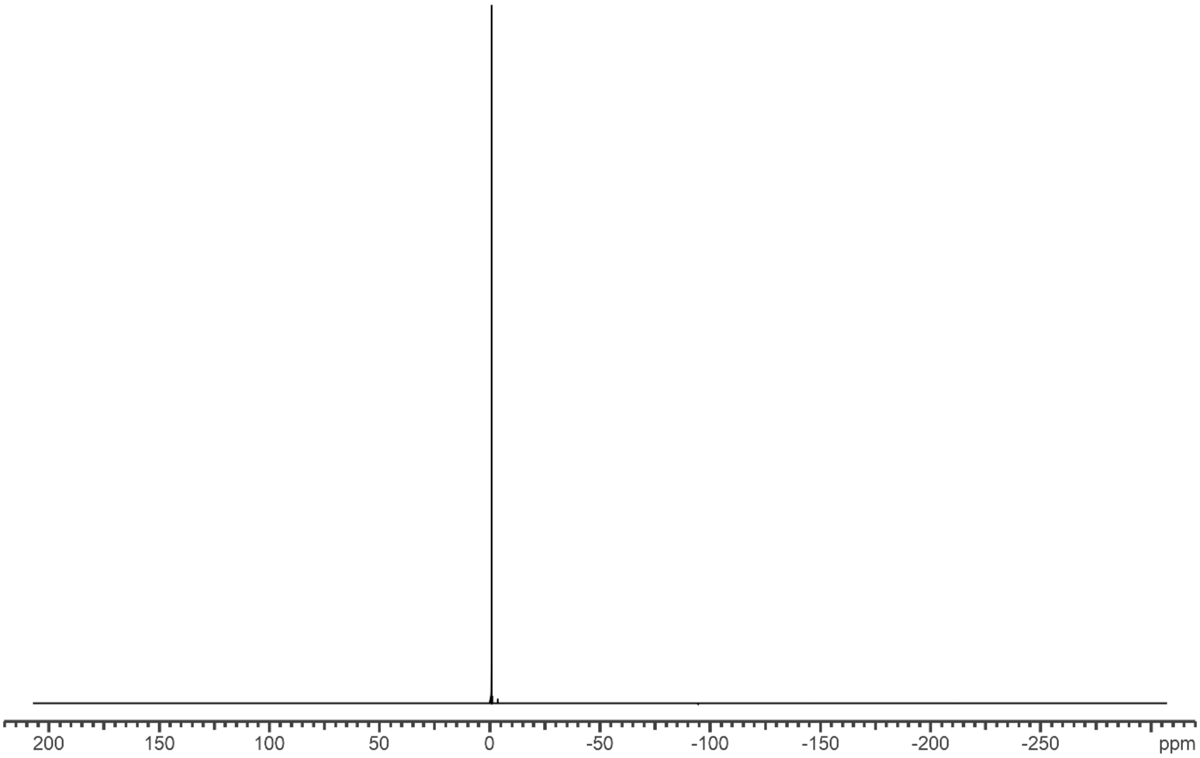

Figure S4: ^{31}P{^{1}H} NMR (162 MHz, 298 K, C_6D_6) spectrum of **^{TMS}NPC-H$_2$**, with assignment.

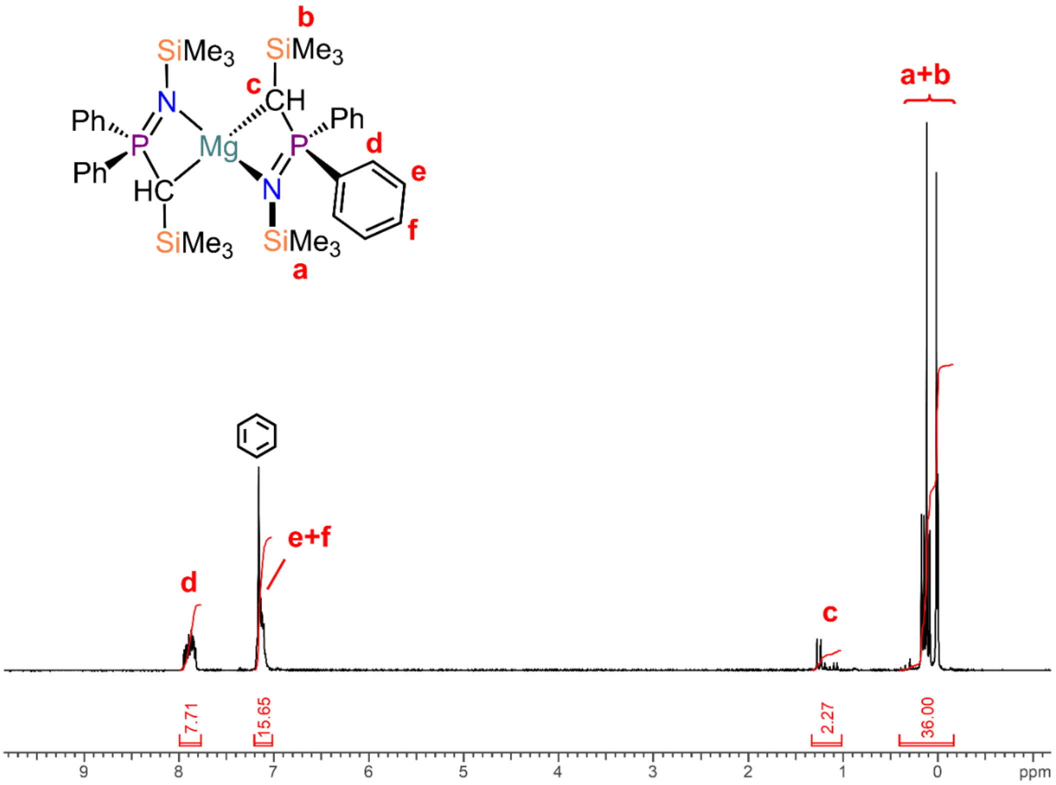

Figure S5: ^{1}H NMR (400 MHz, 298 K, C_6D_6) spectrum of **1-Mg**, with assignment.

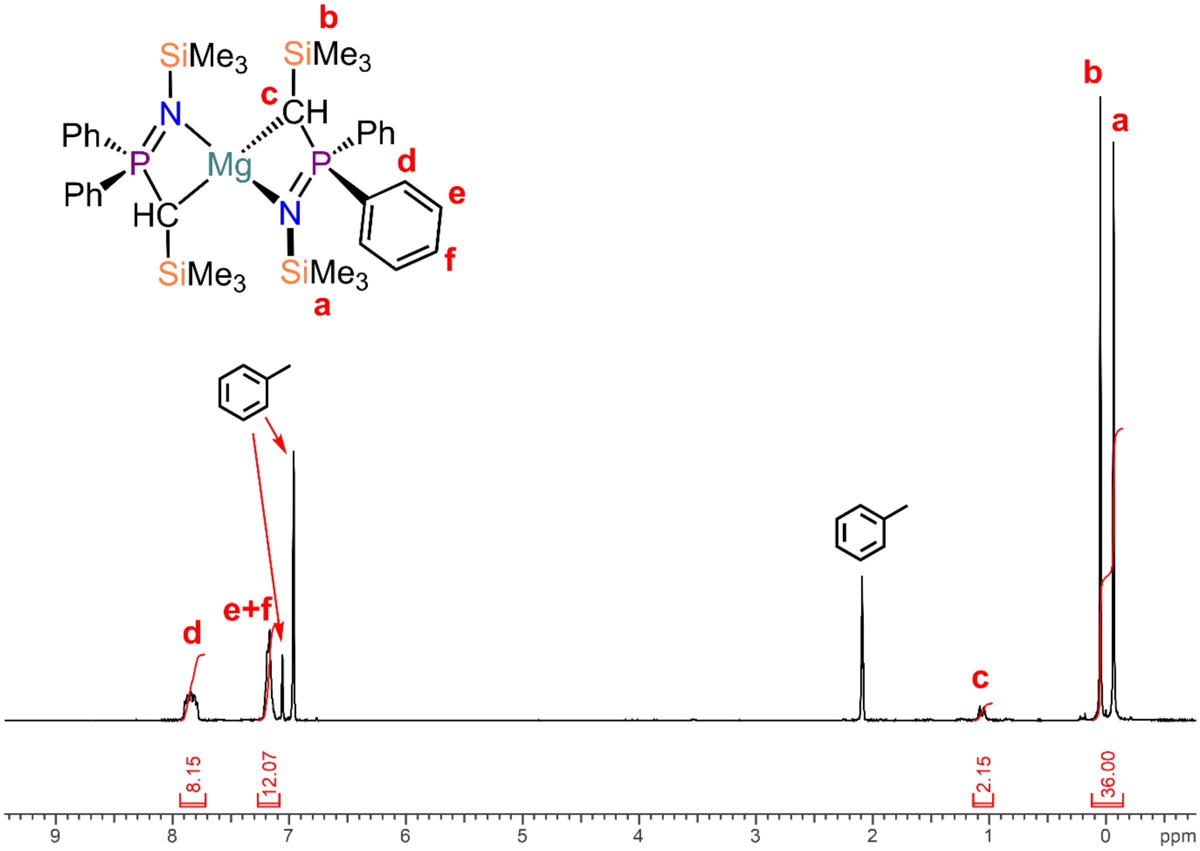

Figure S6: ^{1}H NMR (400 MHz, 378 K, C_7D_8) spectrum of **1-Mg**.

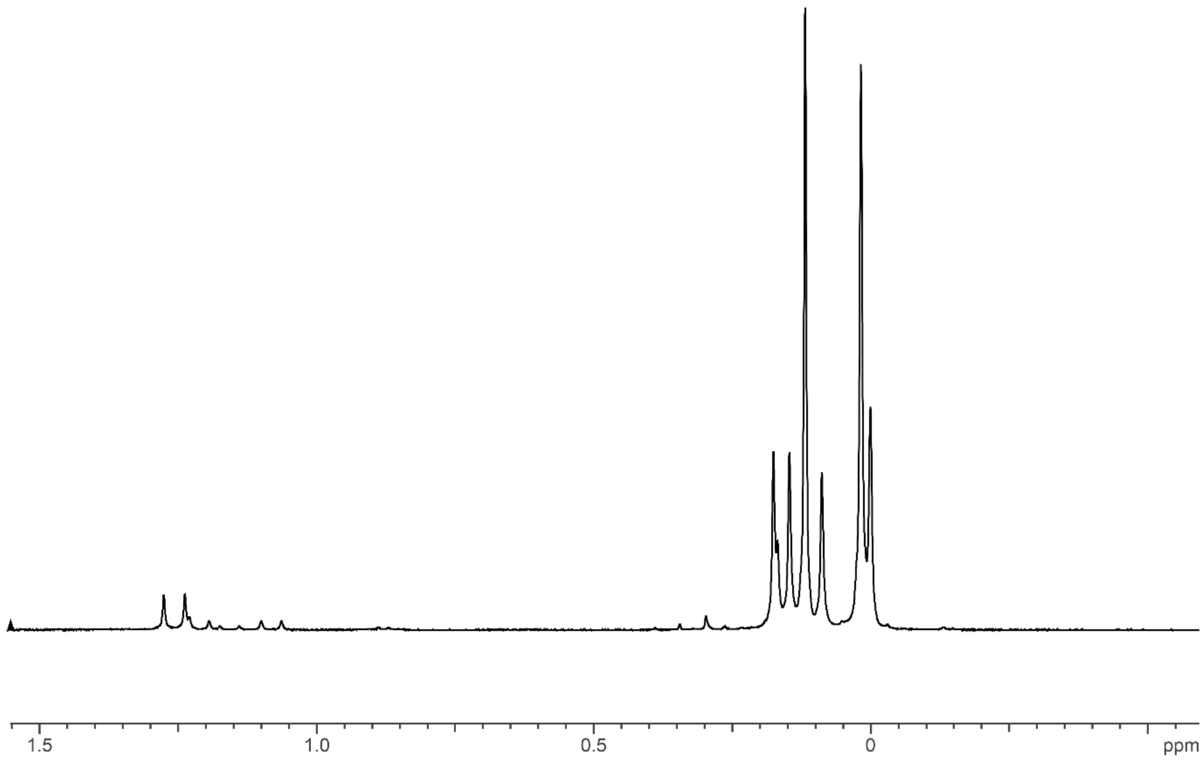

Figure S7: ^{1}H NMR (400 MHz, 298 K, C_6D_6) spectrum of **1-Mg** in the region –0.5 – 1.5 ppm.

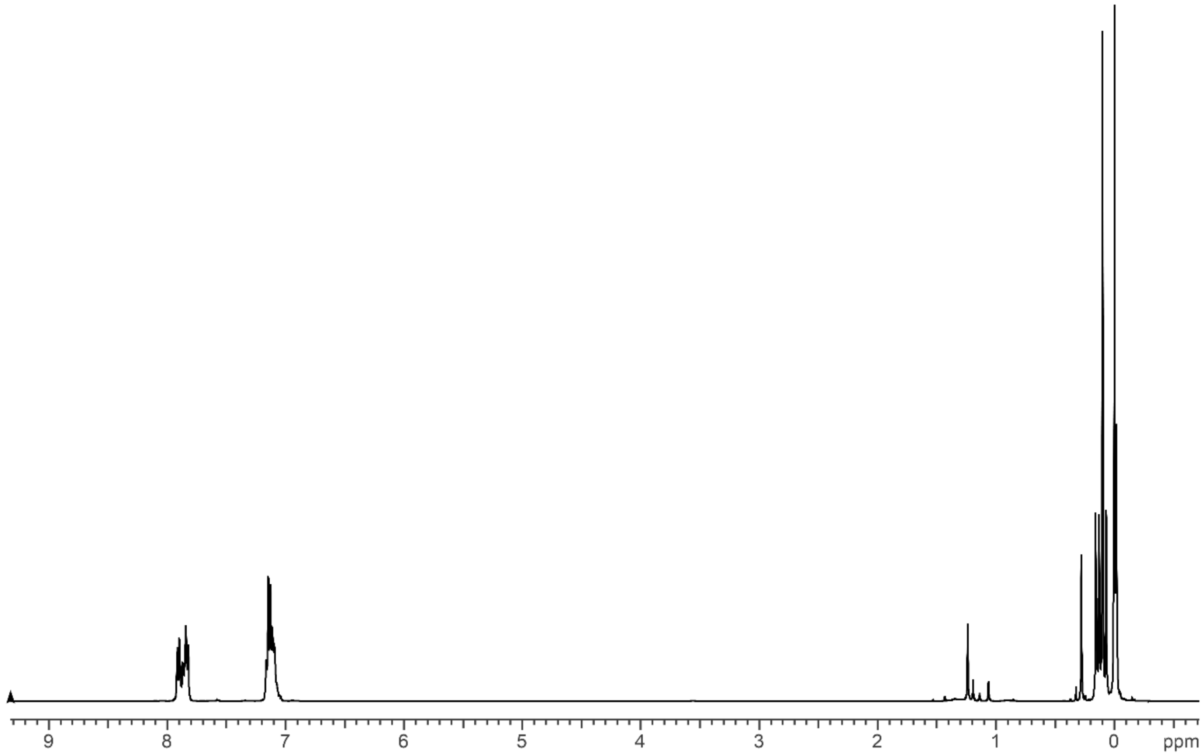

Figure S8: $^{1}H\{^{31}P\}$ NMR (400 MHz, 298 K, C_6D_6) spectrum of **1-Mg.**

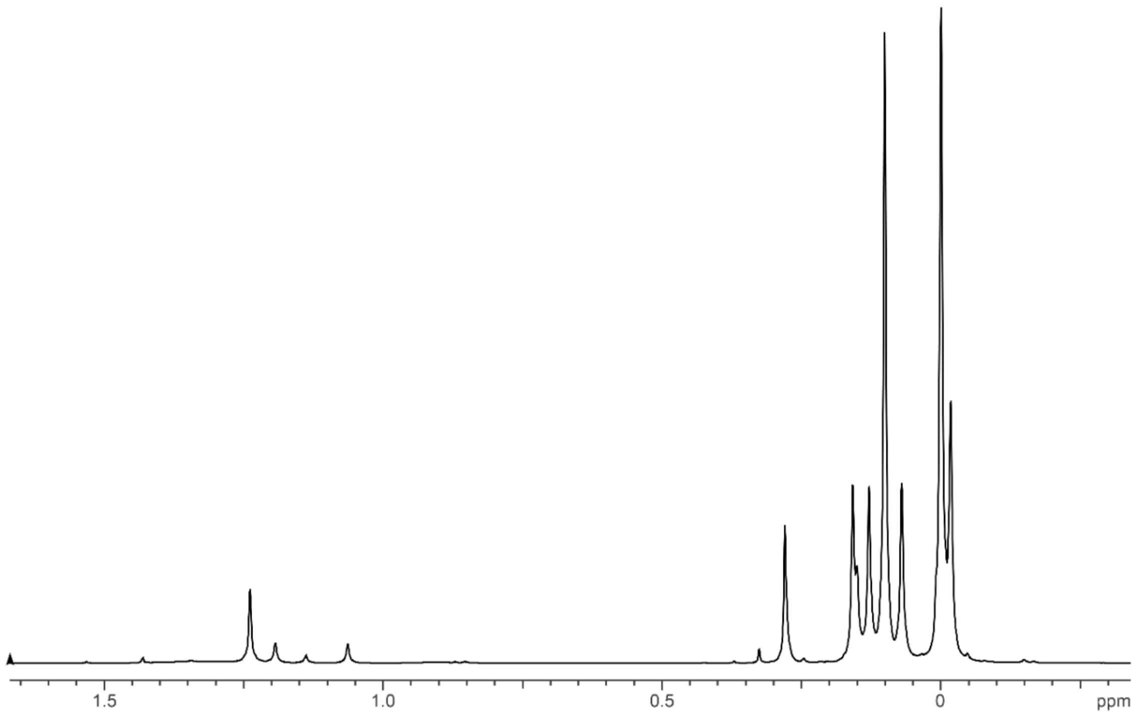

Figure S9: $^{1}H\{^{31}P\}$ NMR (400 MHz, 298 K, C_6D_6) spectrum of **1-Mg** in the region –0.5 – 1.5 ppm.

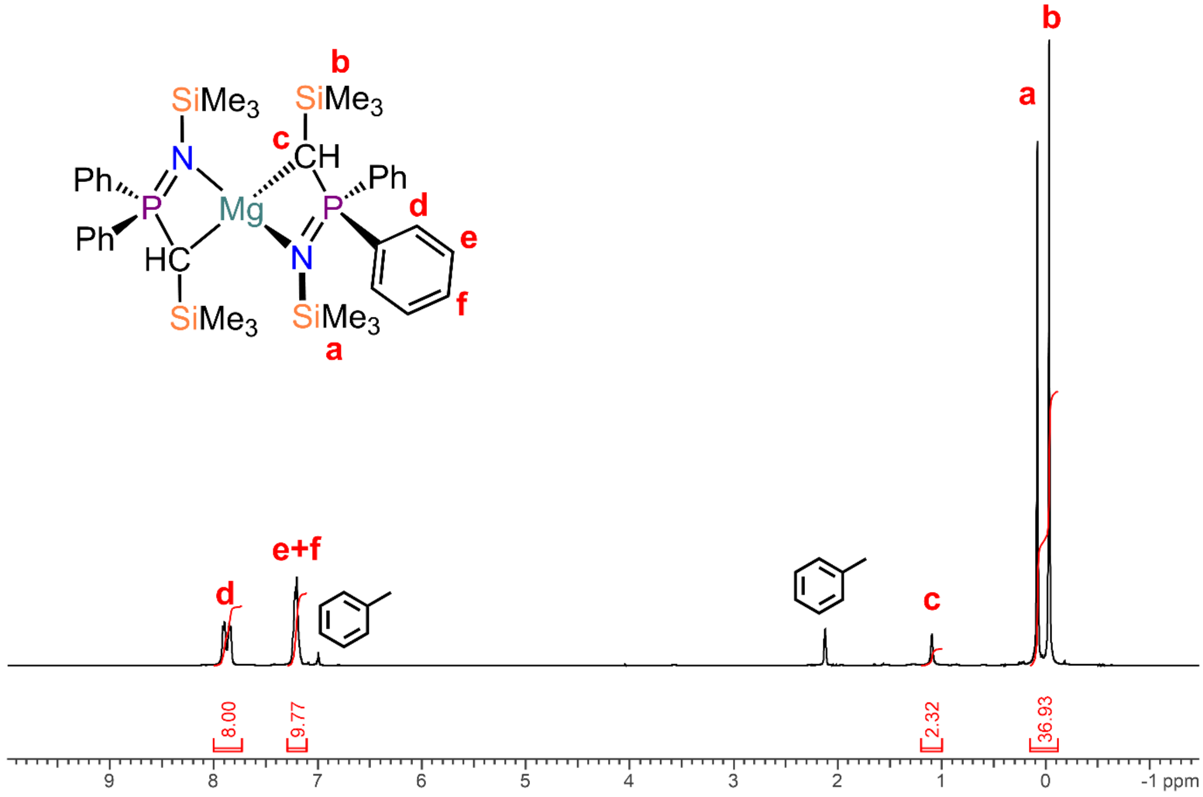

Figure S10: $^{1}H\{^{31}P\}$ NMR (400 MHz, 378 K, C_7D_8) spectrum of **1-Mg.**

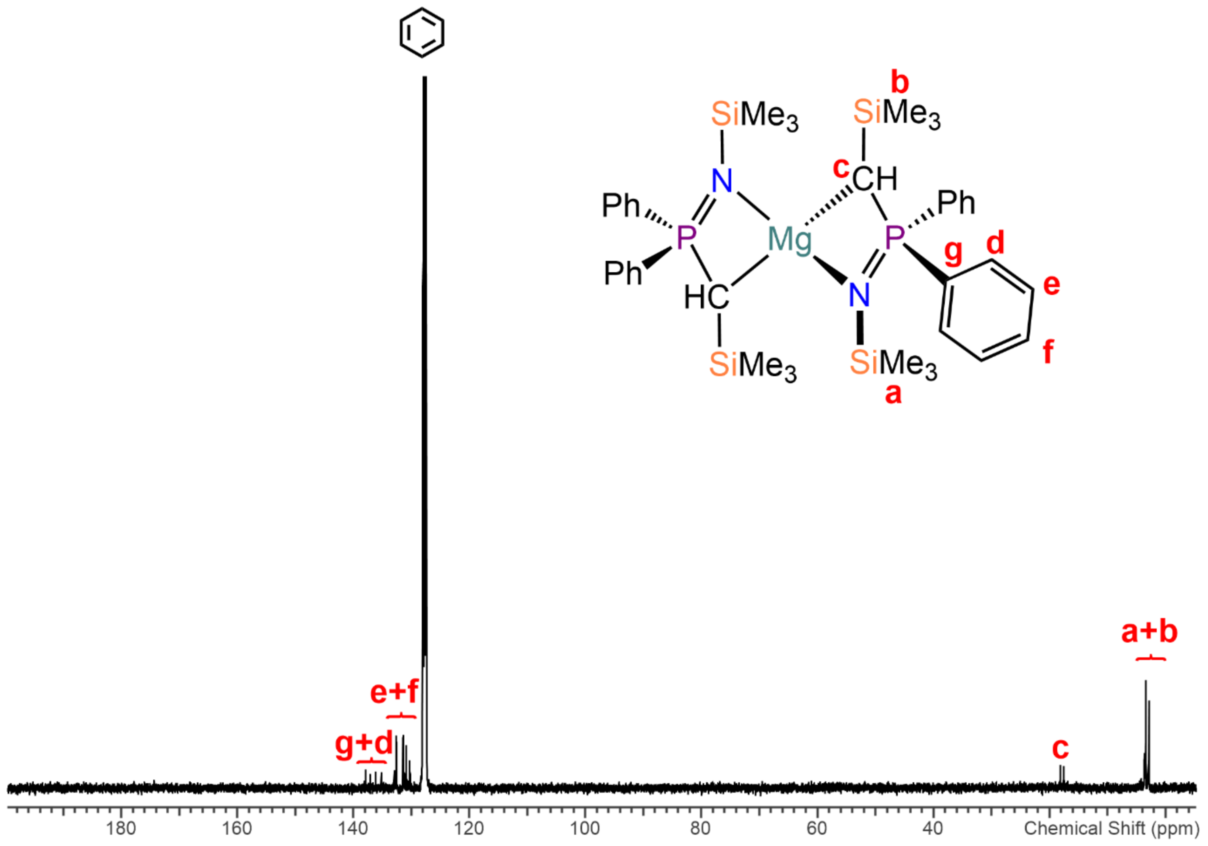

Figure S11: $^{13}C\{^{1}H\}$ NMR (101 MHz, 298 K, C_6D_6) spectrum of **1-Mg**, with assignment.

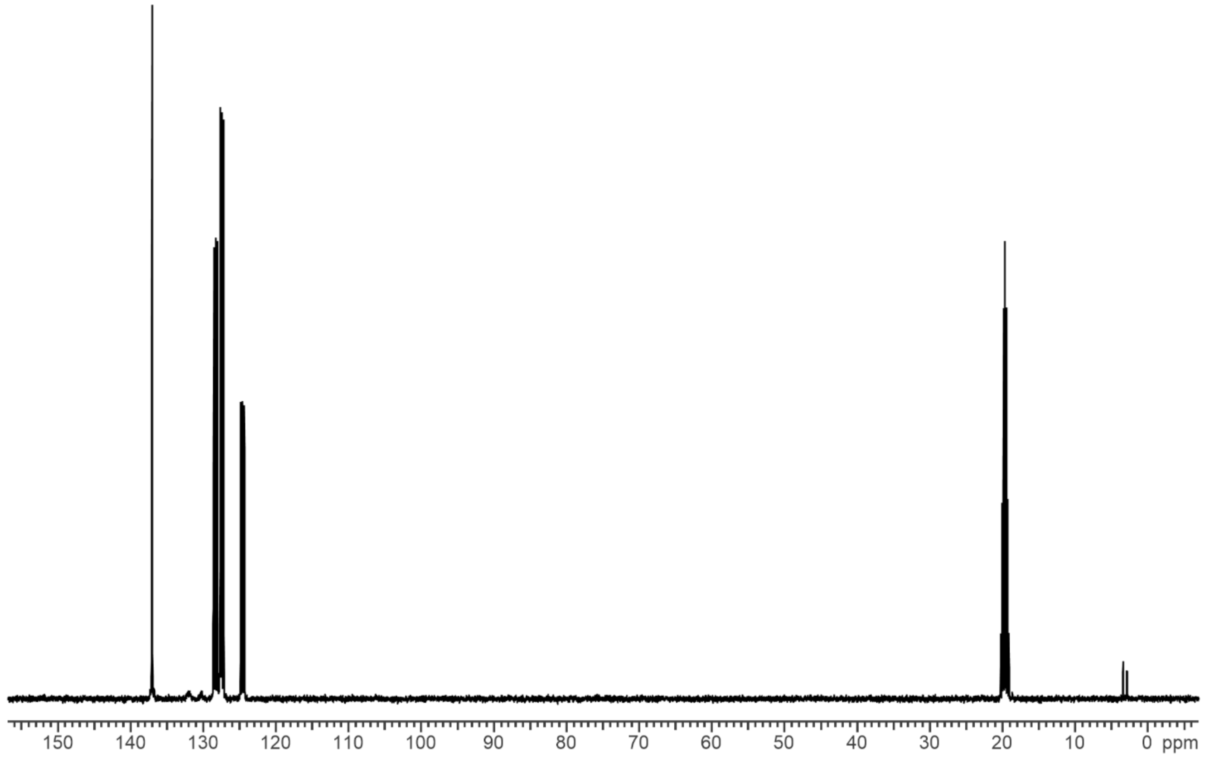

Figure S12: $^{13}C\{^{1}H\}$ NMR (101 MHz, C_7D_8) spectrum of **1-Mg**.

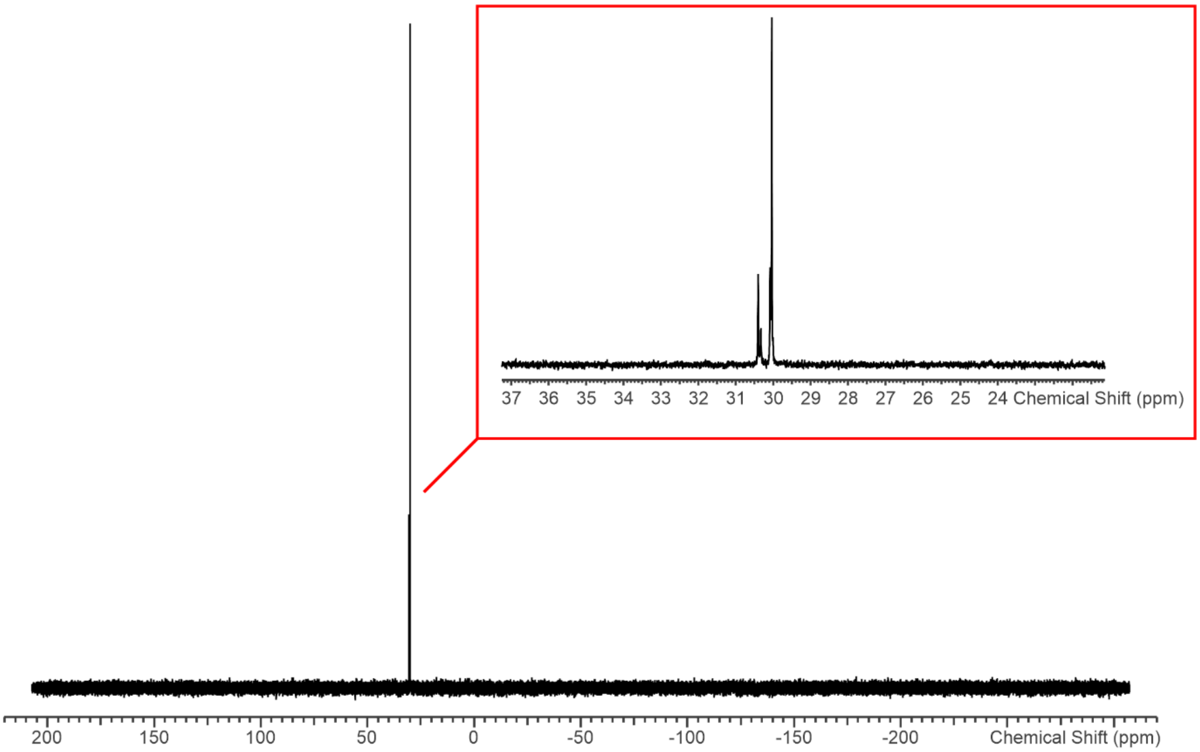

Figure S13a: $^{31}P\{^{1}H\}$ NMR (162 MHz, 298 K, C_6D_6) spectrum of **1-Mg**. Inset shows region between 20-37 ppm.

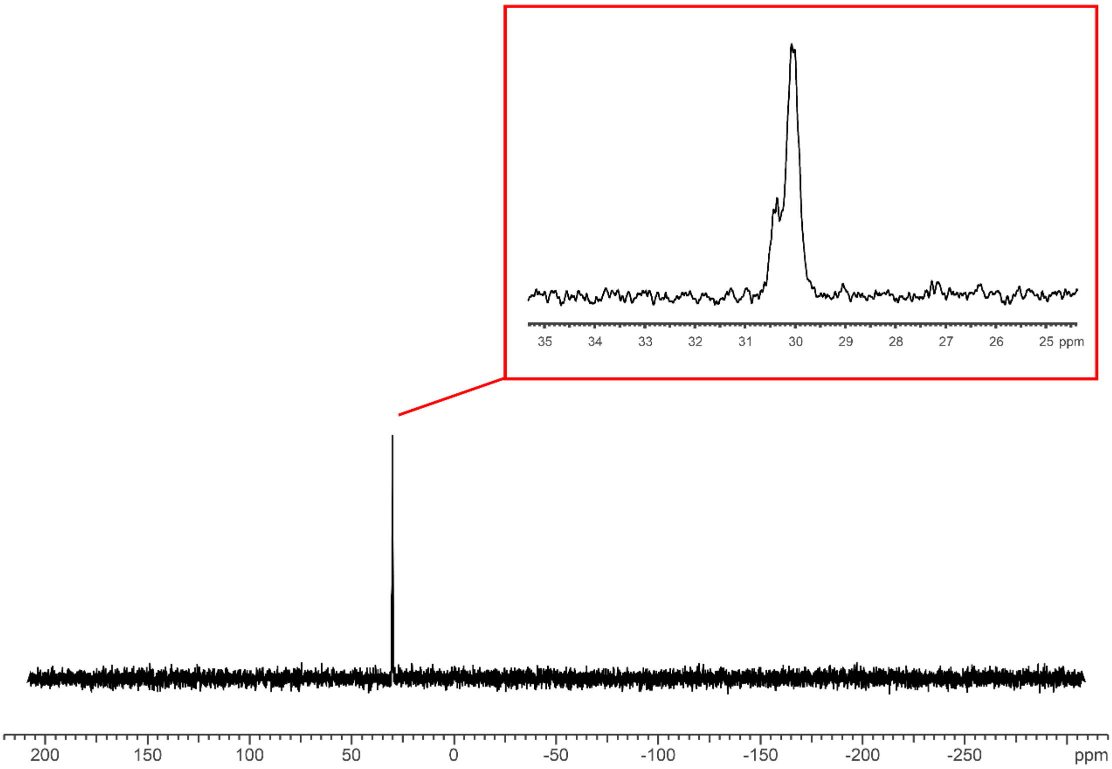

Figure S13b: ^{31}P NMR (162 MHz, 298 K, C_6D_6) spectrum of **1-Mg**. Inset shows region between 25-35 ppm.

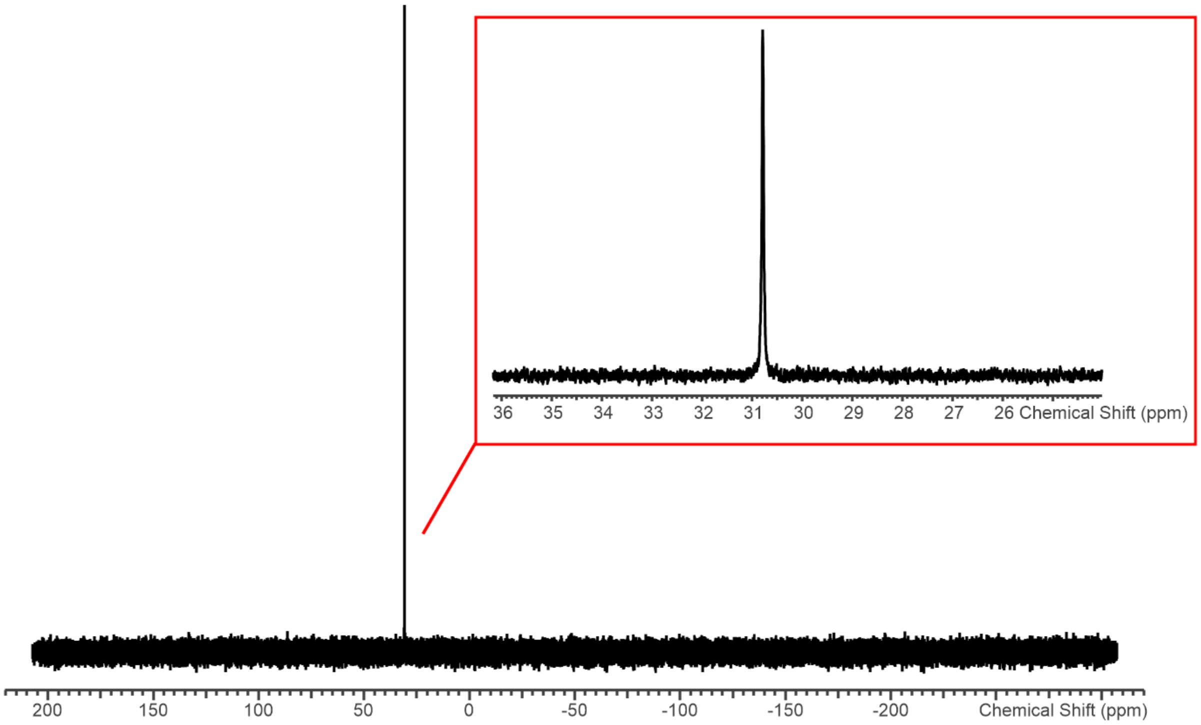

Figure S14: $^{31}P\{^1H\}$ NMR (162 MHz, 373 K, C_6D_6) spectrum of **1-Mg**. Inset shows region between 24-36 ppm.

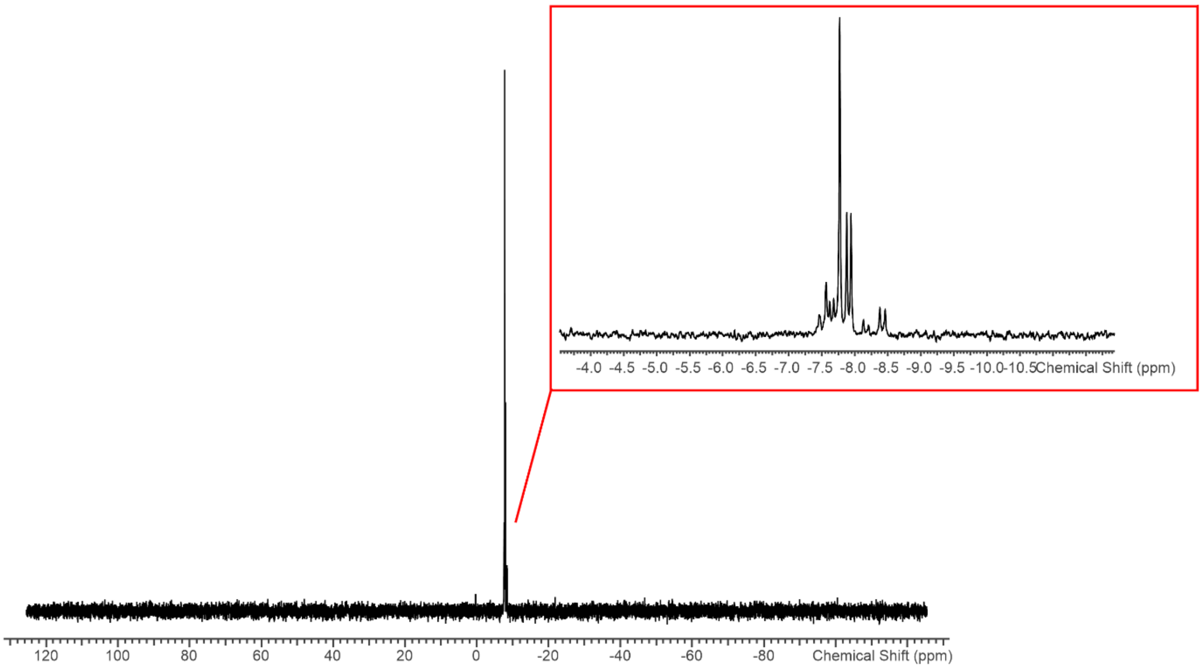

Figure S15: $^{29}Si\{^1H\}$ NMR (80 MHz, 298 K, C_6D_6) spectrum of **1-Mg**. Inset shows region between –10.5 and –4 ppm.

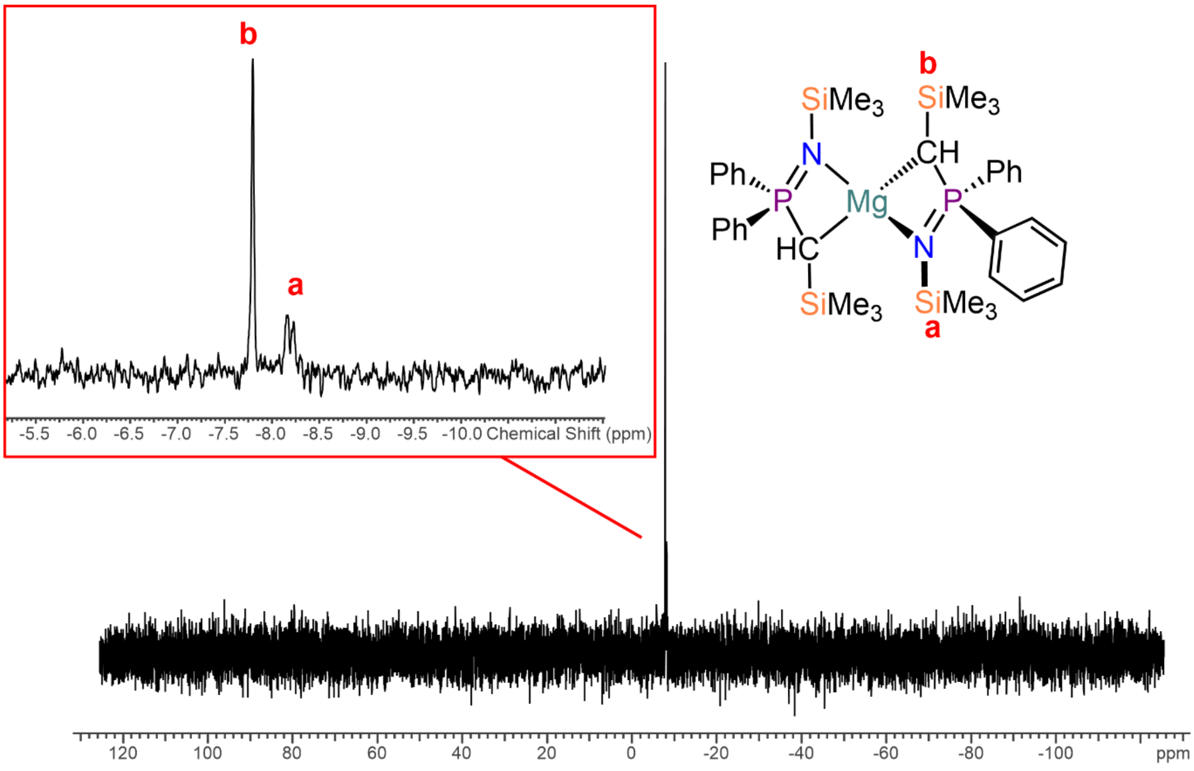

Figure S16: $^{29}Si\{^1H\}$ NMR (80 MHz, 378 K, C_6D_6) spectrum of **1-Mg**. Inset shows region between –12 and –5.5 ppm.

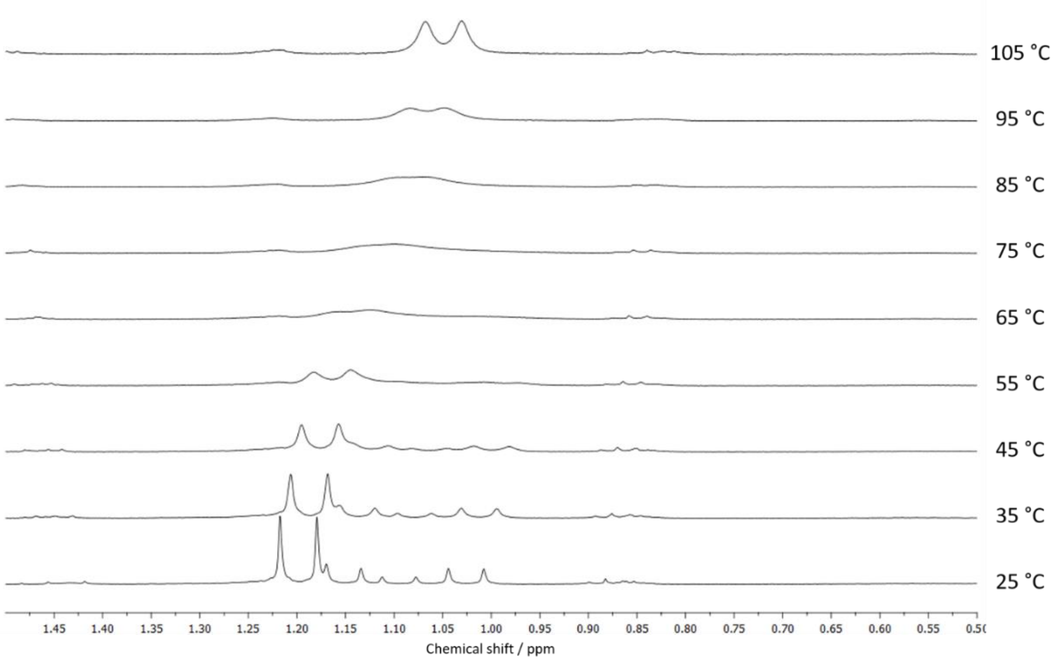

Figure S17: ^1H VT NMR study of **1-Mg** at 25 – 105 °C in d_8-toluene in the region 0.5 – 1.5 ppm.

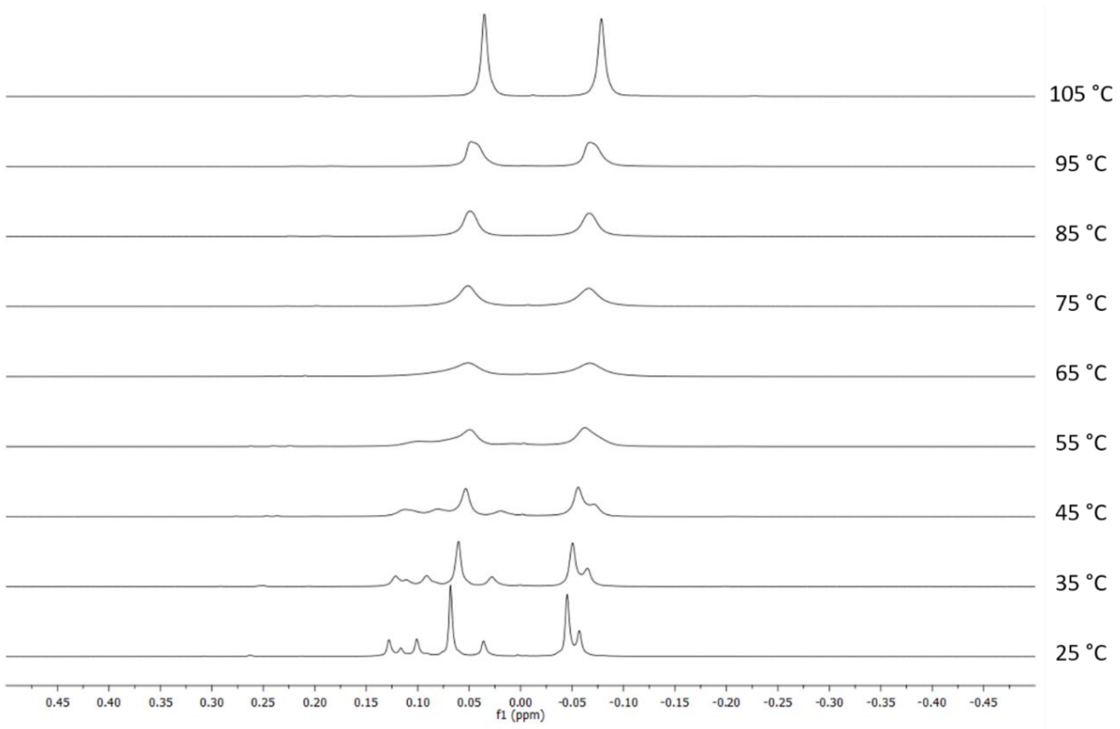

Figure S18: ^{1}H VT NMR study of **1-Mg** at 25 – 105 °C in d_8-toluene in the region ± 0.5 ppm.

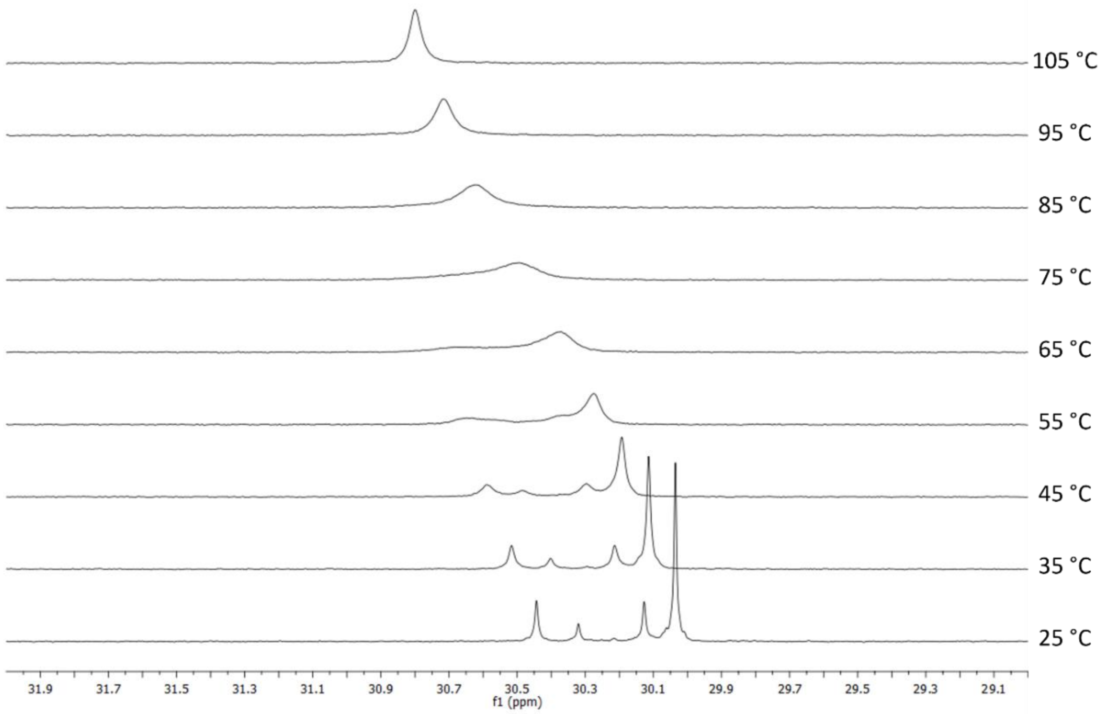

Figure 19: $^{31}P\{^{1}H\}$ VT NMR study of **1-Mg** at 25 – 105 °C in d_8-toluene in the region 30 – 32 ppm.

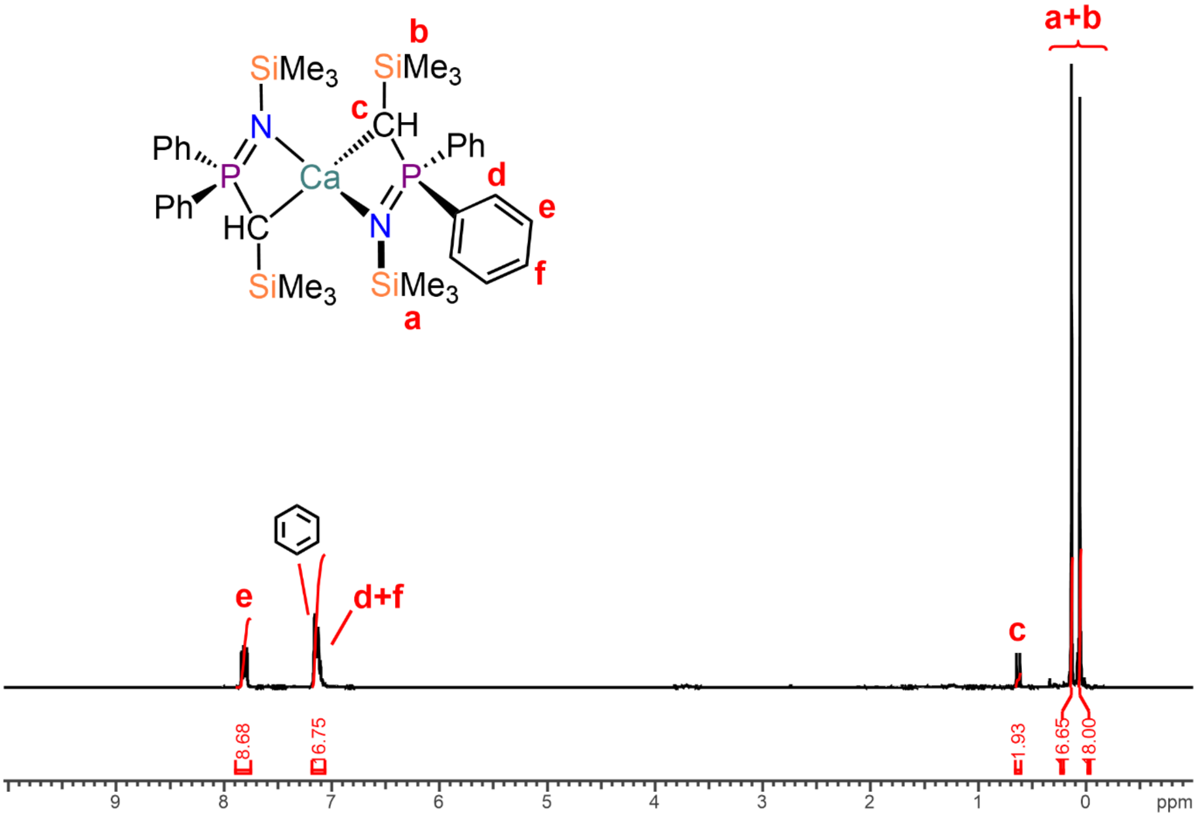

Figure S20: ^{1}H NMR (400 MHz, 298 K, C_6D_6) spectrum of **1-Ca**, with assignment.

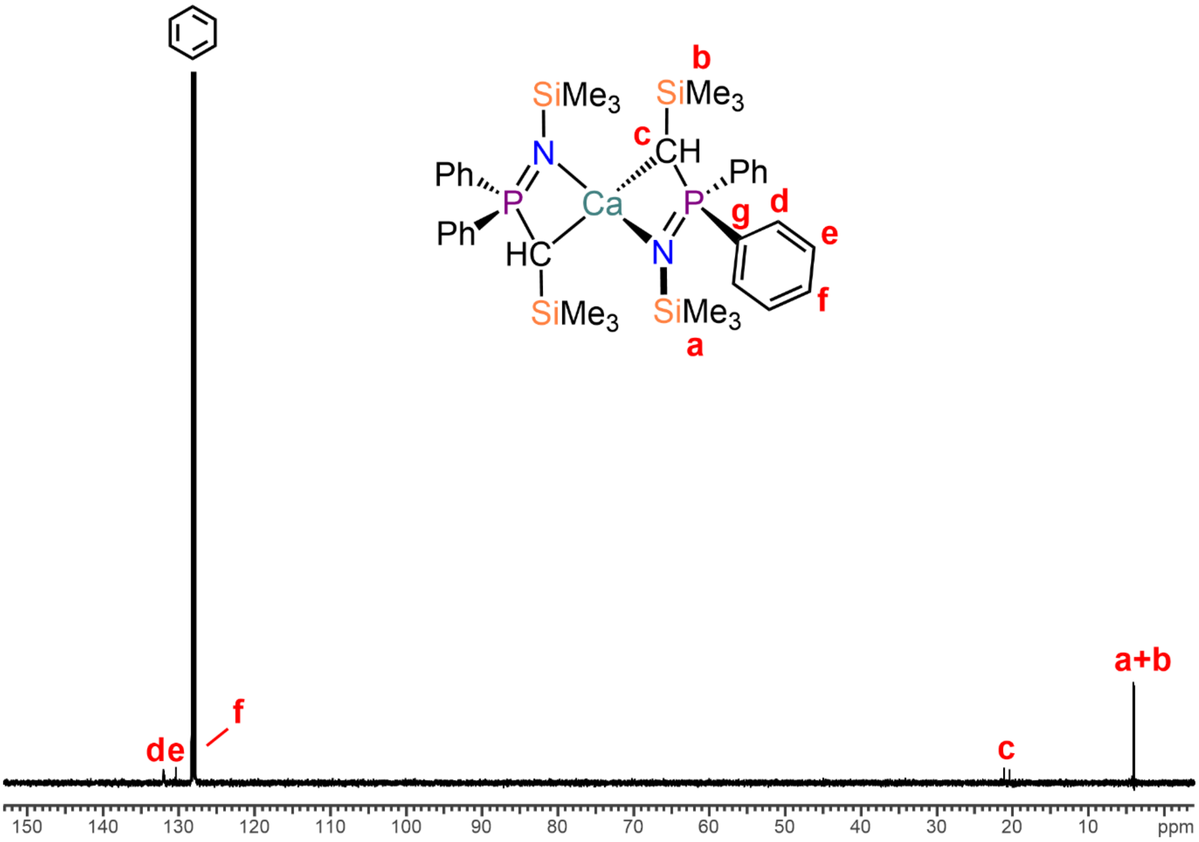

Figure S21: ^{13}C{^{1}H} NMR (101 MHz, 298 K, C_6D_6) spectrum of **1-Ca**, with assignment.

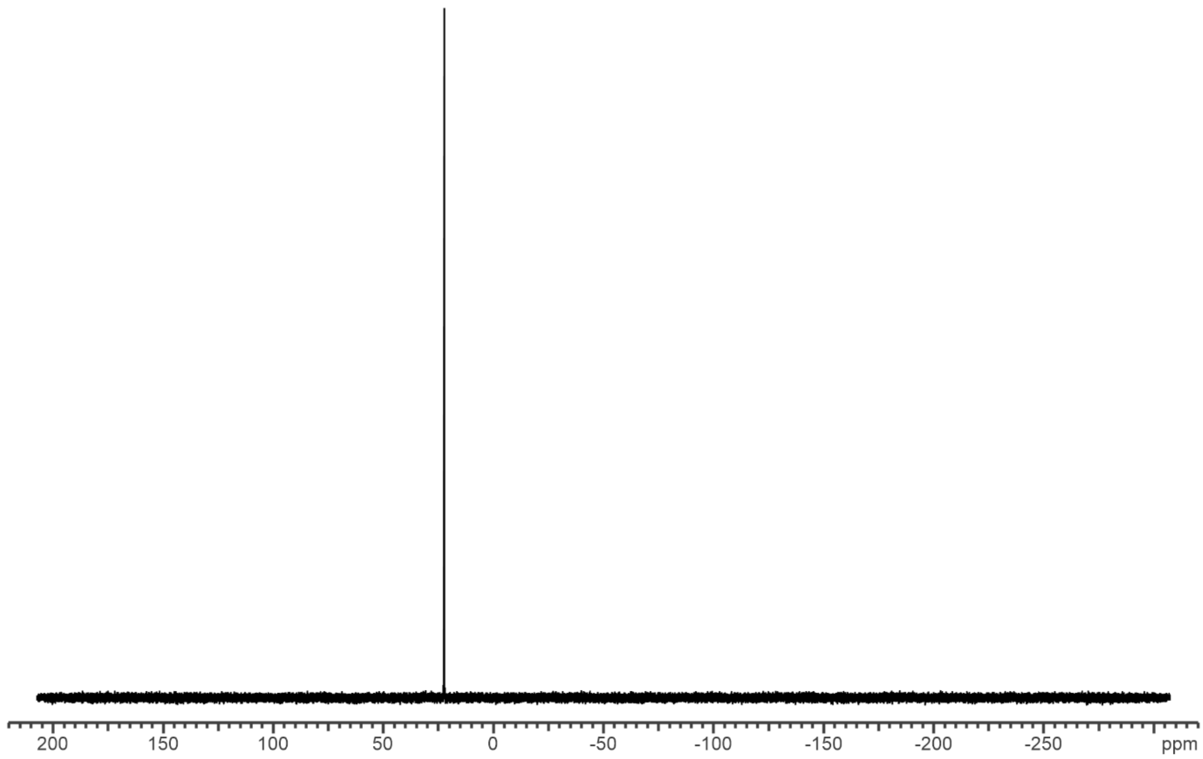

Figure S22: $^{31}P\{^{1}H\}$ NMR (162 MHz, 298 K, C_6D_6) spectrum of **1-Ca**.

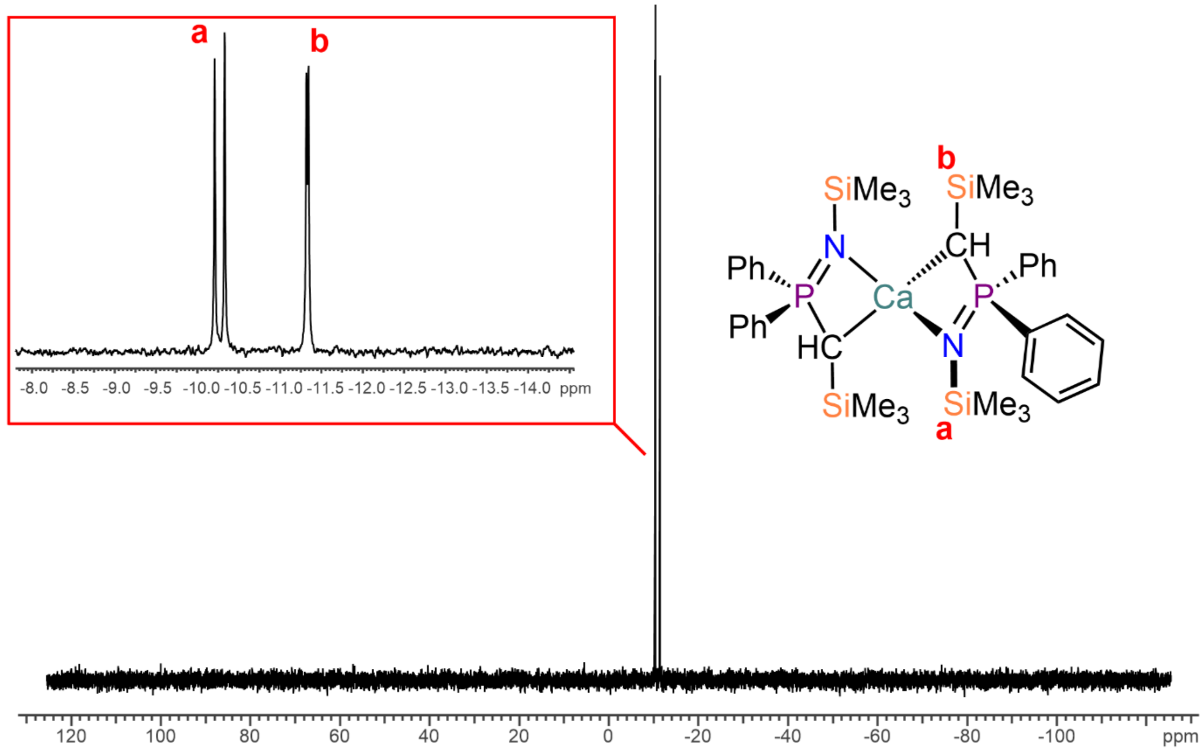

Figure S23: $^{29}Si\{^{1}H\}$ NMR (80 MHz, 298 K, C_6D_6) spectrum of **1-Ca**. Inset shows region between –14 and –8 ppm.

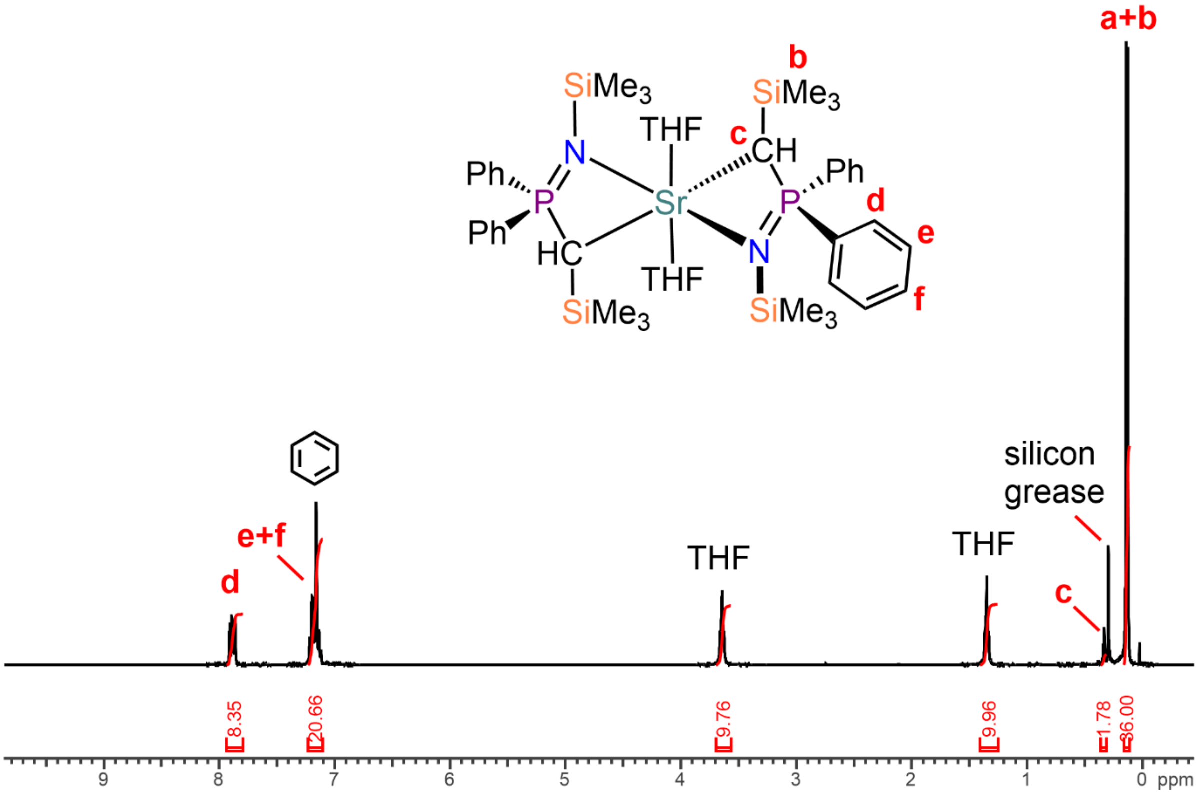

Figure S24: ^{1}H NMR (400 MHz, 298 K, C_6D_6) spectrum of **1-Sr·(THF)$_2$**, with assignment.

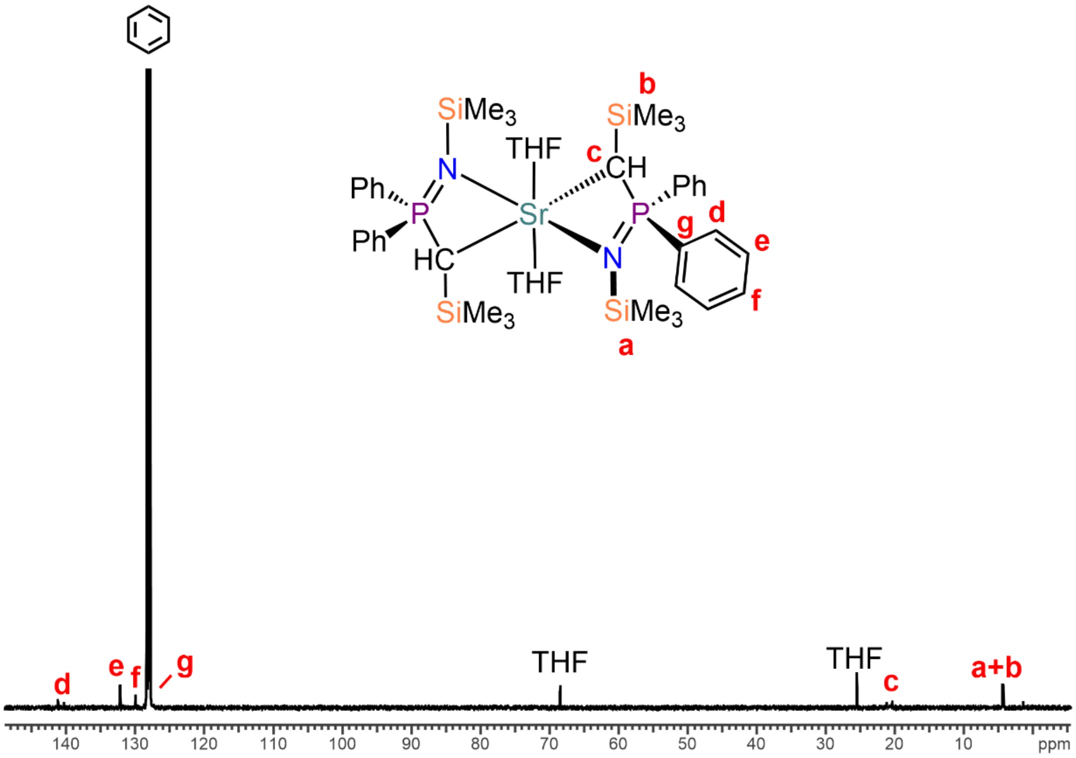

Figure S25: ^{13}C{^{1}H} NMR (101 MHz, 298 K, C_6D_6) spectrum of **1-Sr·(THF)$_2$**, with assignment.

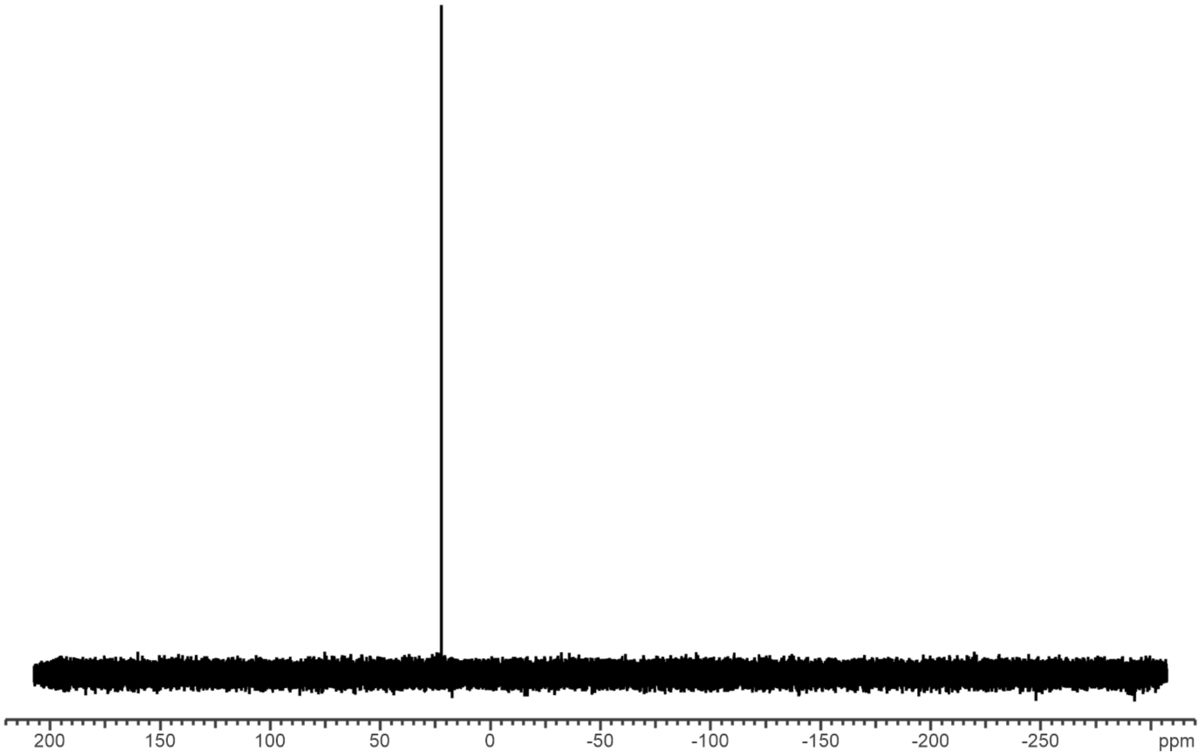

Figure S26: $^{31}P\{^1H\}$ NMR (162 MHz, 298 K, C_6D_6) spectrum of **1-Sr·(THF)₂**.

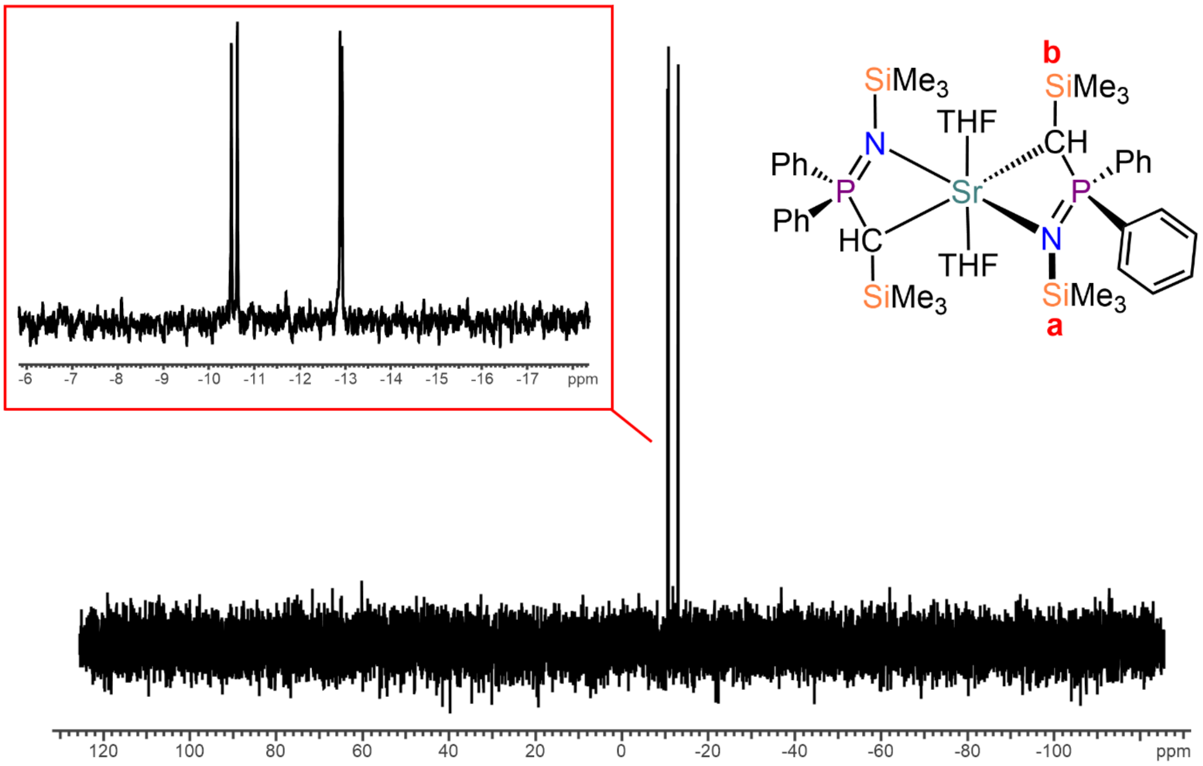

Figure S27: $^{29}Si\{^1H\}$ NMR (80 MHz, 298 K, C_6D_6) spectrum of **1-Sr·(THF)₂**, with assignment. Inset shows region between –18 and –6 ppm.

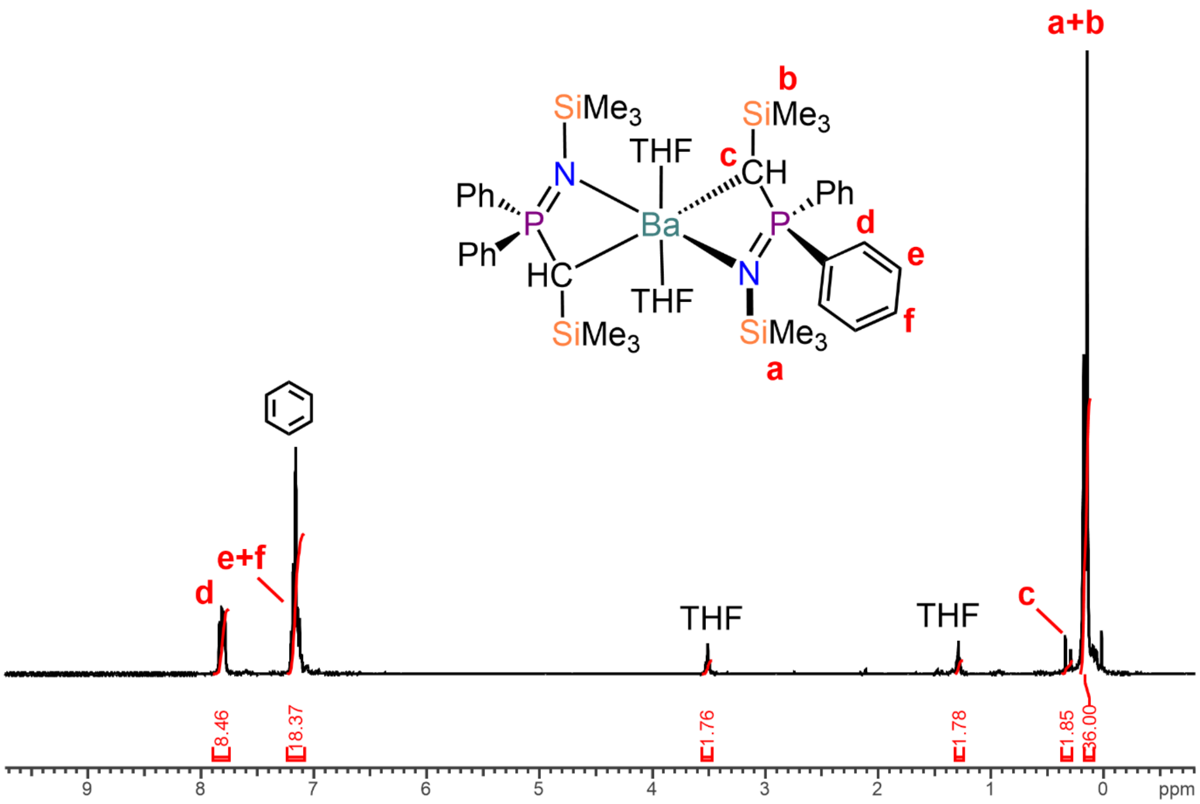

Figure S28: ^{1}H NMR (400 MHz, 298 K, C_6D_6) spectrum of **1-Ba·(THF)$_2$**, with assignment.

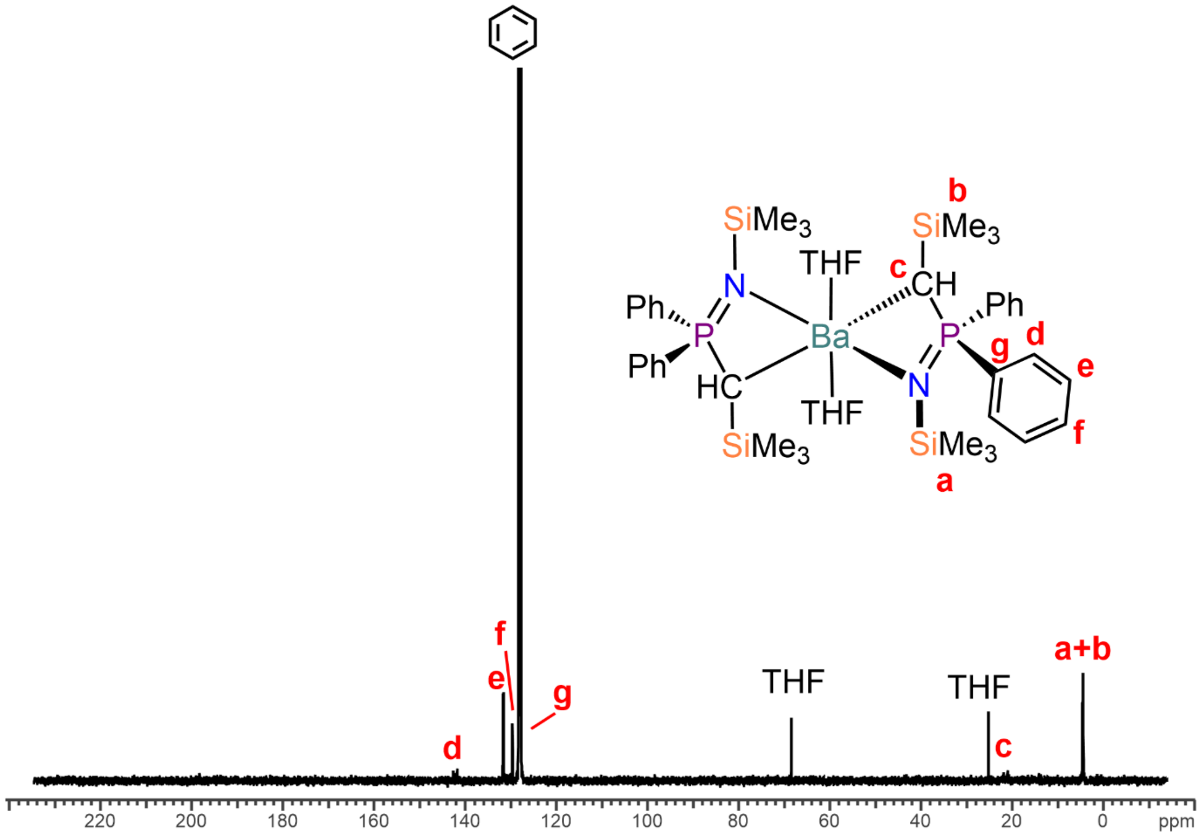

Figure S29: $^{13}C\{^{1}H\}$ NMR (101 MHz, 298 K, C_6D_6) spectrum of **1-Ba·(THF)₂**, with assignment.

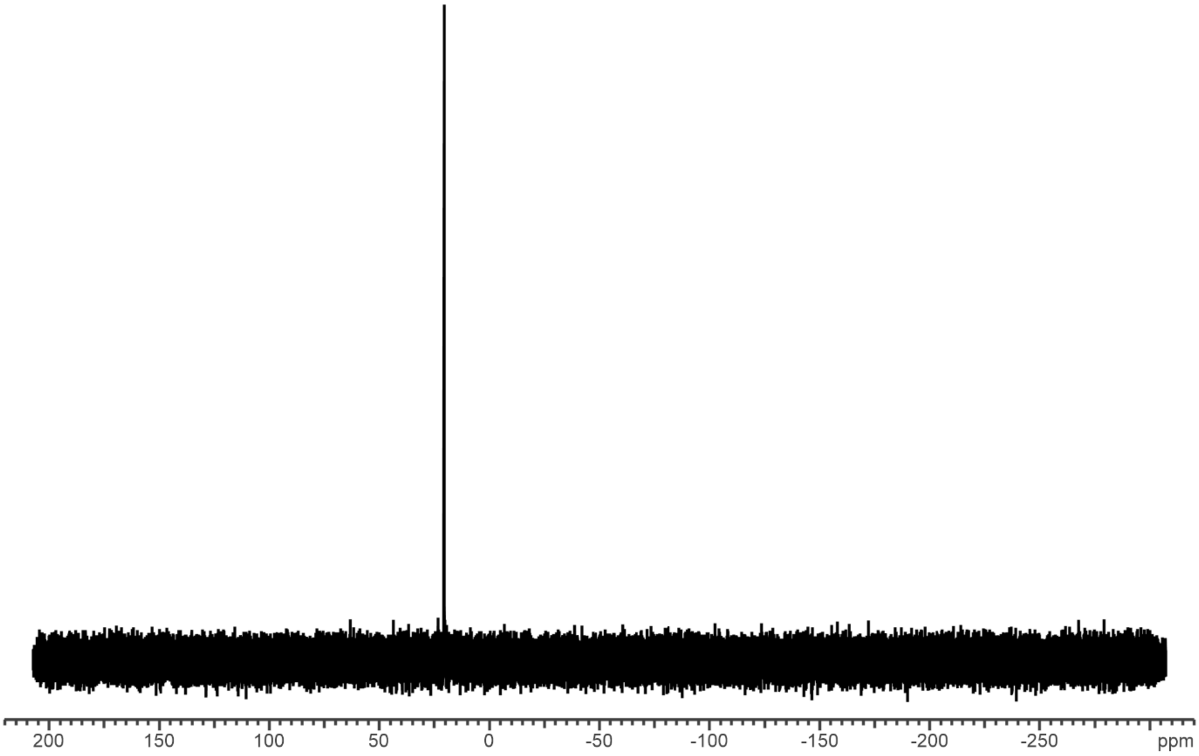

Figure S30: $^{31}P\{^{1}H\}$ NMR (162 MHz, 298 K, C_6D_6) spectrum of **1-Ba·(THF)$_2$**.

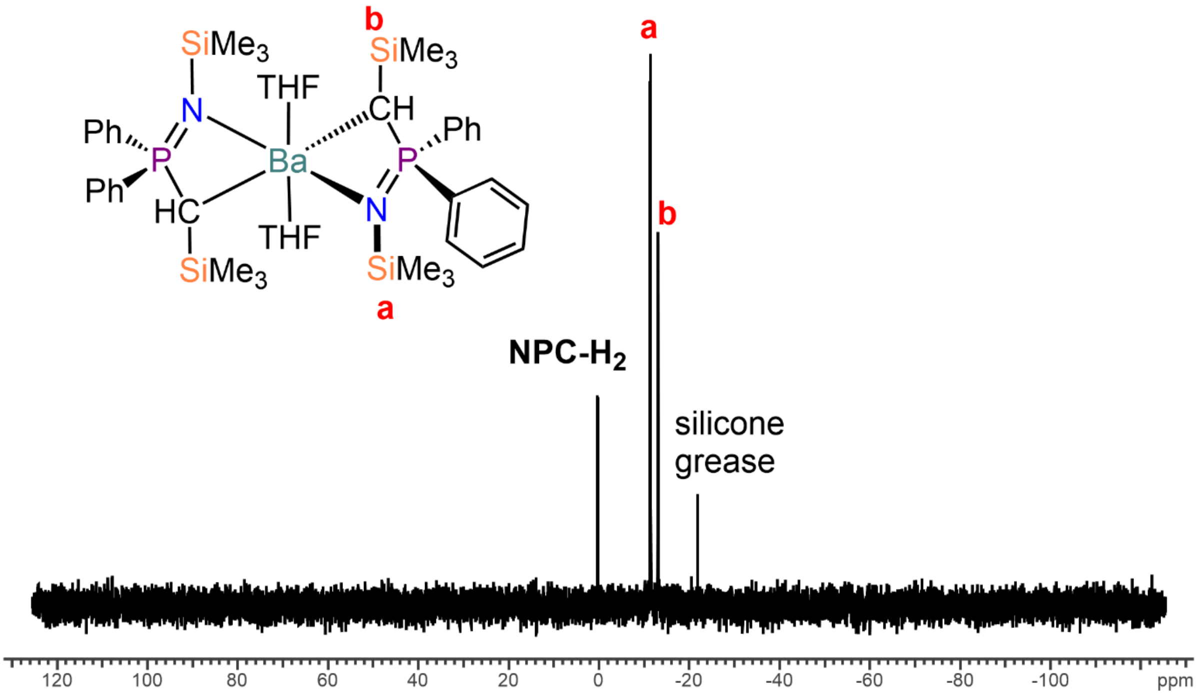

Figure S31: $^{29}Si\{^{1}H\}$ NMR (80 MHz, 298 K, C_6D_6) spectrum of **1-Ba·(THF)$_2$**.

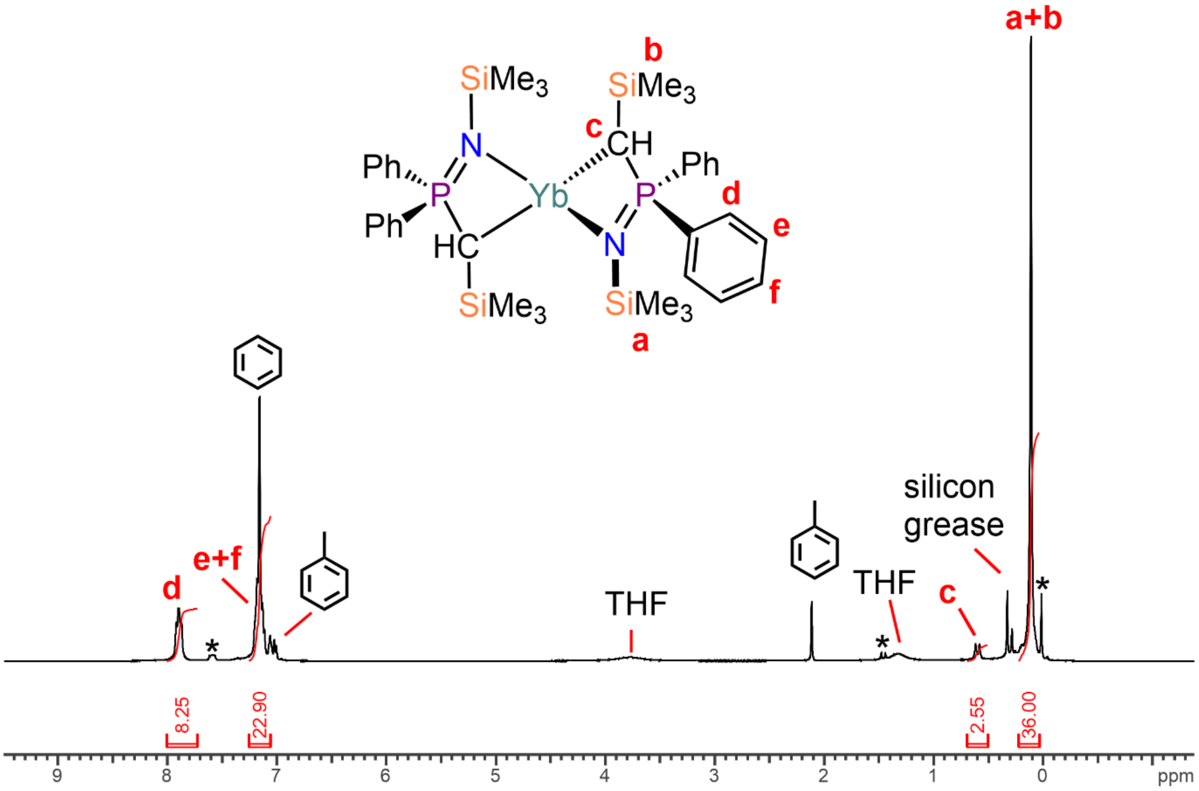

Figure S32: ^{1}H NMR (400 MHz, 298 K, C_6D_6/C_4D_8O) spectrum of **1-Yb**, with assignment. * denotes proligand NPC-H_2.

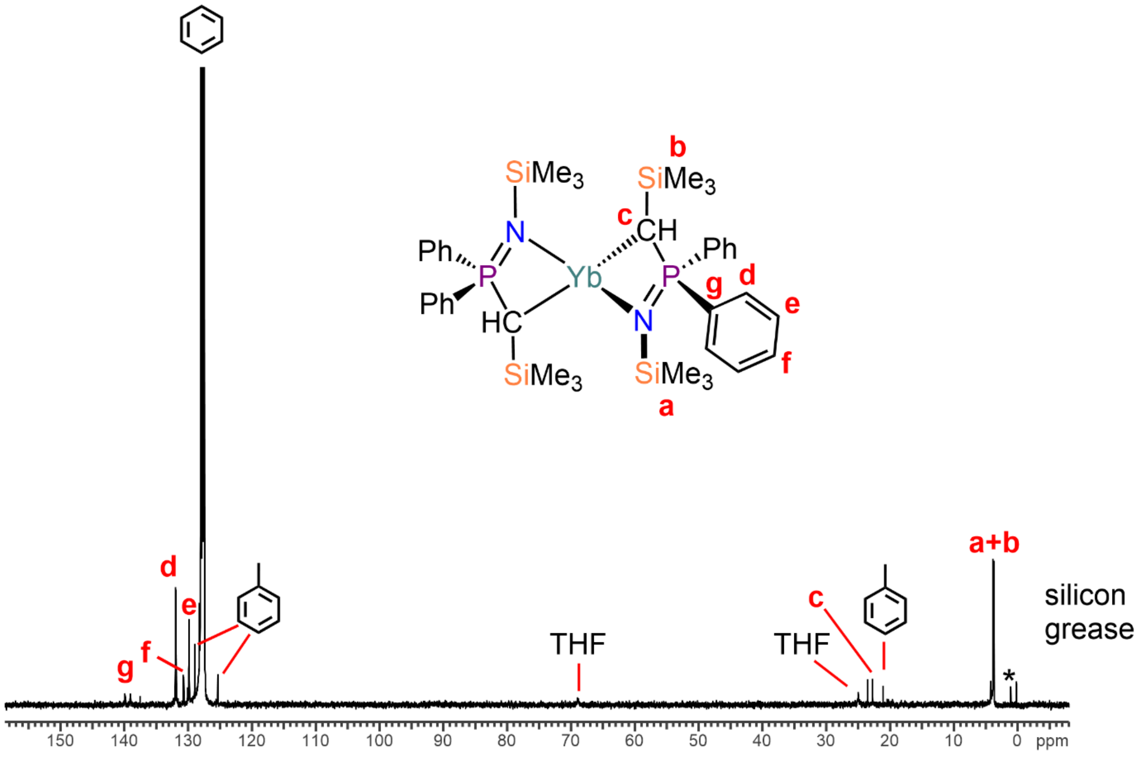

Figure S33: $^{13}C\{^{1}H\}$ NMR (101 MHz, 298 K, C_6D_6/C_4D_8O) spectrum of **1-Yb**, with assignment. * denotes proligand NPC-H_2

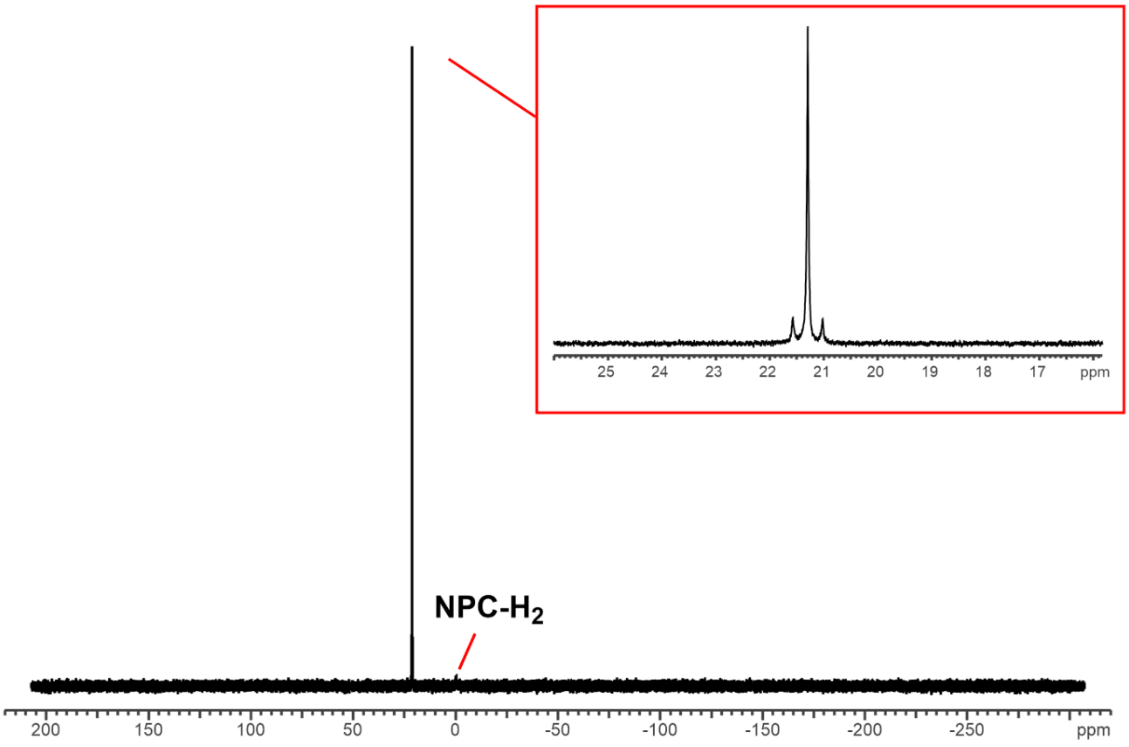

Figure S34: $^{31}P\{^{1}H\}$ NMR (162 MHz, 298 K, C_6D_6/C_4D_8O) spectrum of **1-Yb**. Inset shows the region between 18 and 25 ppm.

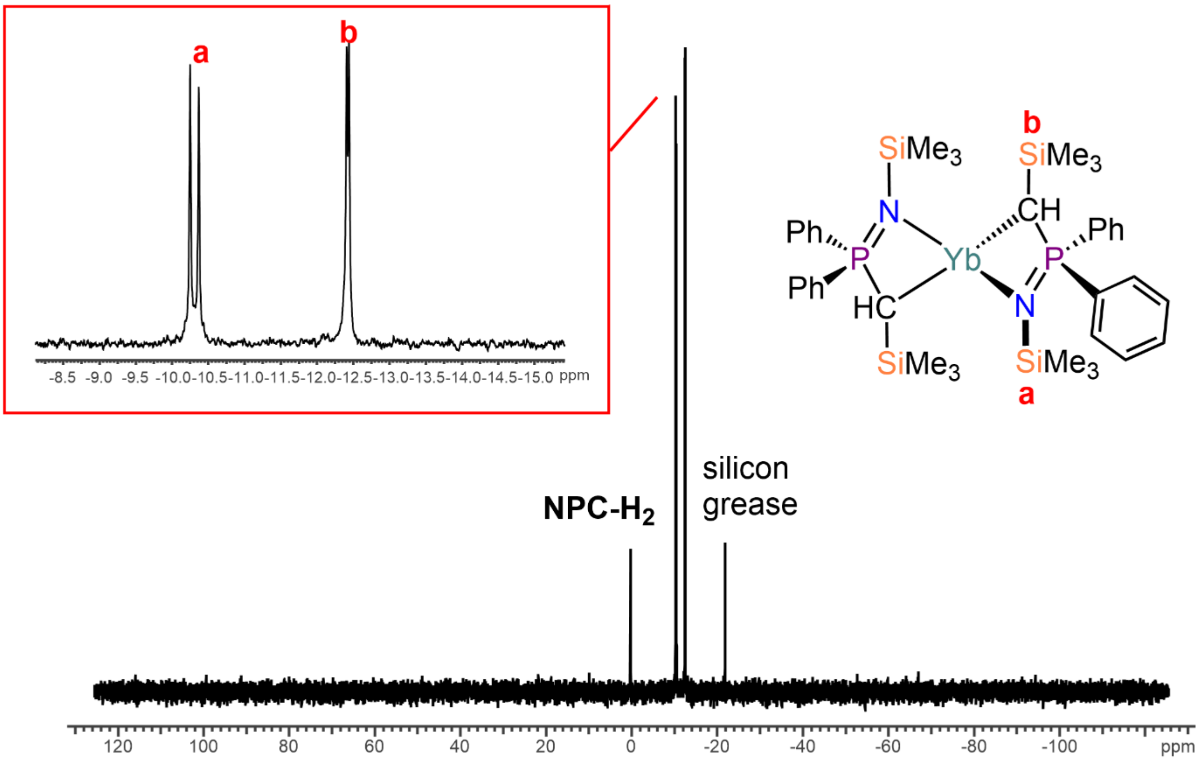

Figure S35: $^{29}Si\{^{1}H\}$ NMR (80 MHz, 298 K, C_6D_6/C_4D_8O) spectrum of **1-Yb**, with assignment. Inset shows region between –15 and –18.5 ppm.

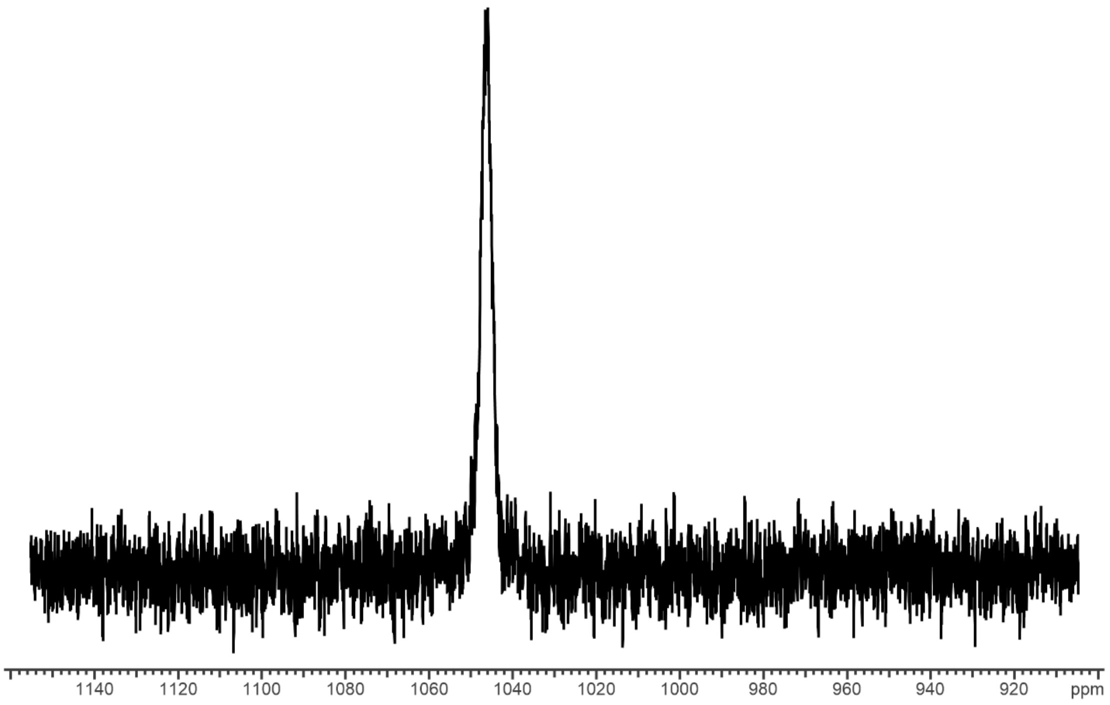

Figure S36: $^{171}Yb\{^{1}H\}$ NMR (88 MHz, 298 K, C_6D_6) spectrum of **1-Yb**.

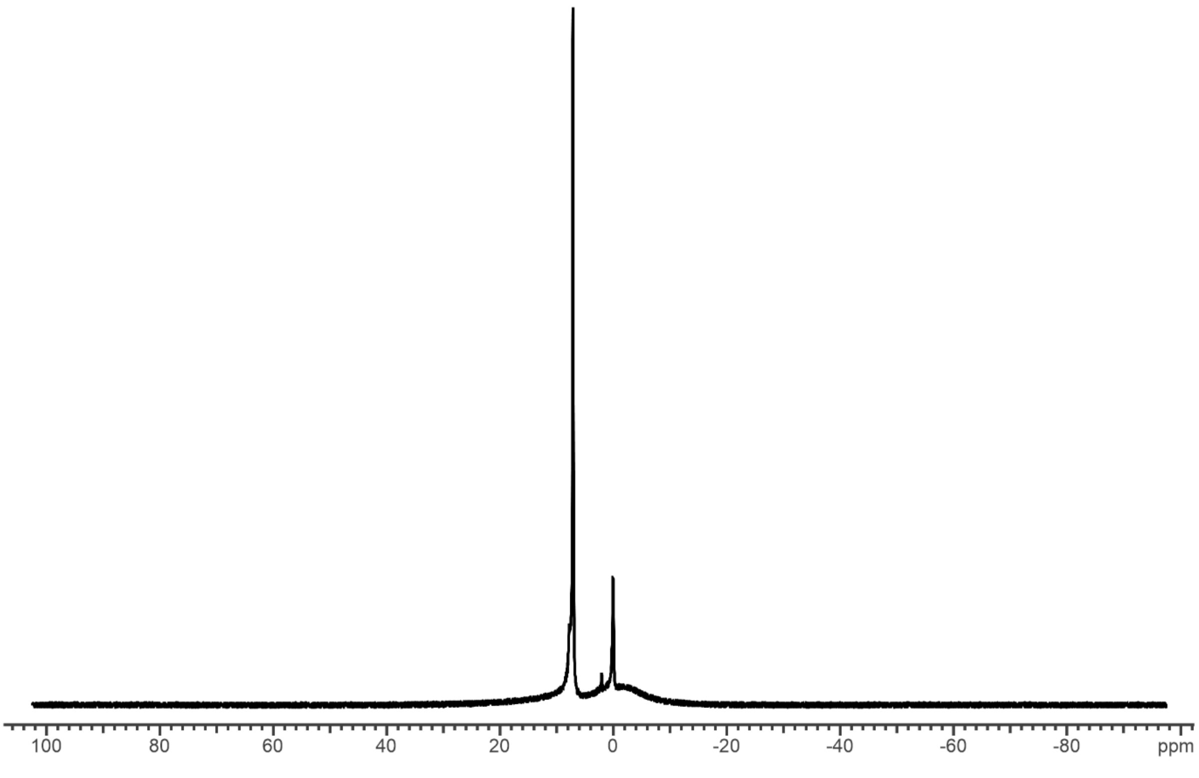

Figure S37: ^{1}H NMR (400 MHz, 298 K, C_6D_6) spectrum of **1-Eu**.

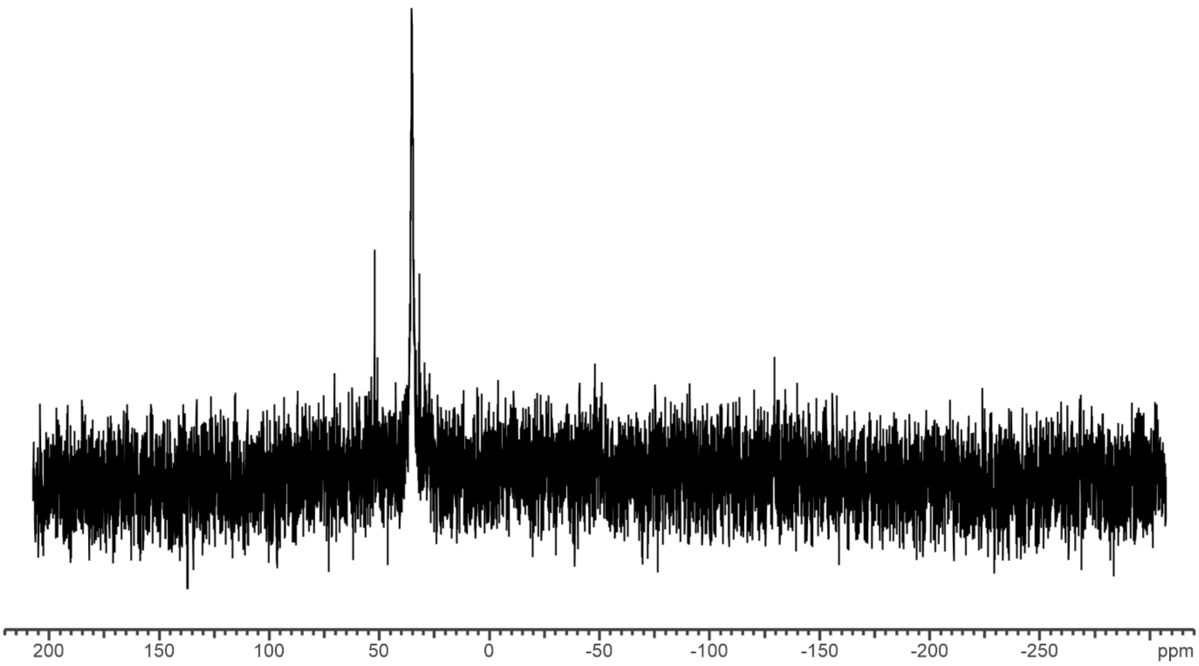

Figure S38: $^{31}P\{^{1}H\}$ NMR (162 MHz, 298 K, C_6D_6) spectrum of **1-Eu**. An exponential broadening post-processing function of 10 Hz was applied to improve the signal-to-noise ratio.

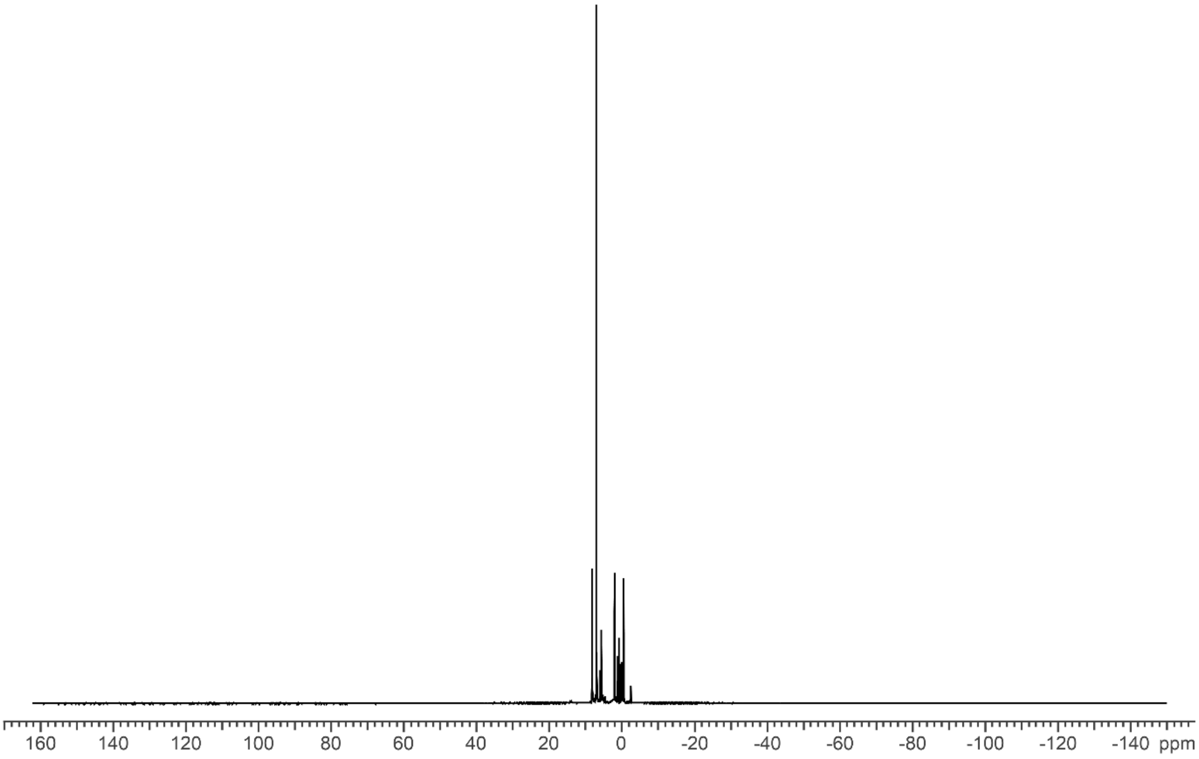

Figure S39: ^{1}H NMR (400 MHz, 298 K, C_6D_6) spectrum of **1-Sm·(THF)$_2$**.

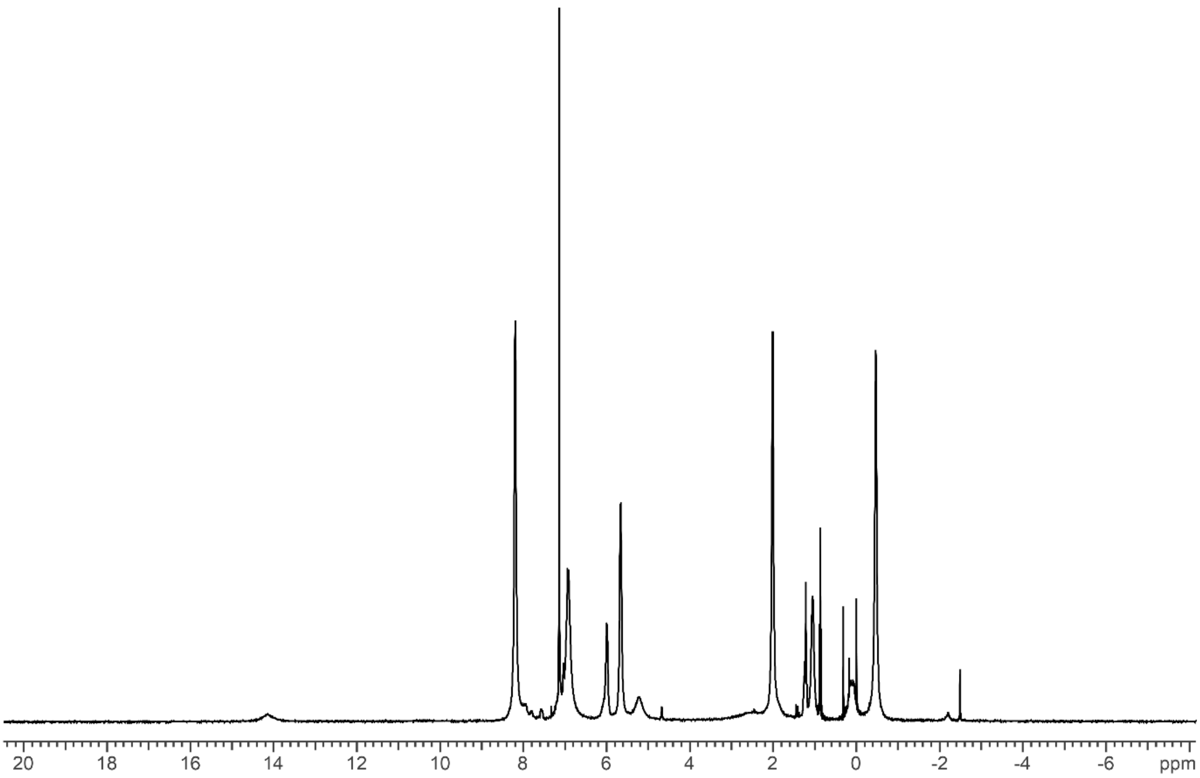

Figure S40: ^{1}H NMR (400 MHz, 298 K, C_6D_6) spectrum of **1-Sm·(THF)$_2$** zoomed in the region between –7 and 20 ppm.

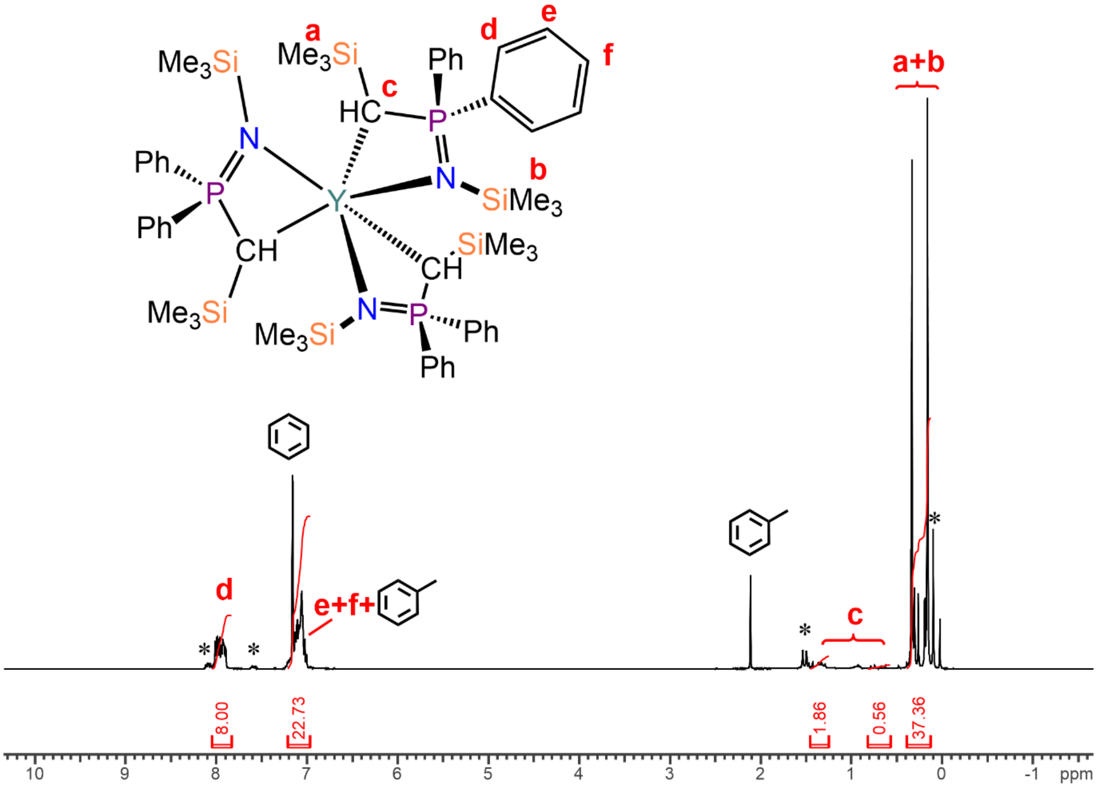

Figure S41: ^{1}H NMR (400 MHz, 298 K, C_6D_6) spectrum of **2-Y**, with assignment. * denotes proligand **NPC-H$_2$.**

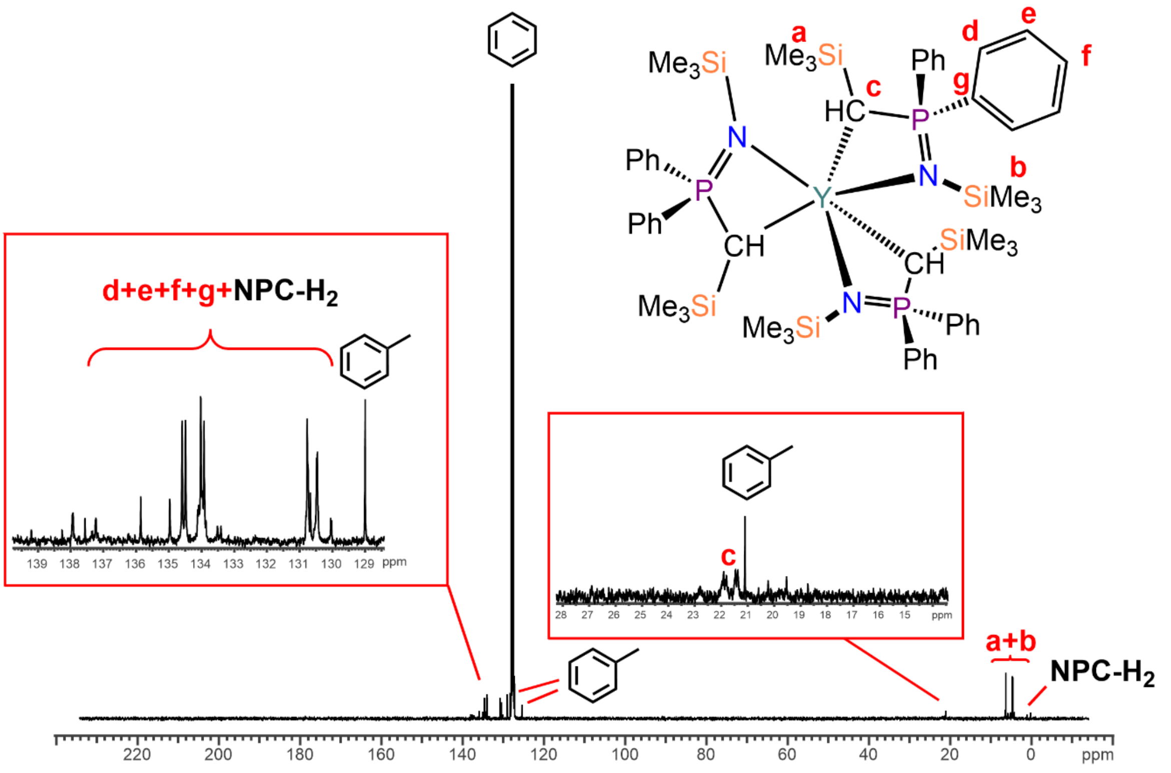

Figure S42: $^{13}C\{^1H\}$ NMR (101 MHz, 298 K, C_6D_6) spectrum of **2-Y**, with assignment.

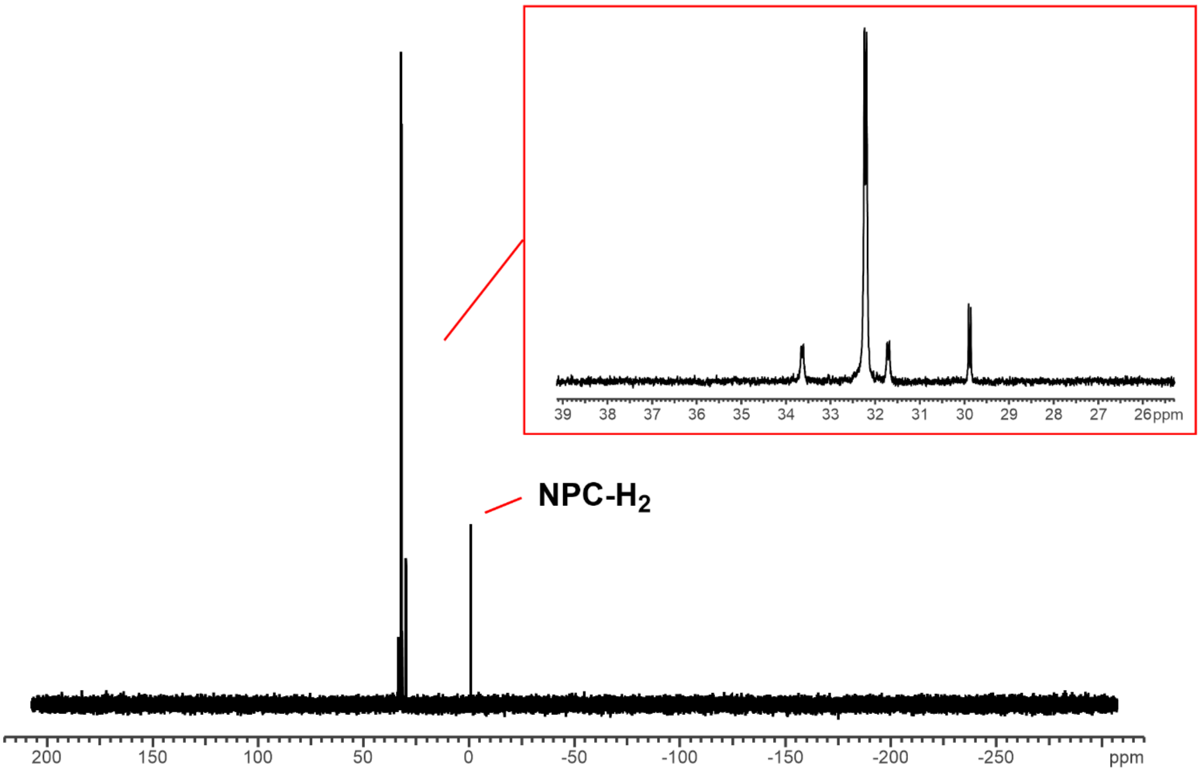

Figure S43: $^{31}P\{^1H\}$ NMR (162 MHz, 298 K, C_6D_6) spectrum of **2-Y**.

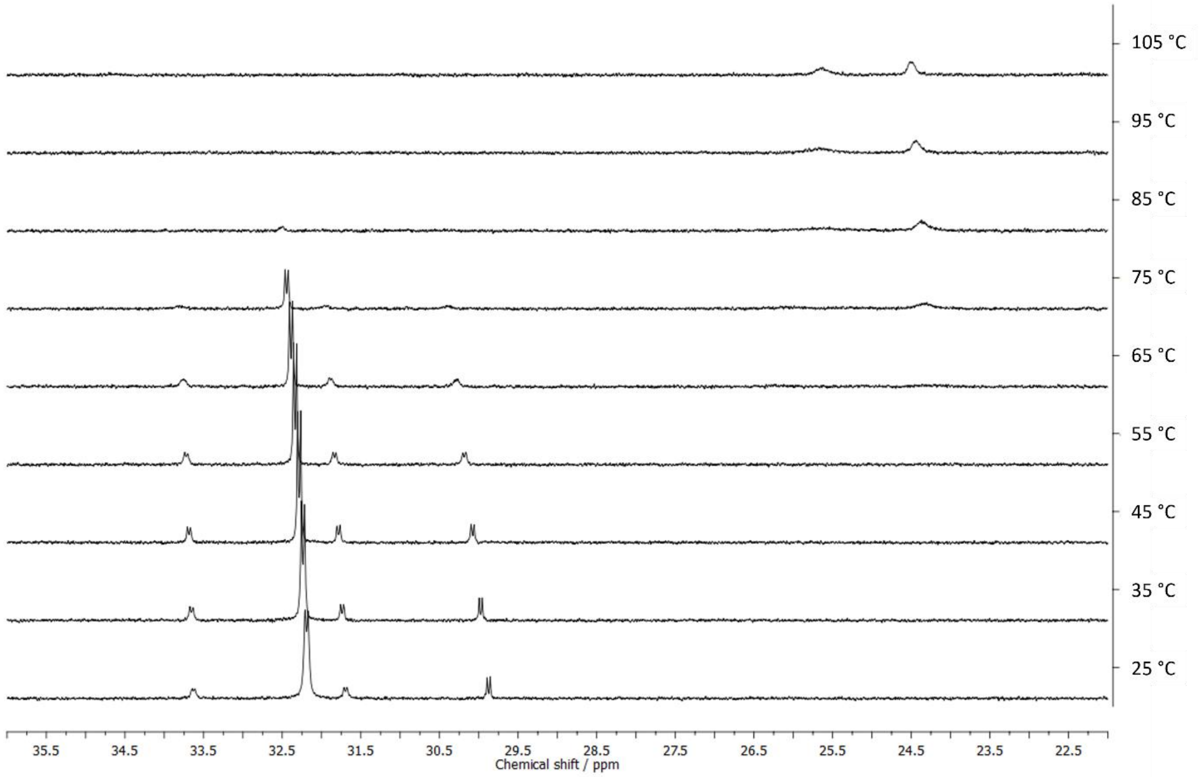

Figure S44: $^{31}P\{^{1}H\}$ VT-NMR study of **2-Y** at 25 – 105 °C in d_8-toluene in the region 22.0 – 36.0 ppm.

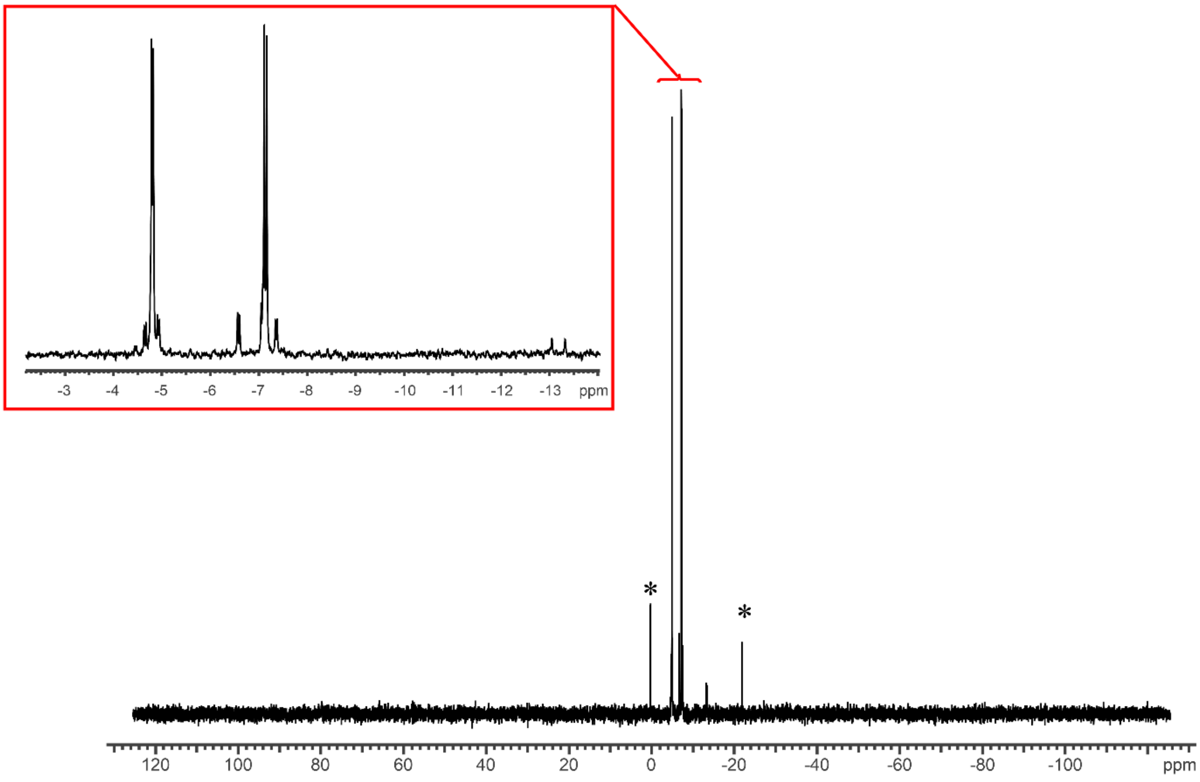

Figure S45: $^{29}Si\{^{1}H\}$ NMR (80 MHz, 298 K, C_6D_6) spectrum of **2-Y**. * denotes proligand **NPC-H$_2$**.

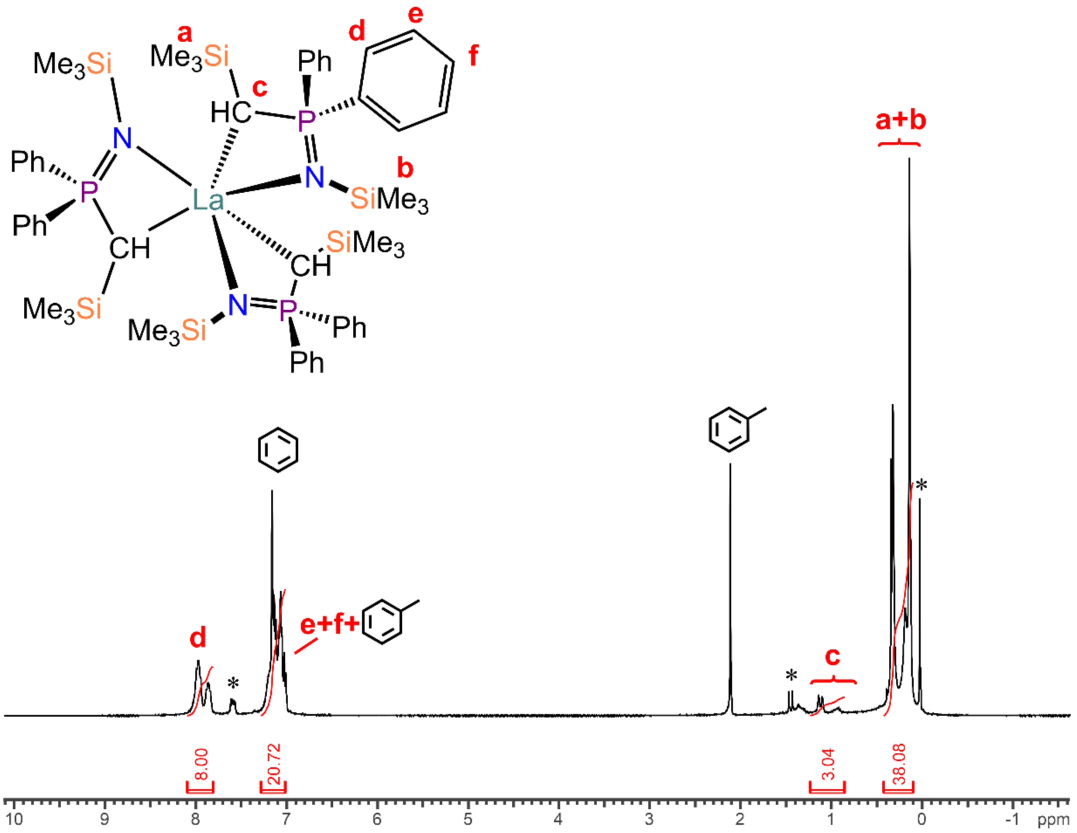

Figure S46: ^{1}H NMR (400 MHz, 298 K, C_6D_6) spectrum of **2-La** with assignment. * denotes proligand **NPC-H₂**.

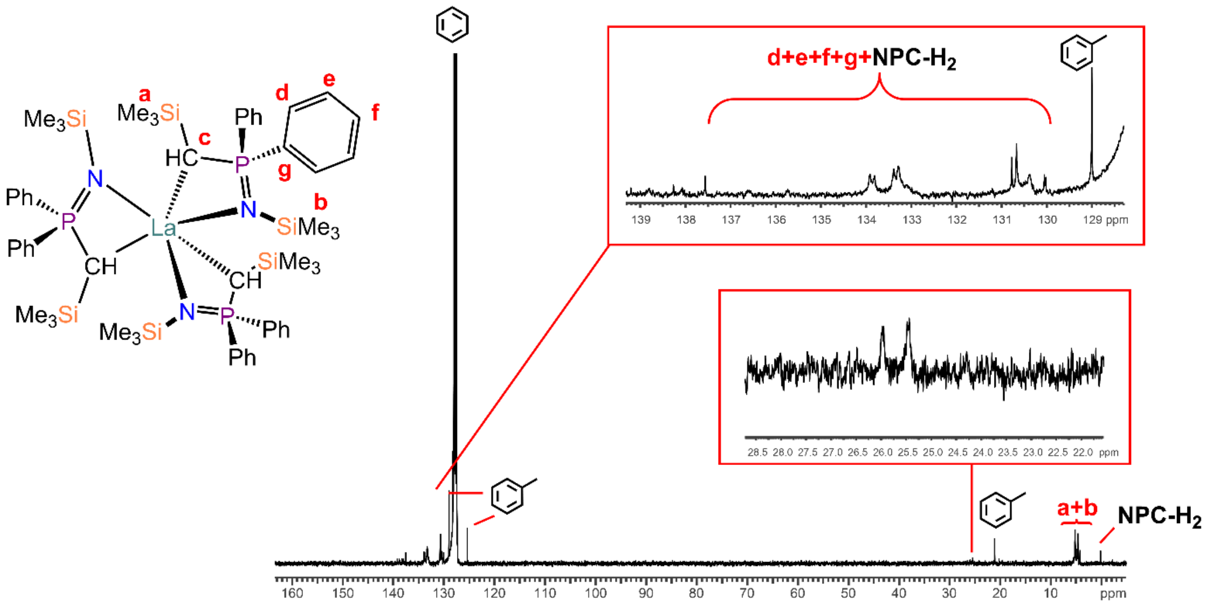

Figure S47: ^{13}C{^{1}H} NMR (101 MHz, 298 K, C_6D_6) spectrum of **2-La**.

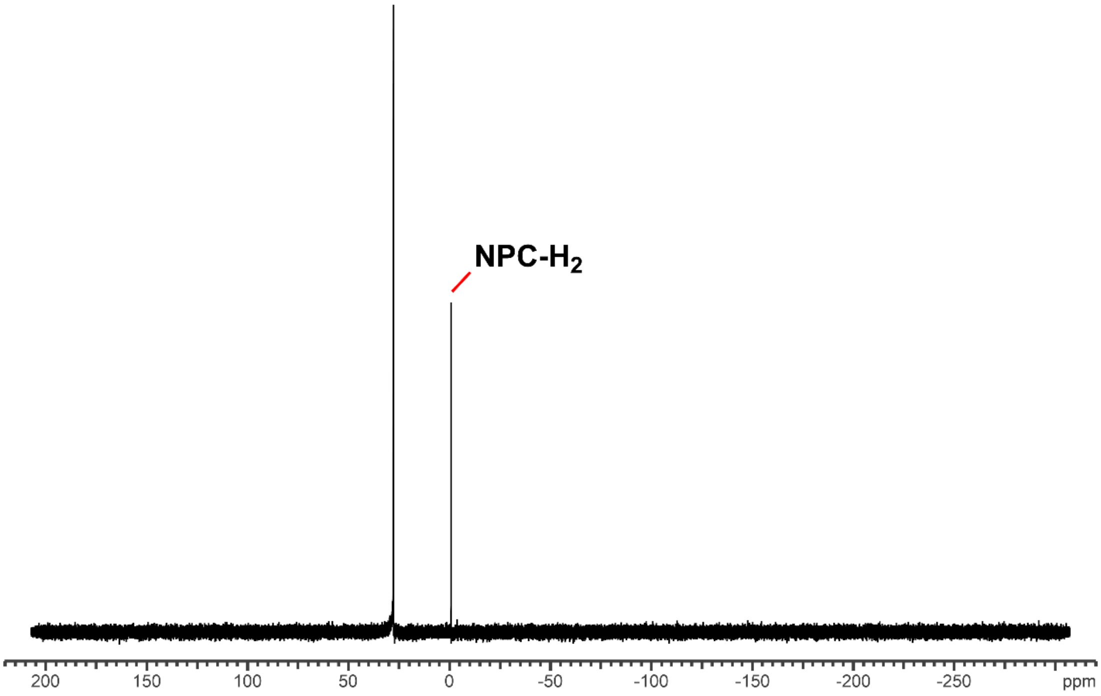

Figure S48: $^{31}P\{^{1}H\}$ NMR (162 MHz, 298 K, C_6D_6) spectrum of **2-La**.

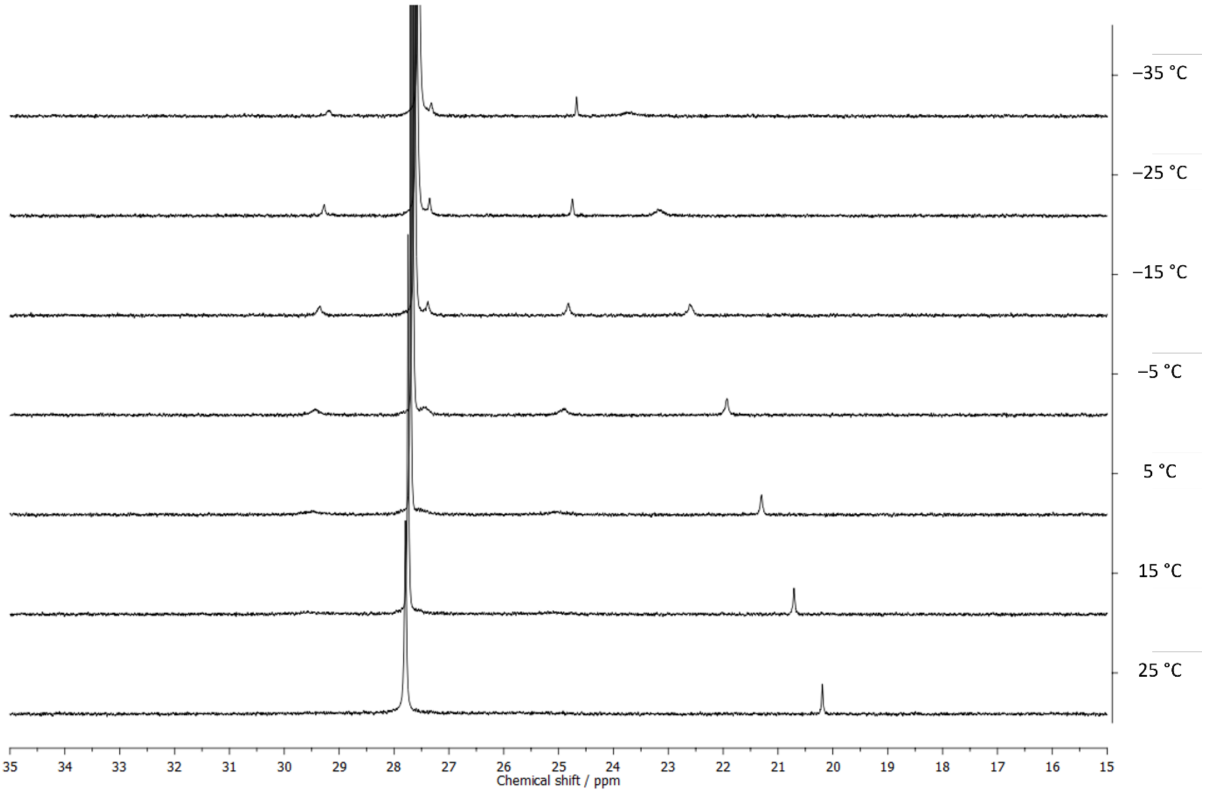

Figure S49: $^{31}P\{^{1}H\}$ VT-NMR study of **2-La** at –35 to 25 °C in d_8-toluene in the region 15.0 – 35.0 ppm.

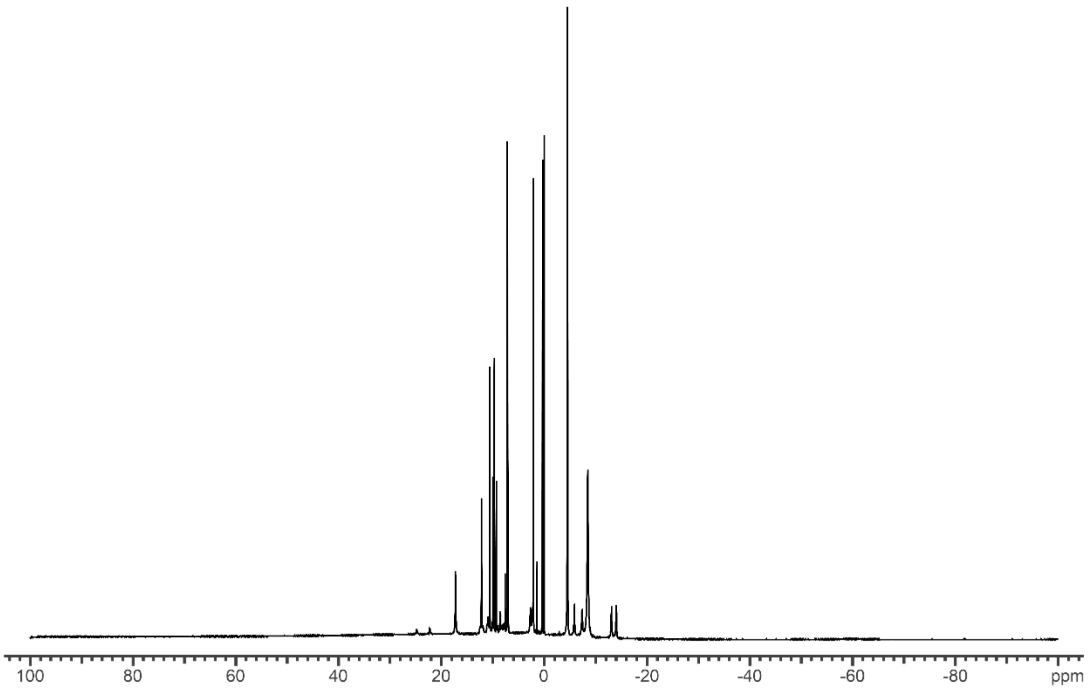

Figure S50: ^{1}H NMR (400 MHz, 298 K, C_6D_6) spectrum of **2-Pr**.

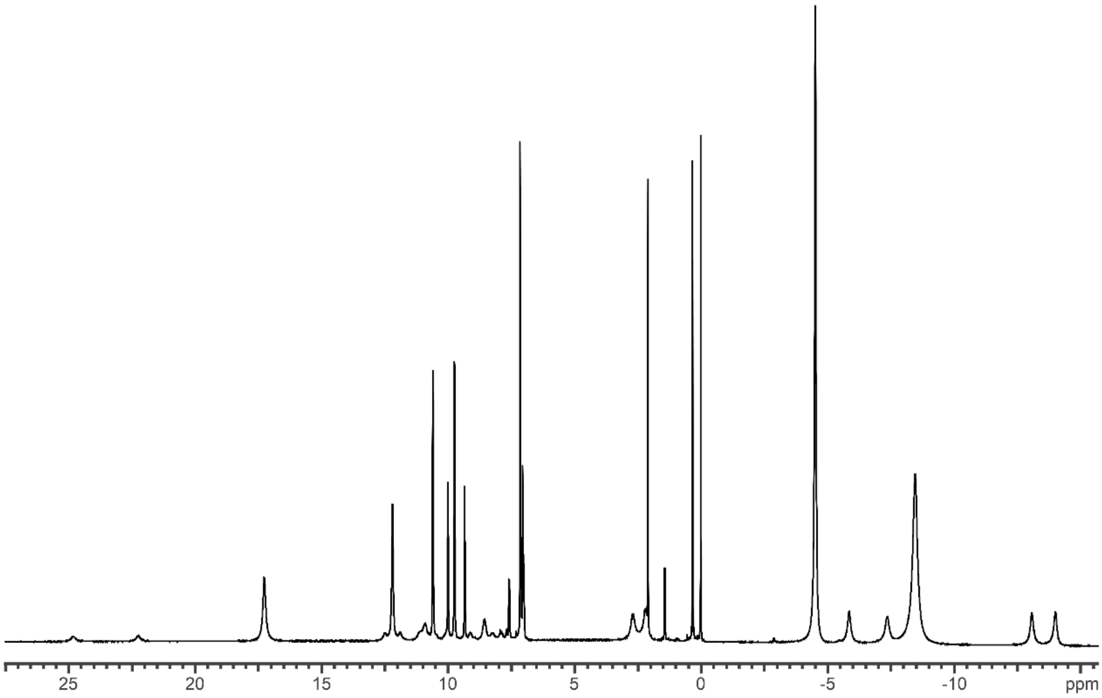

Figure S51: ^{1}H NMR (400 MHz, 298 K, C_6D_6) spectrum of **2-Pr**, zoomed in the region between –15 and 25 ppm.

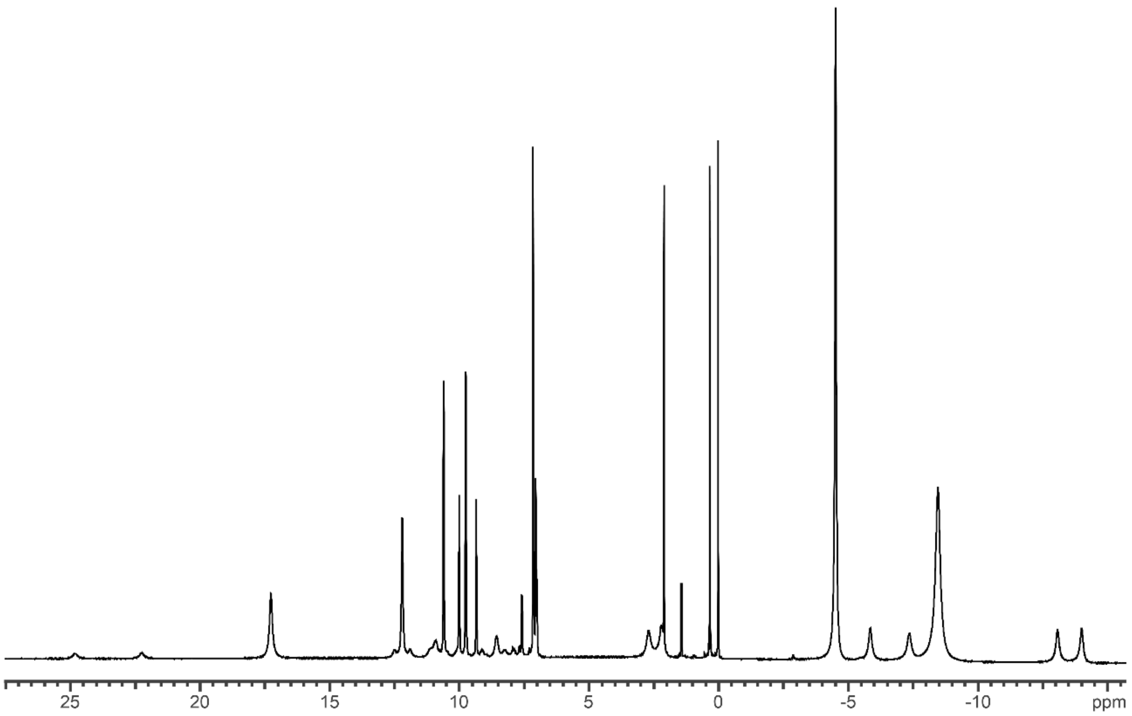

Figure S52: ${}^{31}P\{{}^{1}H\}$ NMR (162 MHz, 298 K, C_6D_6) spectrum of **2-Pr**.

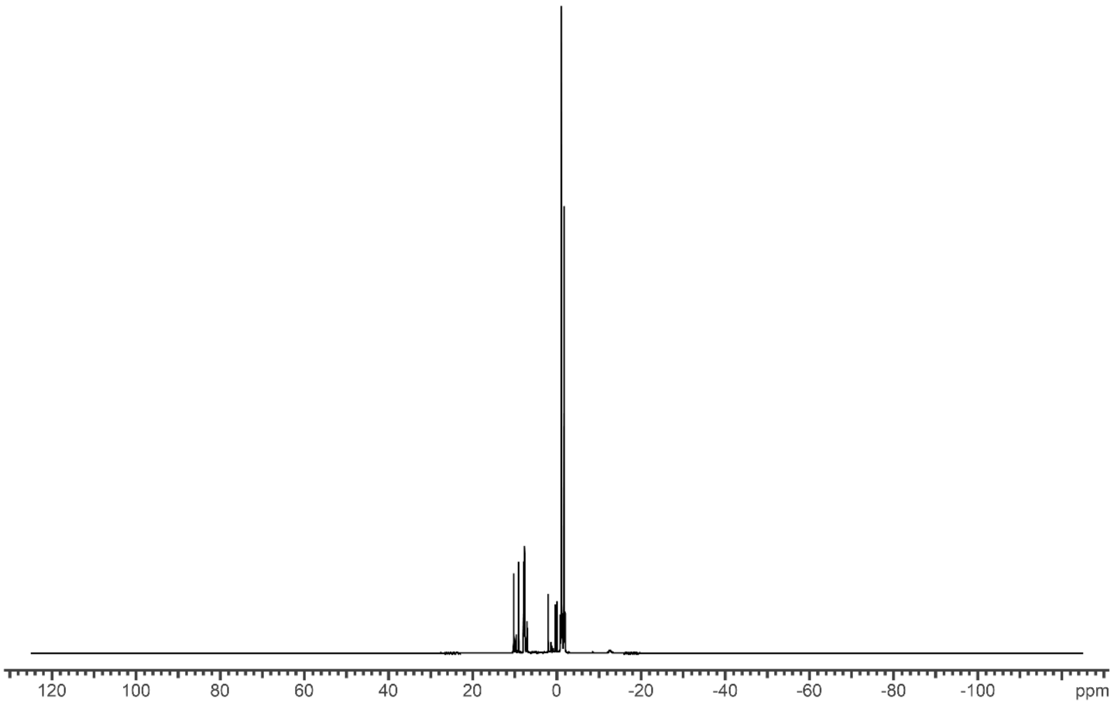

Figure S53: ${}^{1}H$ NMR (400 MHz, 298 K, C_6D_6) spectrum of **2-Sm**.

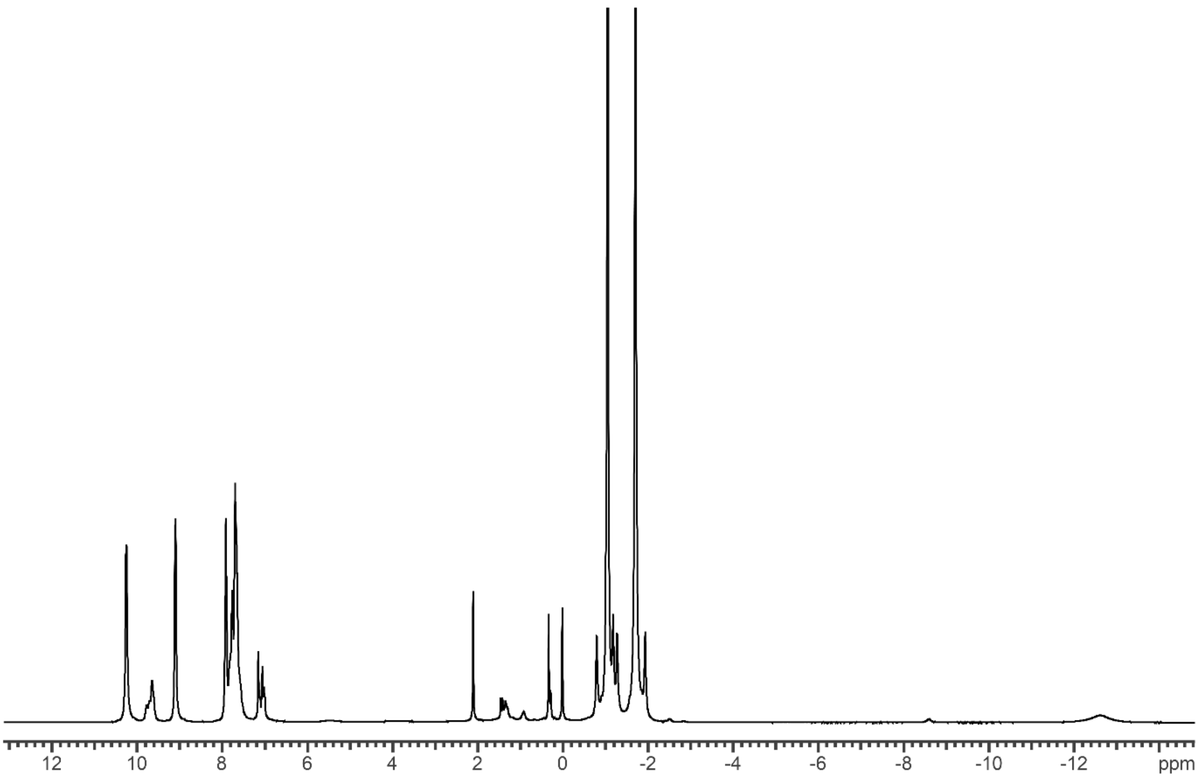

Figure S54: ^{1}H NMR (400 MHz, 298 K, C_6D_6) spectrum of **2-Sm** zoomed in the region between –13 and –12 ppm.

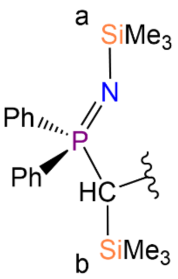

Figure S55: Labelling of the silicon atoms in {NPC-H}$^{-}$.

Table S1: Chemical shifts and $^{2}J_{SiP}$ coupling constants of **1-M·(THF)$_x$** (M = Mg, Ca and Yb, x = 0; M Sr, Ba, x = 2).

Compound	δ_{Si} of Me_3Si-N ($^{2}J_{SiP}$ / Hz)	δ_{Si} of Me_3Si-CH ($^{2}J_{SiP}$ / Hz)
^{TMS}NPC-H_2	*–13.0 (21.3)*	*0.53 (5.9)*
**1-Mg***	–8.2 (5.4)	–7.8 (<0.7 Hz)
**1-Ca**	–9.7 (9.3)	–11.8 (1.8)
**1-Sr·(THF)$_2$**	–10.7 (10.1)	–13.0 (3.5)
**1-Ba·(THF)$_2$**	–10.3 (11.5)	–13.4 (5.5)
**1-Yb**	–10.4 (9.6)	–12.5 (2.3)

*Recorded at 105°C

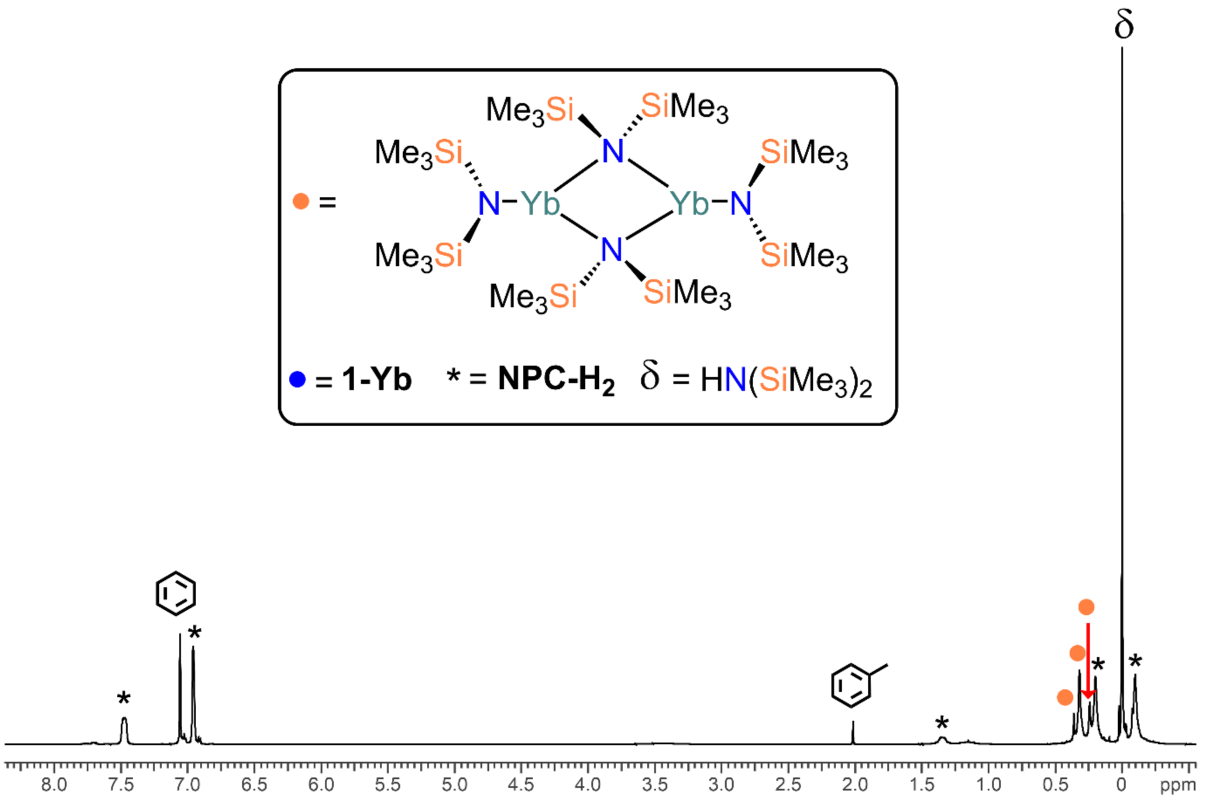

Figure S56: ^{1}H NMR (500 MHz, 298 K, C_6D_6) spectra of the reactivity study between **1-Yb** and $HN(SiMe_3)_2$ on an NMR scale.

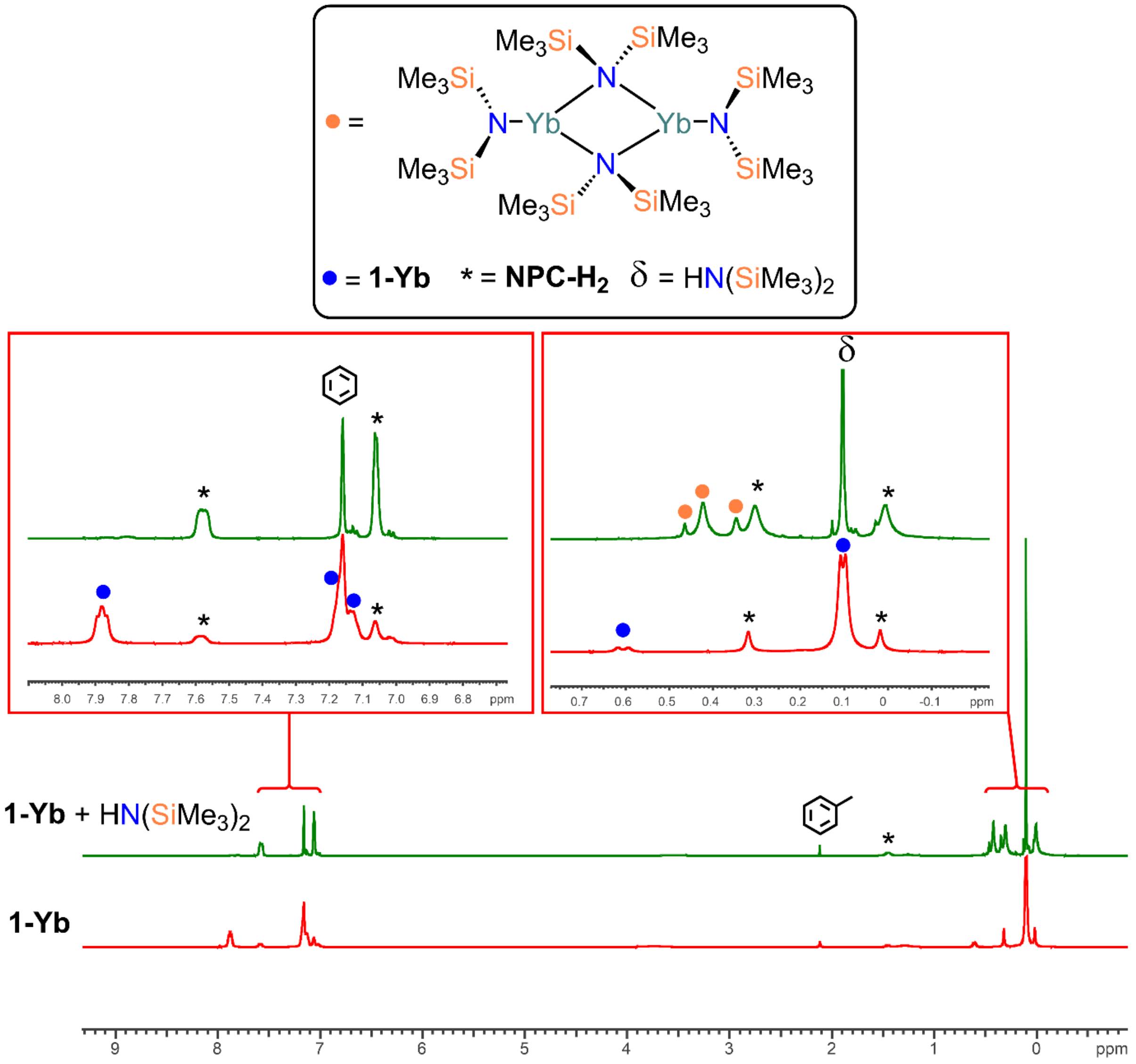

Figure S57: ^{1}H NMR (500 MHz, 298 K, C_6D_6) spectra of the reactivity study between **1-Yb** and $HN(SiMe_3)_2$ on an NMR scale. Bottom spectrum (red): **1-Yb**; top spectrum (green): **1-Yb** reacted with $HN(SiMe_3)_2$ (r.t., 24 h).

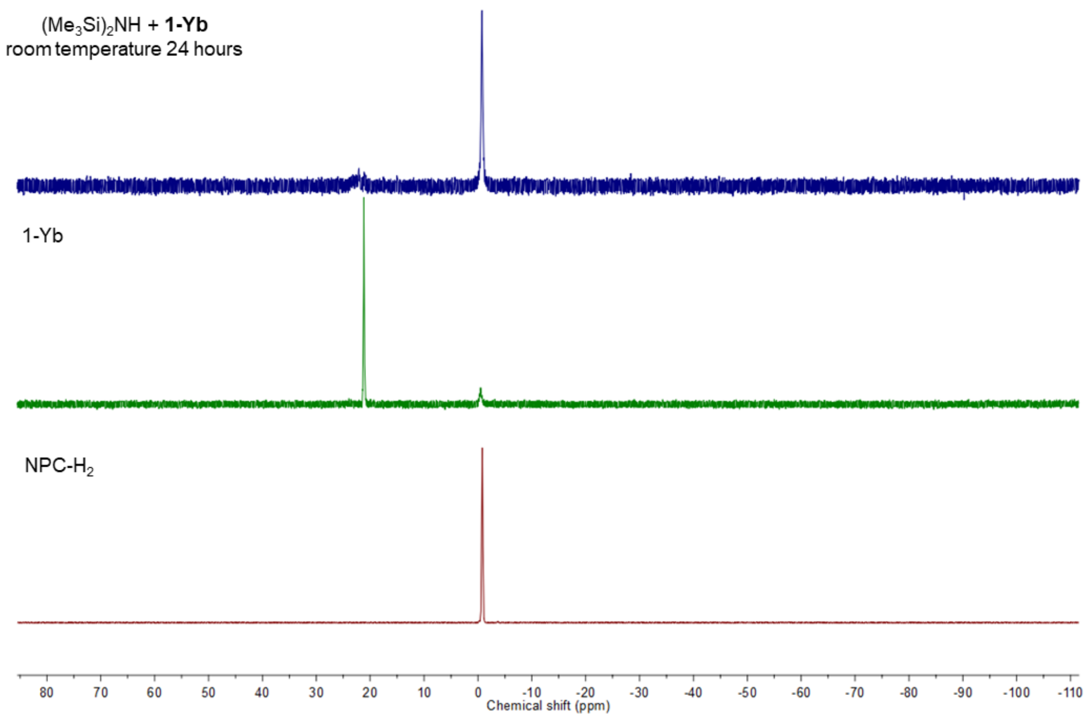

Figure S58: $^{31}P\{^{1}H\}$ NMR (243 MHz, 298 K, C_6D_6) spectra of the reactivity study between **1-Yb** and $HN(SiMe_3)_2$ on an NMR scale.

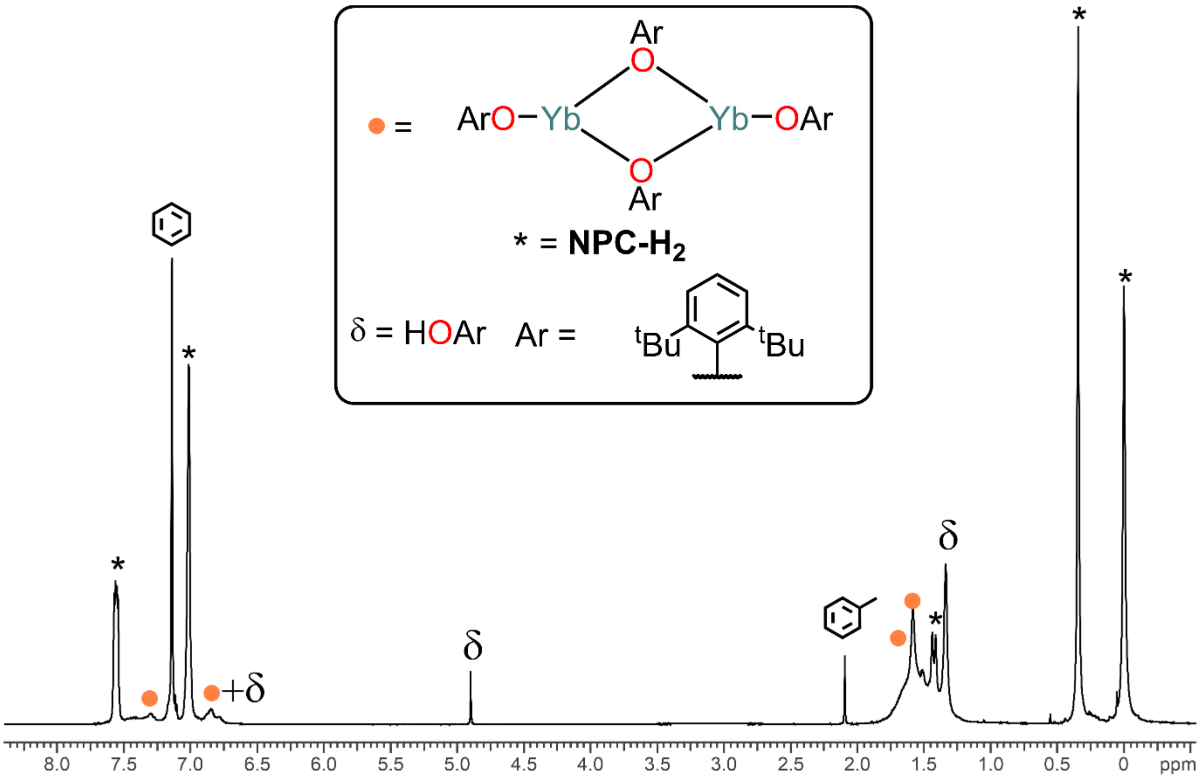

Figure S59: ^{1}H NMR (500 MHz, 298 K, C_6D_6) spectrum of the reactivity study between **1-Yb** and 2,6-di-*tert*-butylphenol on an NMR scale.

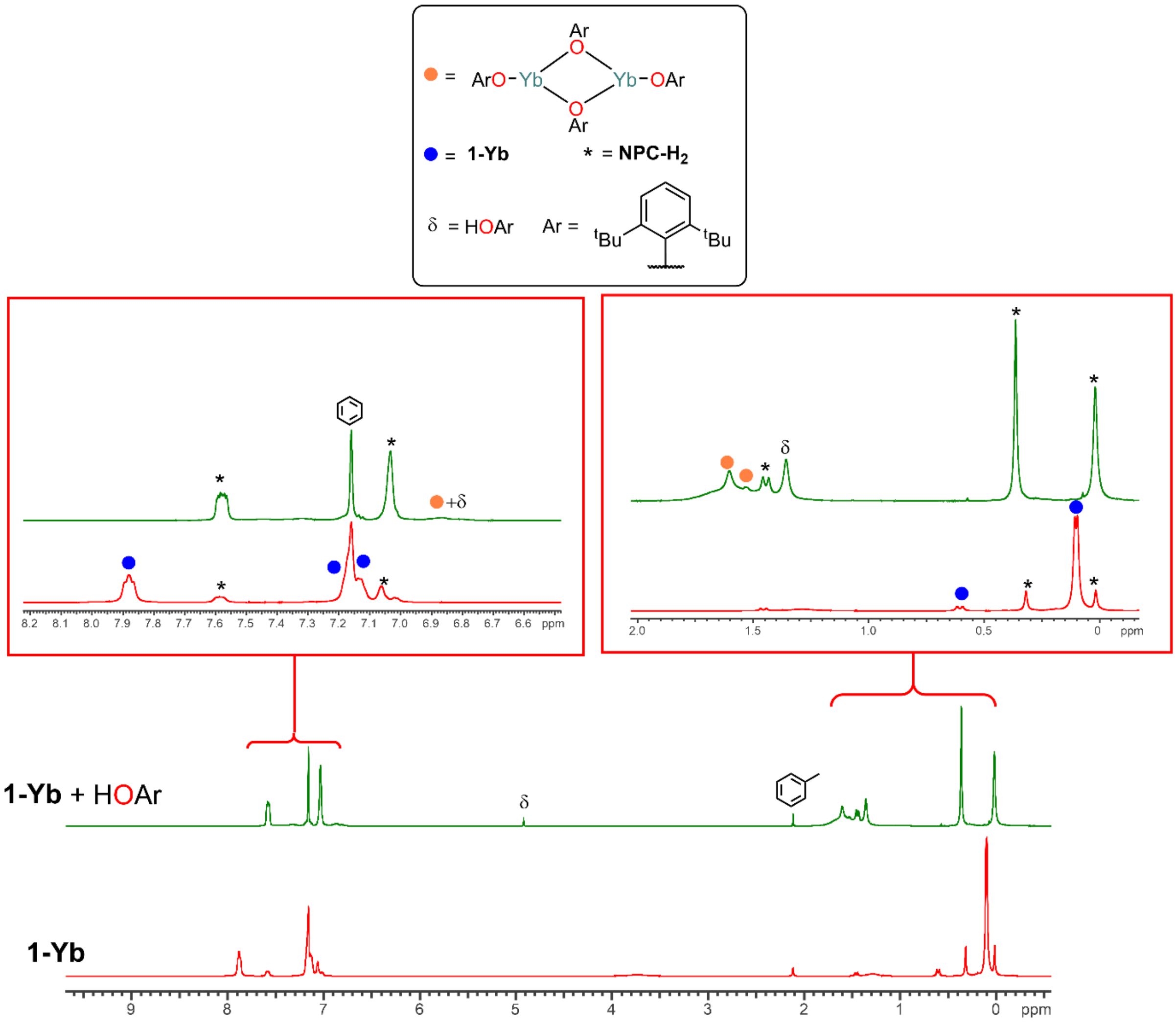

Figure S60: ^{1}H NMR (500 MHz, 298 K, C_6D_6) spectra of the reactivity study between **1-Yb** and 2,6-di-*tert*-butylphenol on an NMR scale. Bottom spectrum (red): **1-Yb**; top spectrum (green): **1-Yb** reacted with 2,6-di-*tert*-butylphenol (r.t., 24 h).

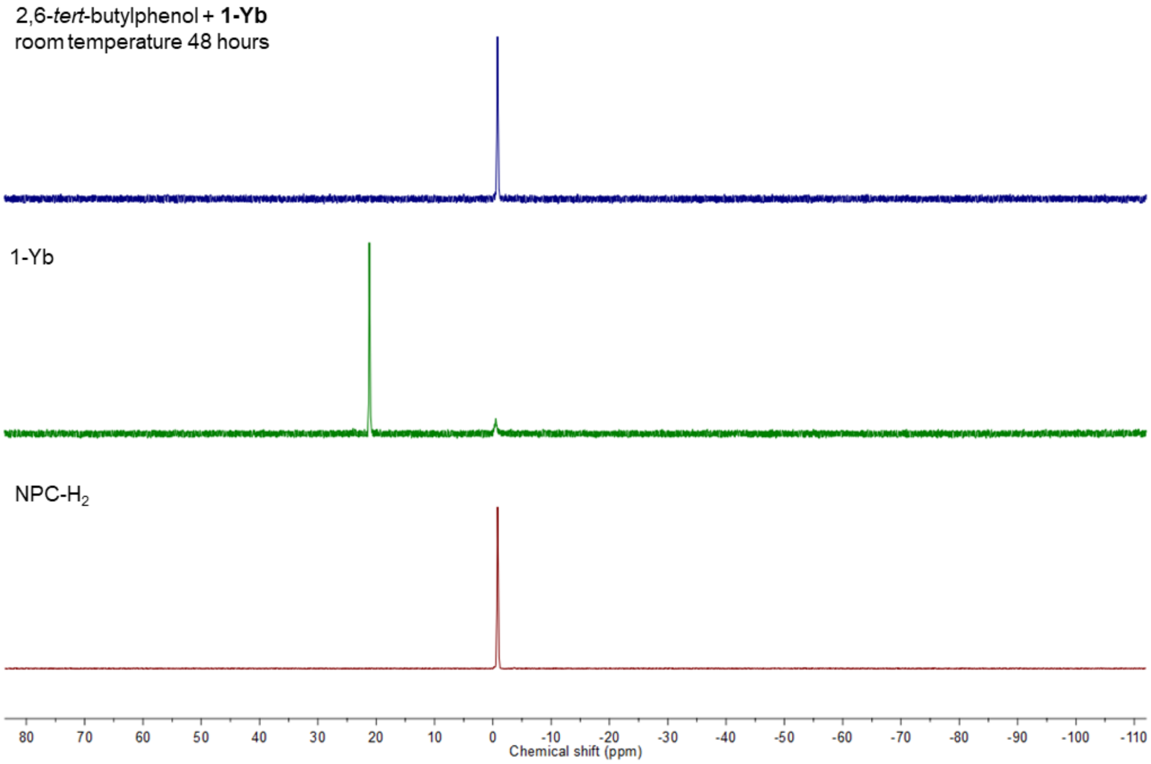

Figure S61: $^{31}P\{^{1}H\}$ NMR (243 MHz, 298 K, C_6D_6) spectrum of the reactivity study between **1-Yb** and 2,6-di-*tert*-butylphenol on an NMR scale.

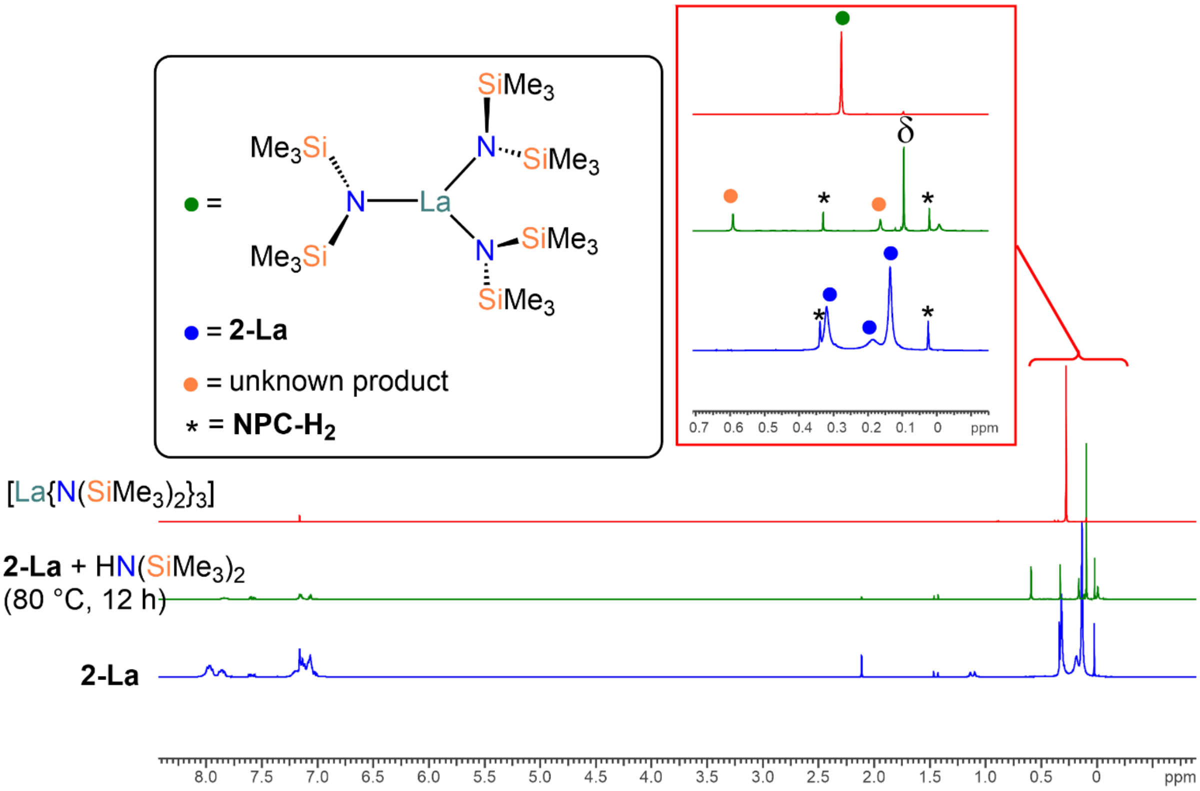

Figure S62: ^{1}H NMR (400 MHz, 298 K, C_6D_6) spectra of the reactivity study between **2-La** and $HN(SiMe_3)_2$ on an NMR scale. Top spectrum (red): $[La\{N(SiMe_3)_2\}_3]$; middle spectrum (green): reaction of **2-La** $HN(SiMe_3)_2$ (80 ºC, 12 h); bottom spectrum (blue): **2-La**.

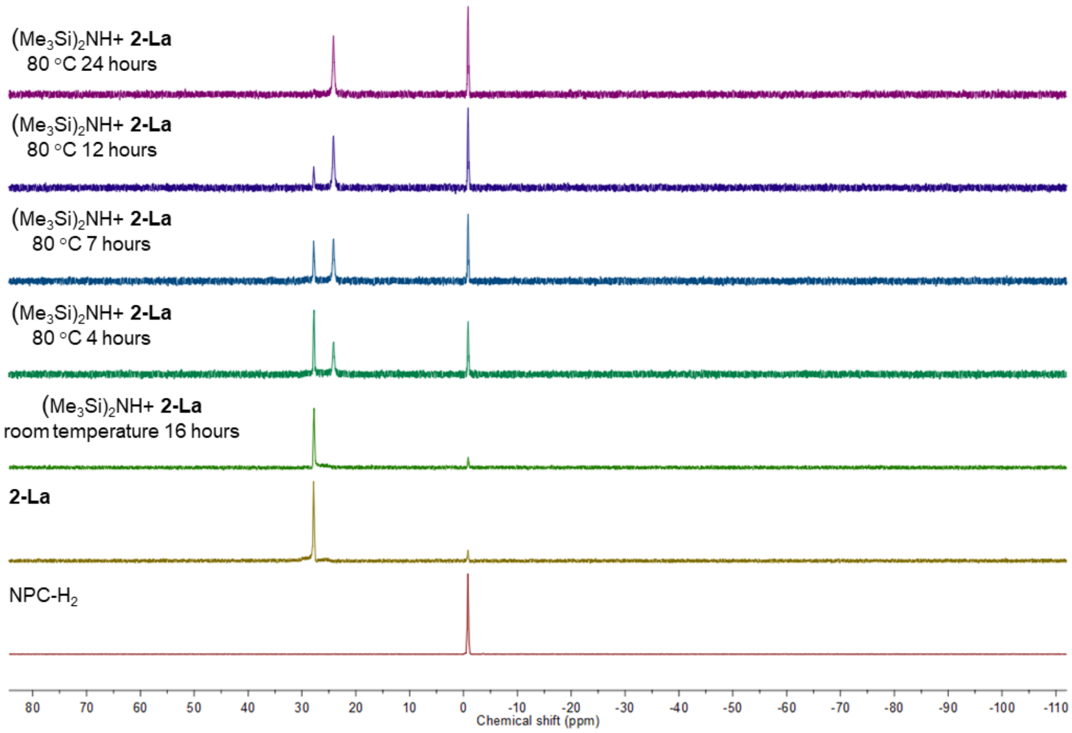

Figure S63: $^{31}P\{^{1}H\}$ NMR (162 MHz, 298 K, C_6D_6) spectrum of the reactivity study between **2-La** and $HN(SiMe_3)_2$ on an NMR scale.

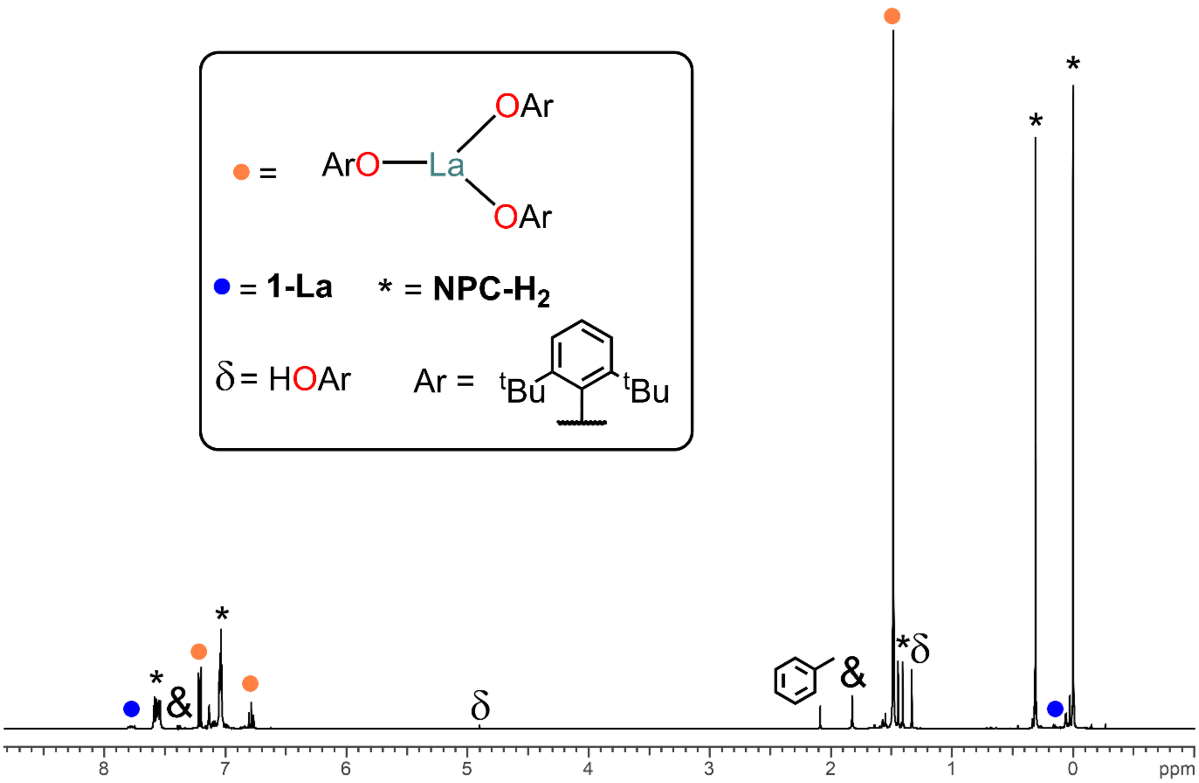

Figure S64: ^{1}H NMR (400 MHz, 298 K, C_6D_6) spectrum of the reactivity study between **2-La** and 2,6-di-*tert*-butylphenol on an NMR scale. '&' denotes unknown by-products.

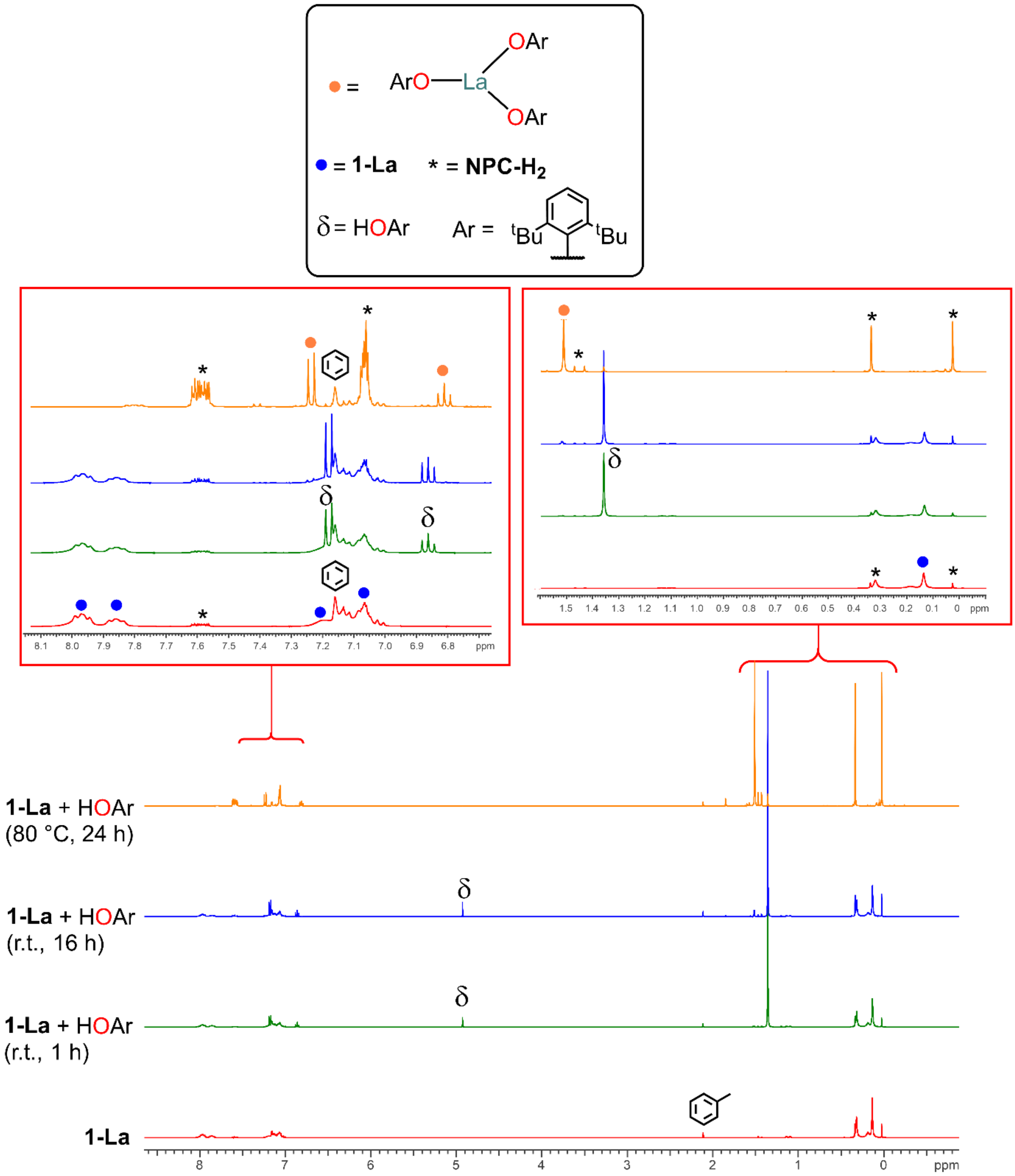

Figure S65: ^{1}H NMR (400 MHz, 298 K, C_6D_6) spectra of the reactivity study between **2-La** and 2,6-di-*tert*-butylphenol on an NMR scale.

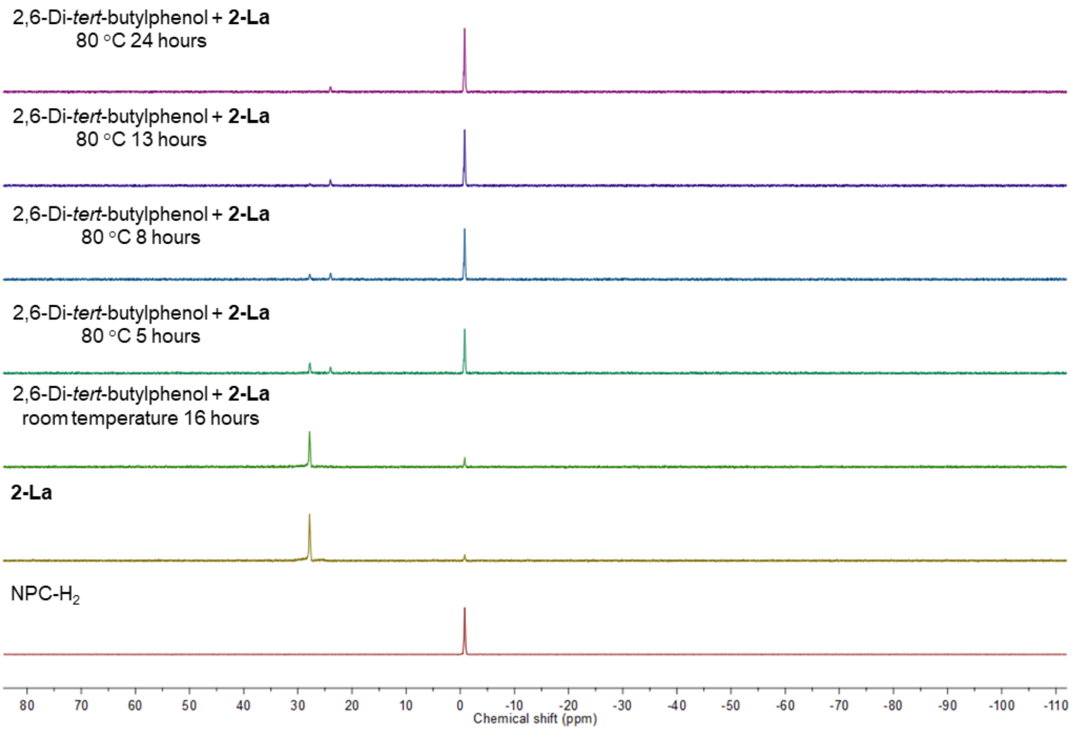

Figure S66: $^{31}P\{^{1}H\}$ NMR (162 MHz, 298 K, C_6D_6) spectra of the reactivity study between **2-La** and 2,6-di-*tert*-butylphenol on an NMR scale.

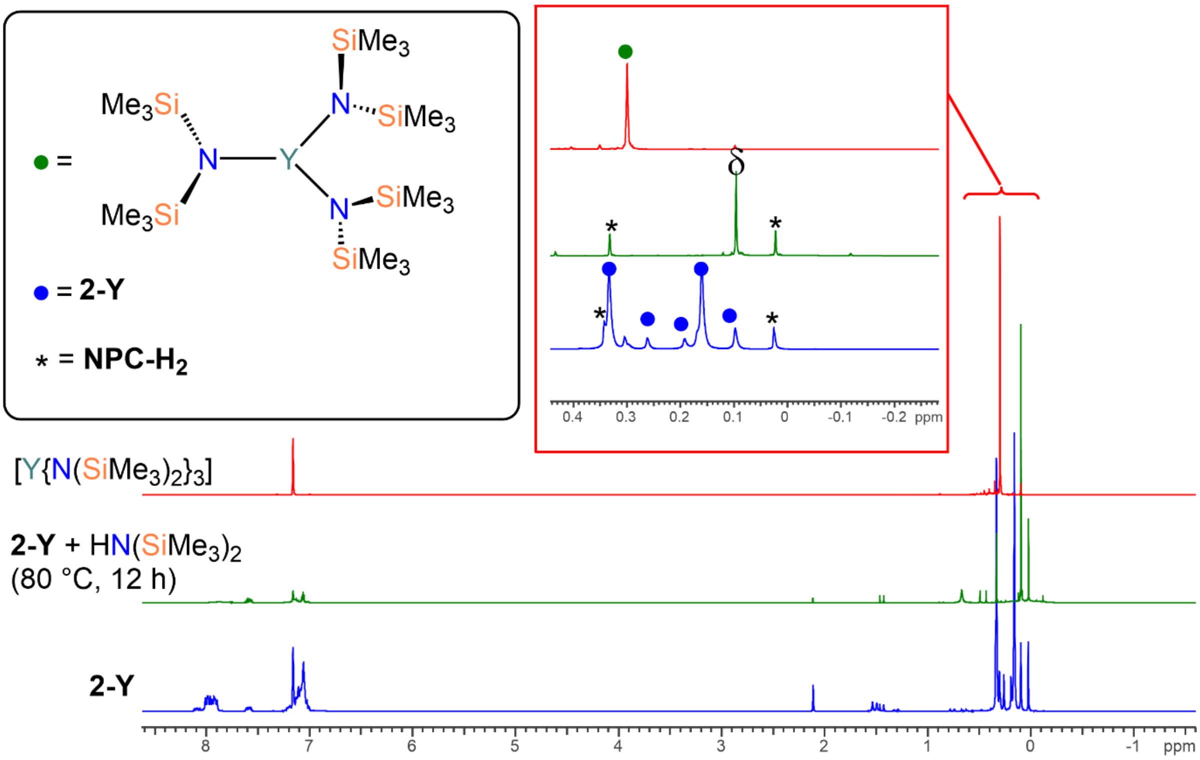

Figure S67: ^{1}H NMR (400 MHz, 298 K, C_6D_6) spectrum of the reactivity study between **2-Y** and $HN(SiMe_3)_2$.

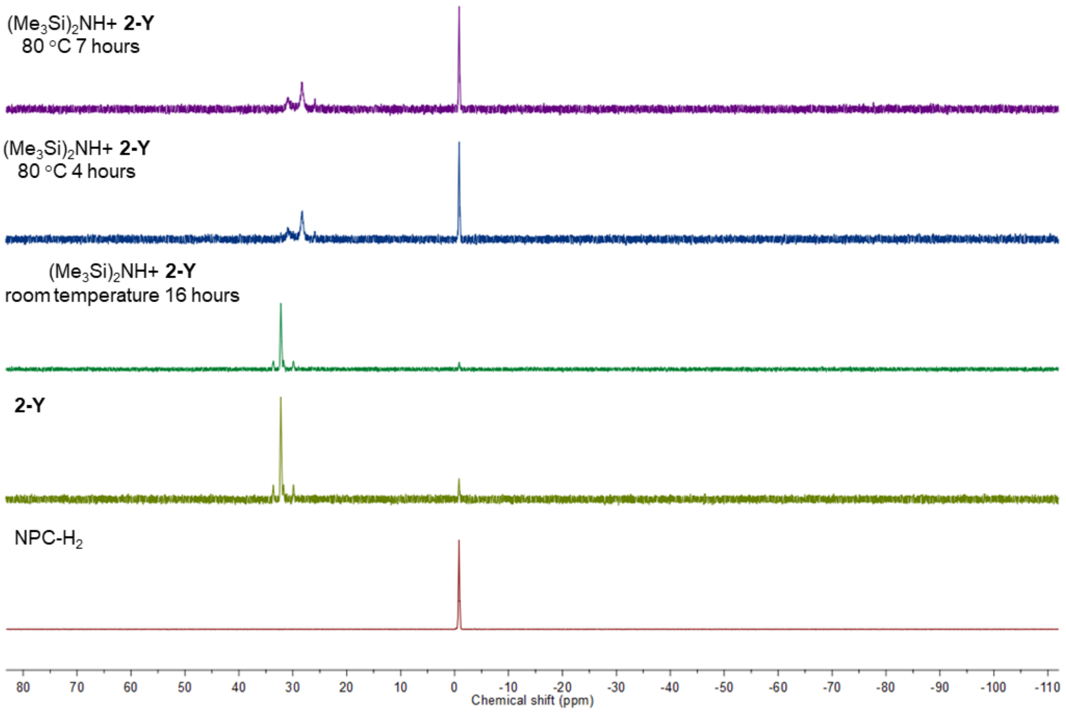

Figure S68: $^{31}P\{^{1}H\}$ NMR (162 MHz, 298 K, C_6D_6) spectrum of the reactivity study between **2-Y** and $HN(SiMe_3)_2$ on an NMR scale in the region –110 to 80 ppm.

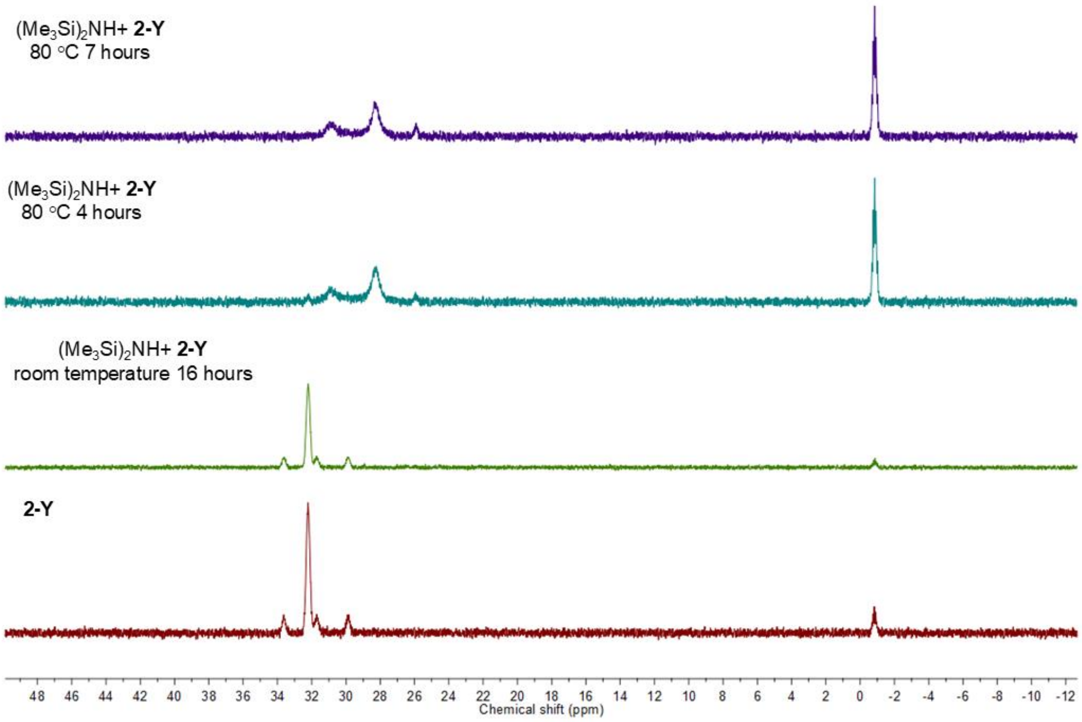

Figure S69: $^{31}P\{^{1}H\}$ NMR (162 MHz, 298 K, C_6D_6) spectrum of the reactivity study between **2-Y** and $HN(SiMe_3)_2$ on an NMR scale in the region –12 to 50 ppm.

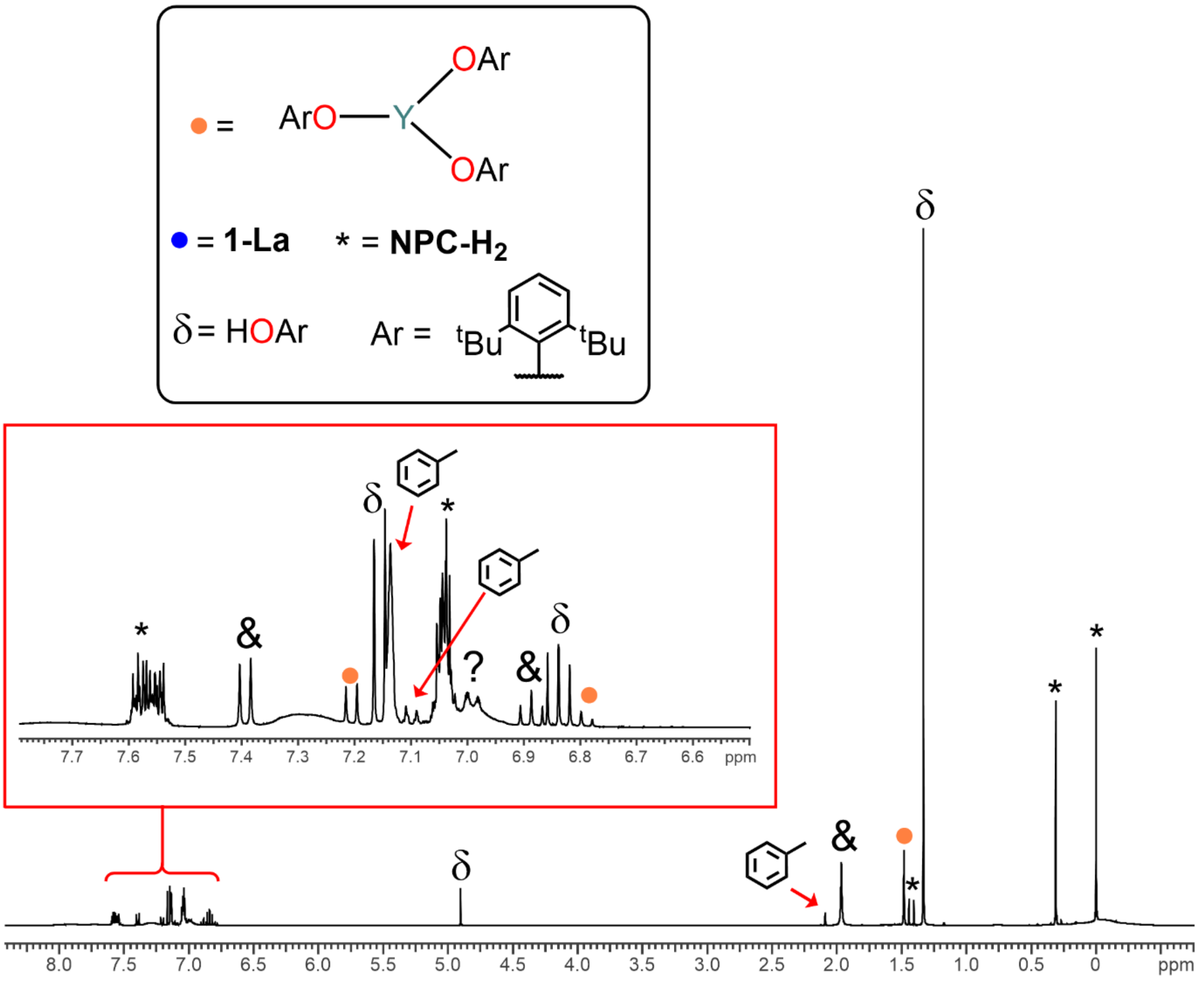

Figure S70: ^{1}H NMR (400 MHz, 298 K, C_6D_6) spectra of the reactivity study between **2-Y** and 2,6-di-*tert*-butylphenol on an NMR scale. '&' denotes an unknown by-product; '?' denotes and unknown impurity present in the starting material **2-Y**.

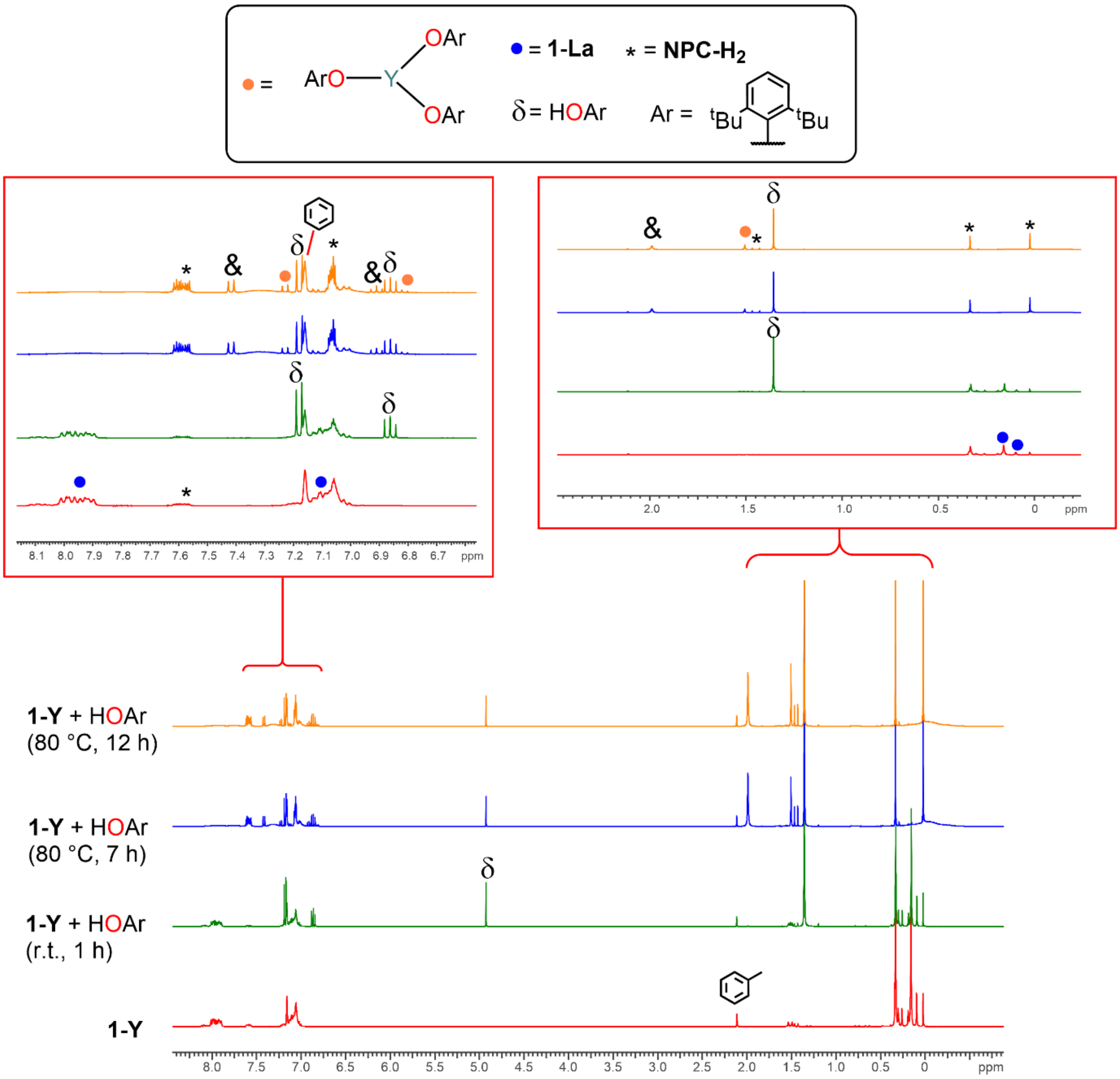

Figure S71: ^{1}H NMR (400 MHz, 298 K, C_6D_6) spectra of the reactivity study between **2-Y** and 2,6-di-*tert*-butylphenol on an NMR scale. '&' denotes an unknown by-product; '?' denotes and unknown impurity present in the starting material **2-Y**.

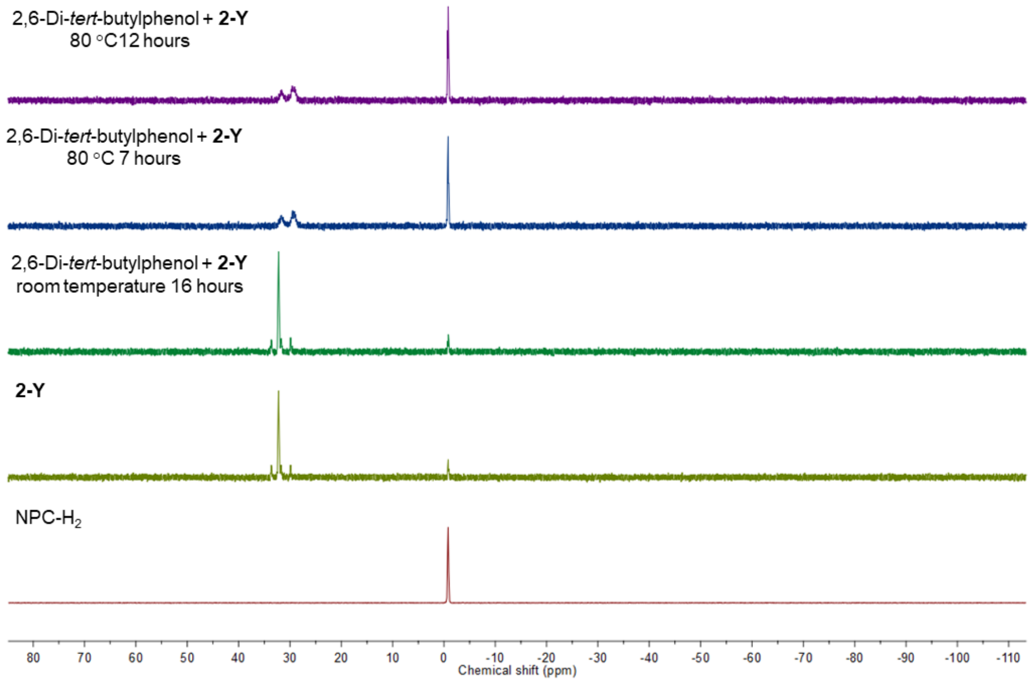

Figure S72: $^{31}P\{^{1}H\}$ NMR (162 MHz, 298 K, C_6D_6) spectrum of the reactivity study between **2-Y** and 2,6-di-*tert*-butylphenol on an NMR scale.

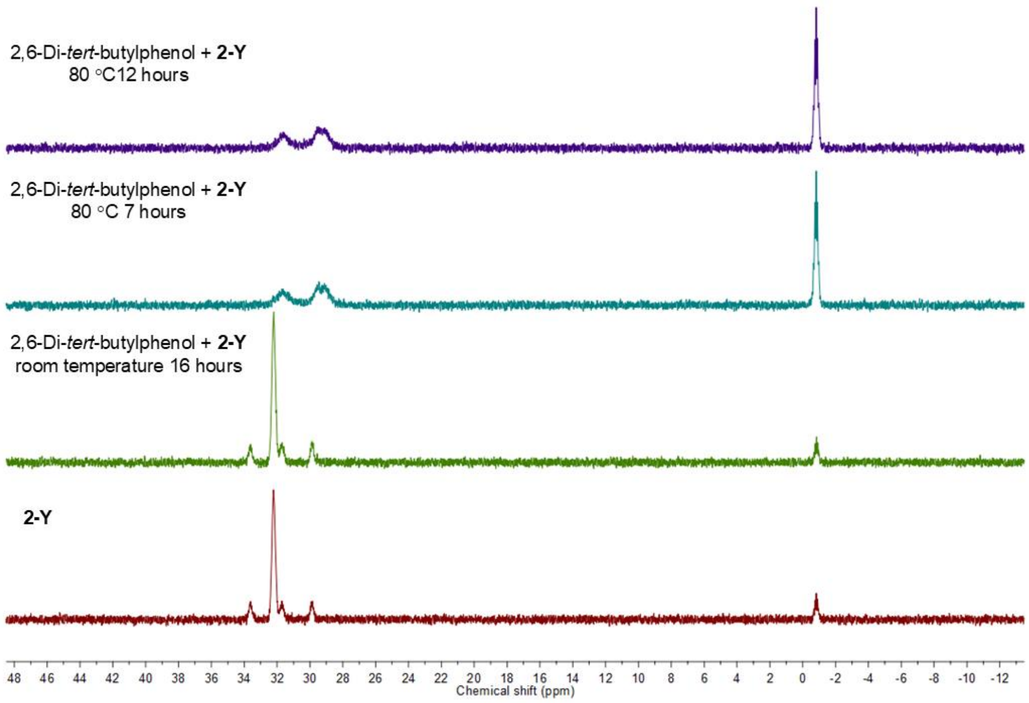

Figure S73: $^{31}P\{^{1}H\}$ NMR (162 MHz, 298 K, C_6D_6) spectrum of the reactivity study between **2-Y** and 2,6-di-*tert*-butylphenol on an NMR scale in the region –12 to 48 ppm.

S2. IR data

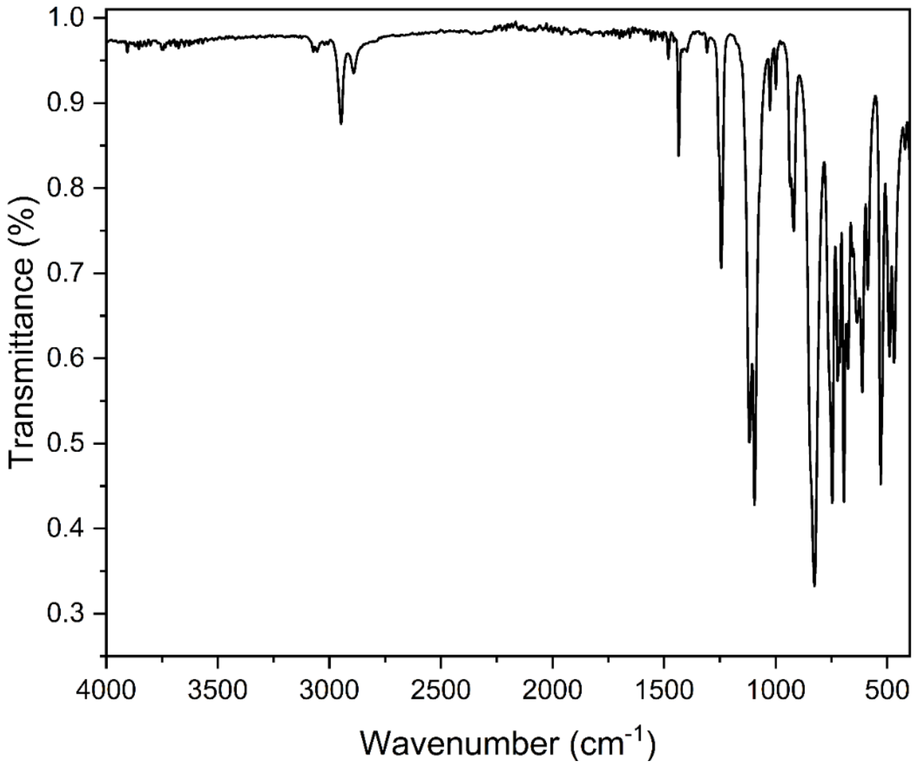

Figure S74: IR spectrum of **1-Mg**.

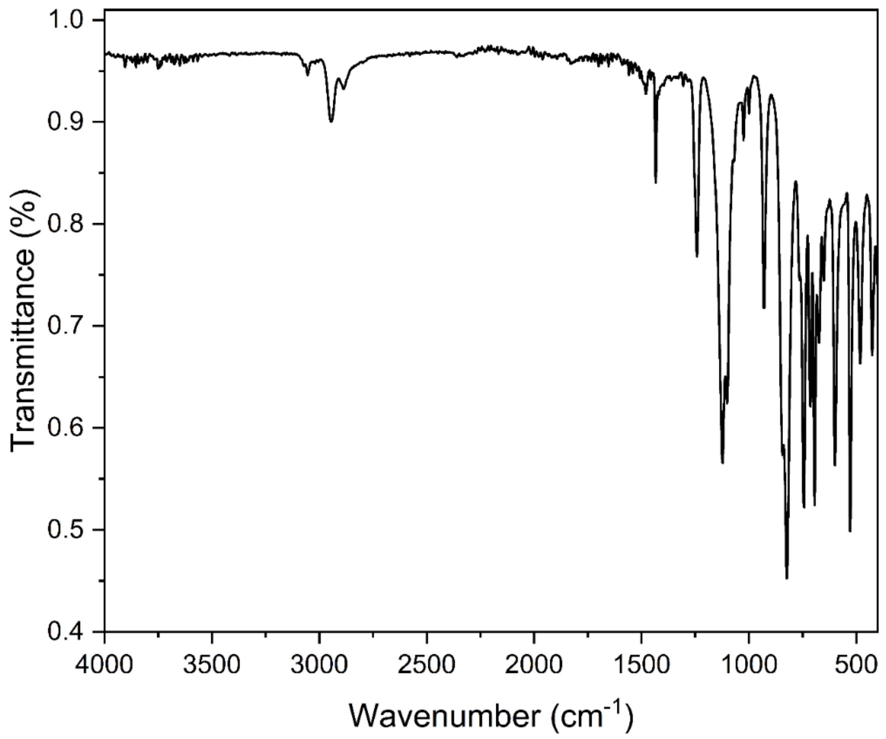

Figure S75: IR spectrum of **1-Ca**.

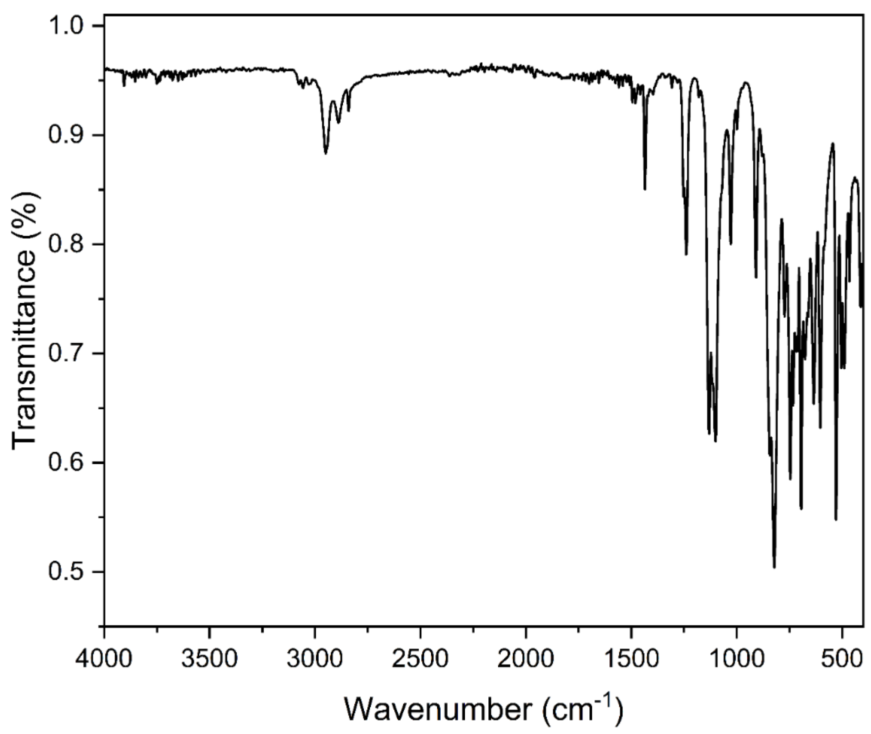

Figure S76: IR spectrum of **1-Ca·(THF)**.

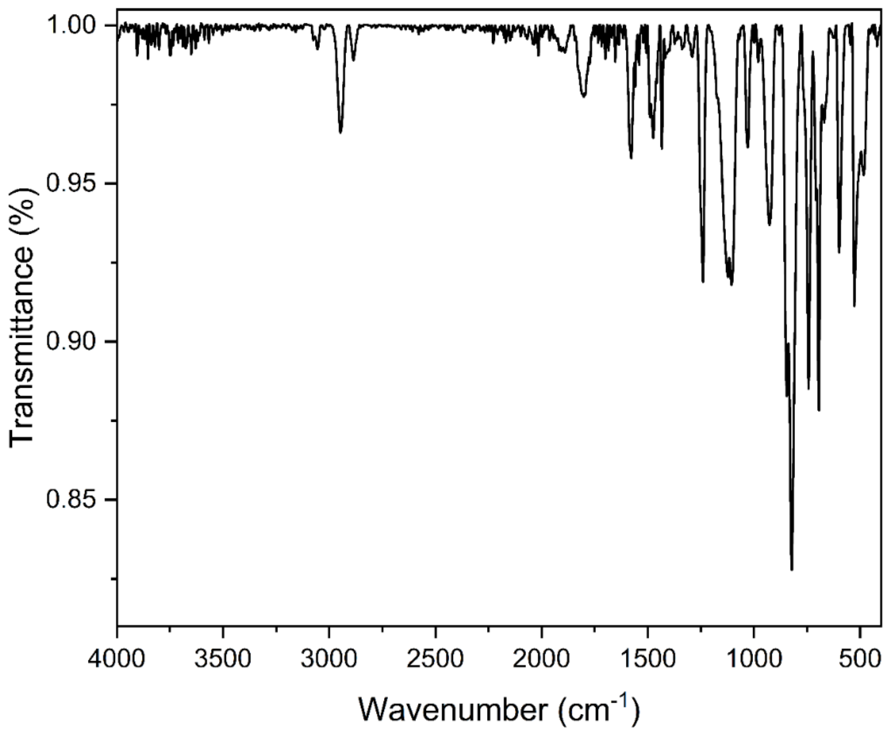

Figure S77: IR spectrum of **1-Sr·(THF)$_2$**.

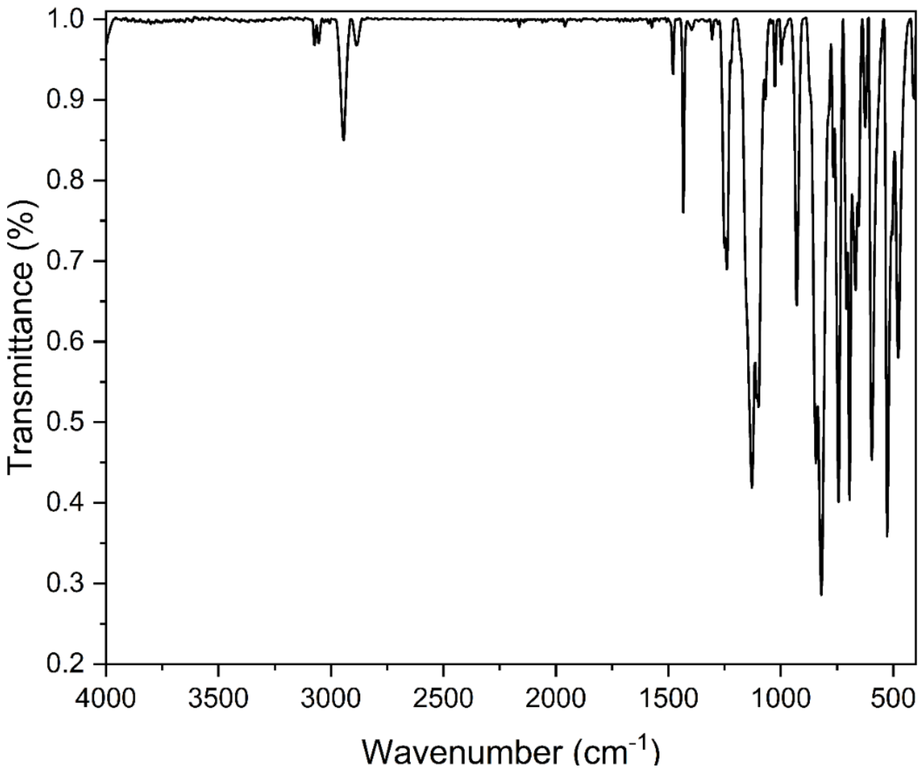

Figure S78: IR spectrum of **1-Ba·(THF)₂**.

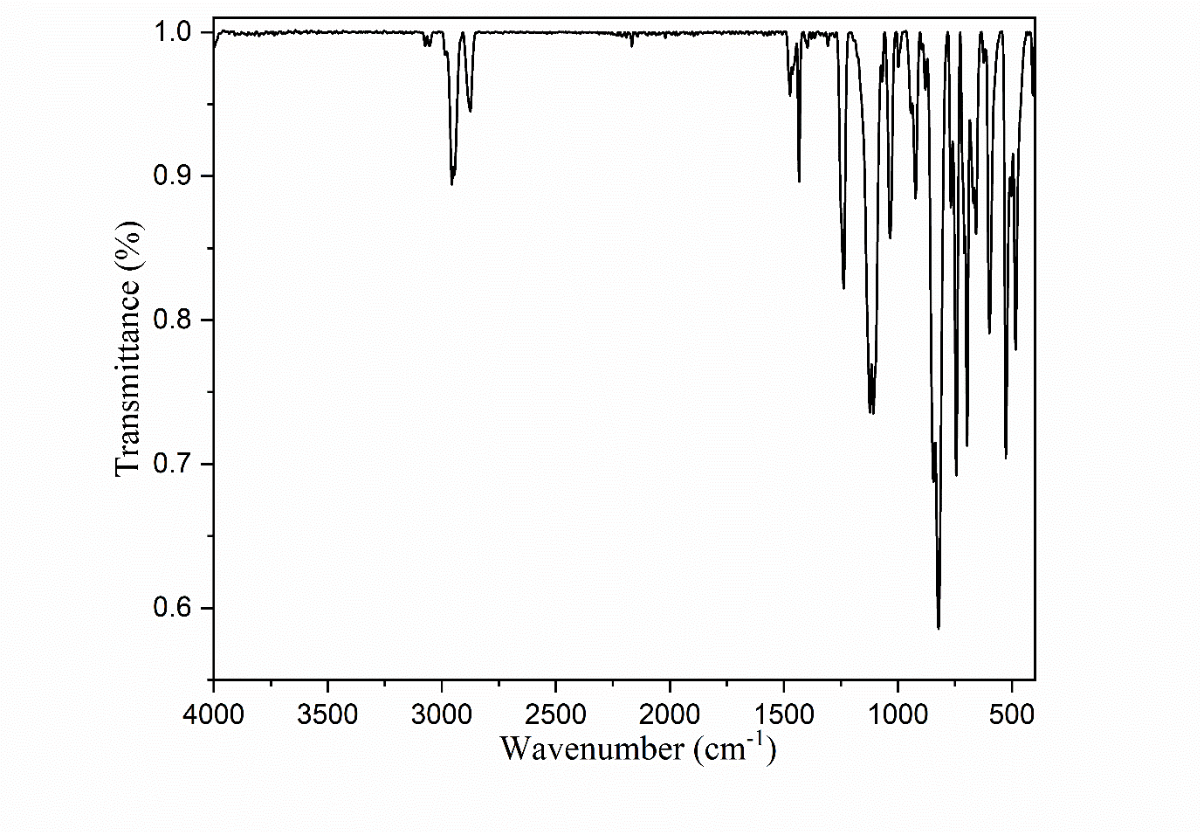

Figure S79: IR spectrum of **1-Sm·(THF)₂**.

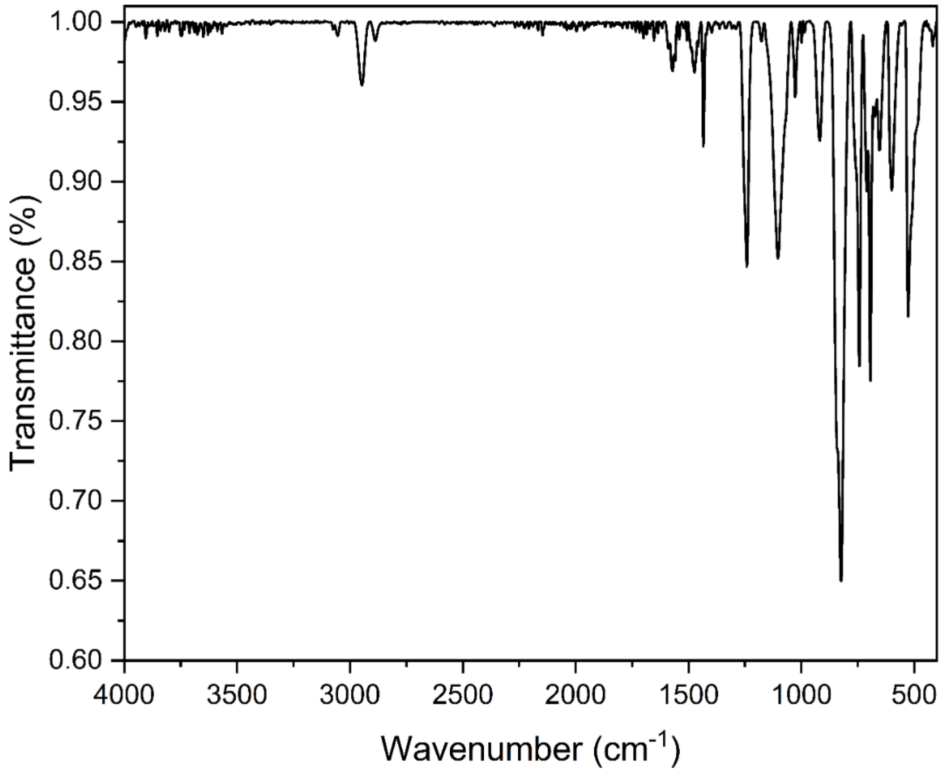

Figure S80: IR spectrum of **2-Sm**.

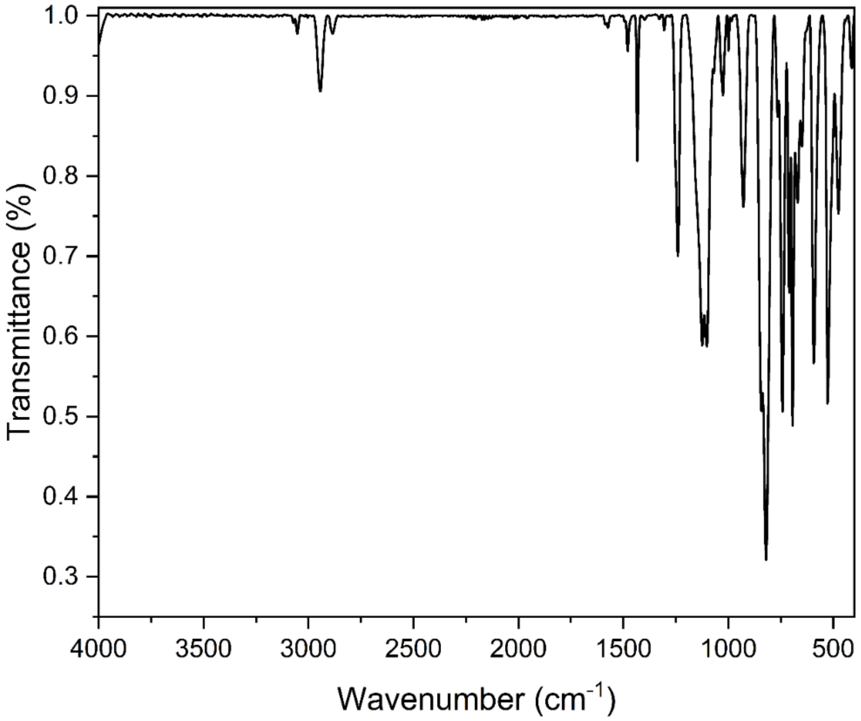

Figure S81: IR spectrum of **1-Eu**.

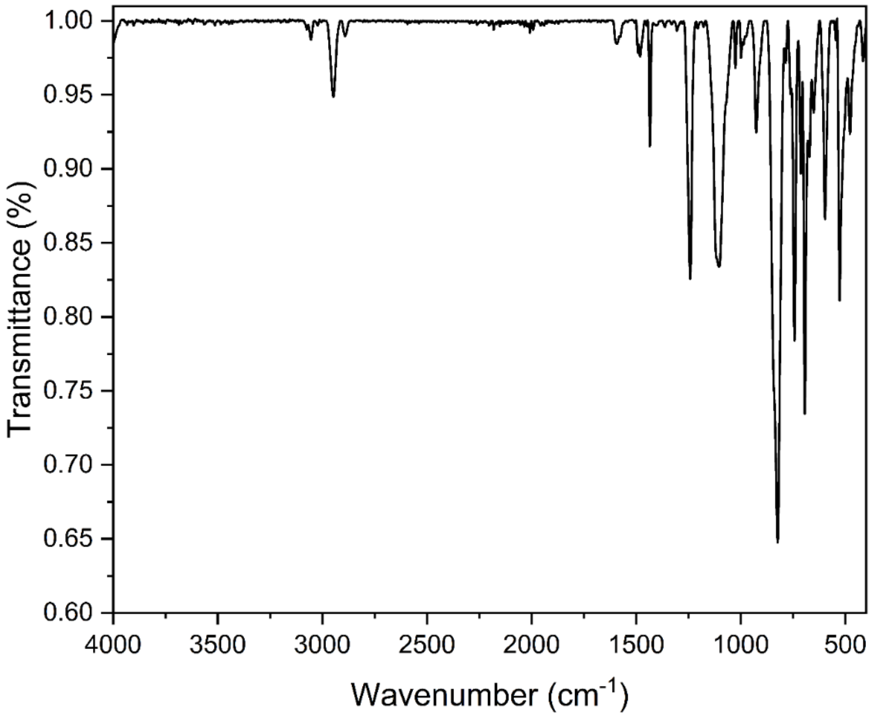

Figure S82: IR spectrum of **1-Yb**.

S3. UV-vis data

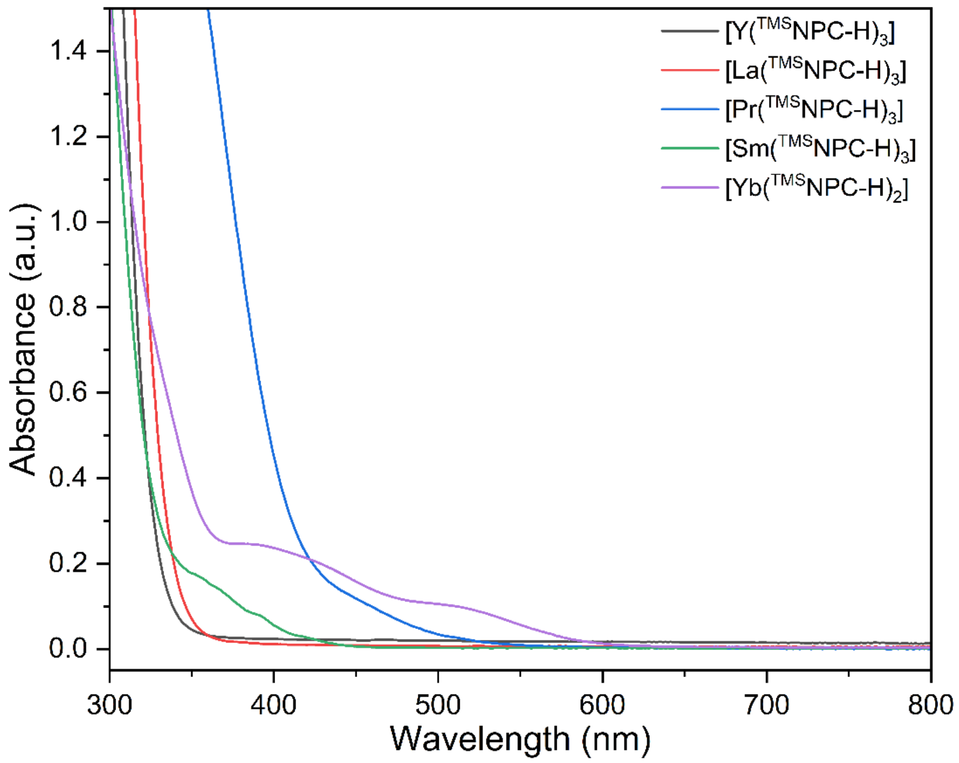

Figure S83: UV-vis spectra (THF, 0.2 M) of **1-Yb** and **2-RE** (RE = Y, La, Pr, Sm).

S4. Crystallography

Crystallographic method

The crystal data for all compounds are compiled in Tables S1–S3. Crystals were examined using a Bruker D8 Quest diffractometer with a Photon III detector and a microfocus source with Cu–Kα radiation (λ = 1.54178). Intensities were integrated from data recorded on 1.0° frames by ω rotation. A multiscan method absorption correction with a beam profile was applied.[1,2] The structures were solved using SHELXS or SHELXT;[3] the datasets were refined by full-matrix least-squares on reflections with $F^2 \geq 2\sigma(F^2)$ values, with anisotropic displacement parameters for all non-hydrogen atoms, and with constrained riding hydrogen geometries;[4] U_{iso}(H) was set at 1.2 (1.5 for methyl groups) times U_{eq} of the parent atom. The largest features in final difference syntheses were close to heavy atoms and were of no chemical significance. SHELX[3,4] was employed through OLEX2 for structure solution and refinement.[5] The structures have been deposited with the Cambridge Crystallographic Data Centre (CCDC 2408361-2408372, 2408465 and 2431369). This information can be obtained free of charge from www.ccdc.cam.ac.uk/data_request/cif.

Table S2: Crystallographic data for **1-Ca**, **1-Ca·(THF)**, and **1-Yb**

	**1-Ca**	**1-Ca·(THF)**	**1-Yb**
Formula	$C_{38}H_{58}CaN_2P_2Si_4$	$C_{45.5}H_{70}CaN_2OP_2Si_4$	$C_{38}H_{58}N_2P_2Si_4Yb$
Formula Weight	757.24	875.41	890.20
Crystal Size, mm	0.254×0.11×0.55	0.335×0.264×0.238	0.086×0.072×0.062
Crystal System	Triclinic	Triclinic	Triclinic
Space group	*P*-1	*P*-1	*P*-1
a, Å	9.2742(7)	11.9165(4)	9.3197(4)
b, Å	13.3547(9)	13.1578(4)	13.3149(5)
c, Å	18.7233(13)	17.6229(6)	18.7148(8)
α, °	78.526(4)	76.0980(10)	78.608(2)
β, °	76.568(5)	89.6590(10)	76.539(2)
γ, °	80.845(4)	73.8290(10)	80.782(2)
V, Å^3	2194.9(3)	2570.61(15)	2198.1(2)
Z	2	2	2
Temperature, K	120	120	120
ρ_{calc}, g cm^{-3}	1.146	1.131	1.345
μ, mm^{-1}	3.166	2.780	5.863
F(*000*)	812	942	912
No. of reflections (unique)	20723 (8289)	29774 (9965)	35789 (8028)
S[a]	1.07	1.03	1.06
$R_1(wR_2)$ ($F^2 > 2\sigma(F^2)$)	0.0673 (0.1902)	0.0307 (0.0821)	0.0571 (0.1493)
R_{int}	0.073	0.022	0.065
Min./max. diff map, Å^{-3}	–0.81, 0.78	–0.52, 0.52	–0.98, 1.35

[a]Conventional $R = \Sigma||Fo| - |Fc||/\Sigma|Fo|$; $Rw = [\Sigma w(Fo^2 - Fc^2)^2/\Sigma w(Fo^2)^2]^{1/2}$; $S = [\Sigma w(Fo^2 - Fc^2)^2/\text{no. data} - \text{no. params})]^{1/2}$ for all data.

Table S3: Crystallographic data for **1-Mg**, **1-Eu**, and **1-Sr**

	**1-Mg**	**1-Mg**	**1-Eu**	**1-Sr**
Formula	$C_{38}H_{58}MgN_2P_2Si_4$	$C_{38}H_{58}MgN_2P_2Si_4$	$C_{38}H_{58}EuN_2P_2Si_4$	$C_{38}H_{58}N_2P_2Si_4Sr$
Formula Weight	741.47	741.47	869.12	804.78
Crystal Size, mm	0.273×0.235×0.176	0.273×0.235×0.176	0.02×0.02×0.01	0.01×0.005×0.005
Crystal System	Monoclinic	Monoclinic	Monoclinic	Monoclinic
Space group	$C2/c$	$P2_1/c$	$P2_1/c$	$P2_1/c$
a, Å	25.4630(2)	12.8971(5)	13.5425(2)	13.5886(8)
b, Å	18.8370(2)	17.8425(8)	35.4008(7)	13.5886(8)
c, Å	20.6819(15)	19.5749(8)	9.2819(2)	9.2844(7)
α, °	90	90	90	90
β, °	117.260(2)	106.105(2)	98.0780(10)	98.146(4)
γ, °	90	90	90	90
V, Å^3	8818.3(11)	4327.7(3)	4405.73(14)	4420.7(5)
Z	8	4	4	4
Temperature, K	120	120	120	120
ρ_{calc}, g cm^{-3}	1.117	1.138	1.310	1.209
μ, mm^{-1}	2.273	2.315	12.115	3.614
F(*000*)	3184	1592	1796	1696
No. of reflections (unique)	44579 (8704)	40682 (8511)	48253 (8569)	29374 (4474)
S^a	1.14	1.06	1.14	1.44
$R_1(wR_2)$ ($F^2 > 2\sigma(F^2)$)	0.0748 (0.1529)	0.0669 (0.1982)	0.0642 (0.1310)	0.1428 (0.3699)
R_{int}	0.040	0.120	0.060	0.206
Min./max. diff map, Å^{-3}	–1.03, 0.67	–1.39, 1.18	–1.32, 1.16	–1.22, 1.85

^{a}Conventional $R = \Sigma||Fo| - |Fc||/\Sigma|Fo|$; $Rw = [\Sigma w(Fo^2 - Fc^2)^2/\Sigma w(Fo^2)^2]^{1/2}$; $S = [\Sigma w(Fo^2 - Fc^2)^2/\text{no. data} - \text{no. params})]^{1/2}$ for all data.

Table S4: Crystallographic data for **1-Sr·(THF)$_2$**, **1-Ba·(THF)$_2$**, **1-Sm·(THF)$_2$**, and **2-Sm.**

	**1-Sr·(THF)$_2$**	**1-Ba·(THF)$_2$**	**1-Sm·(THF)$_2$**	**2-Sm**
Formula	$C_{46}H_{74}N_2O_2P_2Si_4Sr$	$C_{46}H_{74}BaN_2O_2P_2Si_4$	$C_{46}H_{74}N_2O_2P_2Si_4Sm$	$C_{71}H_{103}N_3P_3Si_6Sm$
Formula Weight	949.02	998.71	1011.72	1410.43
Crystal Size, mm	0.608×0.581×0.485	0.212×0.158×0.092	0.360×0.299×0.174	0.437×0.344×0.344
Crystal System	Monoclinic	Monoclinic	Monoclinic	Triclinic
Space group	$P2_1/n$	$P2_1/n$	$P2_1/n$	P-1
a, Å	11.2183(2)	11.2946(3)	11.215(1)	18.6633(7)
b, Å	25.4398(4)	25.7725(8)	25.380(2)	19.7230(7)
c, Å	18.4800(3)	18.4396(6)	18.527(2)	21.0667(7)
α, °	90	90	90	95.504(2)
β, °	94.994(1)	95.036(2)	94.753(3)	102.574(2)
γ, °	90	90	90	90.180(2)
V, Å^3	5254.01(15)	5346.9(3)	5255.4(8)	7531.4(5)
Z	4	4	4	4
Temperature, K	120	120	120	120
ρ_{calc}, g cm^{-3}	1.200	1.241	1.279	1.244
μ, mm^{-1}	3.142	7.464	10.106	7.671
F(*000*)	2025	2088	2112	2964
No. of reflections (unique)	30963 (10179)	110944 (10598)	122146 (10402)	161374 (29607)
S^a	1.04	1.11	1.12	1.08
$R_1(wR_2)$ ($F^2 > 2\sigma(F^2)$)	0.0838 (0.2403)	0.0384 (0.0891)	0.0571 (0.1406)	0.0903 (0.2236)
R_{int}	0.110	0.065	0.073	0.070
Min./max. diff map, Å^{-3}	–1.97, 1.66	–0.89, 1.24	–0.93, 2.62	–0.83, 4.47

^{a}Conventional $R = \Sigma||Fo| - |Fc||/\Sigma|Fo|$; $Rw = [\Sigma w(Fo^2 - Fc^2)^2/\Sigma w(Fo^2)^2]^{1/2}$; $S = [\Sigma w(Fo^2 - Fc^2)^2$/no. data – no. params)]$^{1/2}$ for all data.

Table S5: Crystallographic data for **2-Y**, **2-La** and **2-Pr**.

	**2-Y**	**2-La**	**2-Pr**
Formula	$C_{71}H_{103}N_3P_3Si_6Y$	$C_{71.7}H_{103.8}LaN_3P_3Si_6$	$C_{71}H_{103}N_3P_3PrSi_6$
Formula Weight	1348.92	1408.13	1400.92
Crystal Size, mm	0.02×0.01×0.01	0.221×0.018×0.011	0.394×0.217×0.205
Crystal System	Triclinic	Hexagonal	Triclinic
Space group	*P*-1	$P6_322$	*P*-1
a, Å	18.64420(10)	17.5484(4)	18.7129(8)
b, Å	19.6403(2)	17.5484(4)	19.7729(7)
c, Å	21.0408(2)	15.0089(9)	21.0737(8)
α, °	95.6750(10)	90	95.295(2)
β, °	102.6880(10)	90	102.587(2)
γ, °	90.1210(10)	120	90.258(2)
V, $Å^3$	7477.43(12)	4002.7(3)	7575.1(5)
Z	4	2	4
Temperature, K	120	120	120
ρ_{calc}, g cm^{-3}	1.198	1.168	1.228
μ, mm^{-1}	2.928	5.836	6.739
F(*000*)	2872	1482	2952
No. of reflections (unique)	- (27257)*	86584 (2744)	124168 (32226)
S^a	1.10	1.12	1.20
$R_1(wR_2)$ ($F^2 > 2\sigma(F^2)$)	0.0967 (0.2400)	0.0835 (0.2126)	0.0659 (0.1489)
R_{int}	0.110	0.097	0.052
Min./max. diff map, $Å^{-3}$	–3.84, 3.49	–3.36, 2.62	–1.04, 1.63

[a]Conventional $R = \Sigma||Fo| - |Fc||/\Sigma|Fo|$; $Rw = [\Sigma w(Fo^2 - Fc^2)^2/\Sigma w(Fo^2)^2]^{1/2}$; $S = [\Sigma w(Fo^2 - Fc^2)^2$/no. data – no. params)]$^{1/2}$ for all data.

*twinning treatment

Table S6: Crystallographic data for **[La{N(SiMe3)2}3]**.

	**[La{N(SiMe3)2}3]**
Formula	$C_{24}H_{68}N_3LaSi_6$
Formula Weight	706.26
Crystal Size, mm	0.11×0.15×0.59
Crystal System	Trigonal
Space group	P-3_1c
a, Å	16.4920(2)
b, Å	16.4920(2)
c, Å	8.3189(2)
α, °	90
β, °	90
γ, °	120
V, Å^3	1959.49(8)
Z	2
Temperature, K	120
ρ_{calc}, g cm^{-3}	1.051
μ, mm^{-1}	10.293
F(*000*)	648
No. of reflections (unique)	9913 (1289)
S^a	1.17
$R_1(wR_2)$ ($F^2 > 2\sigma(F^2)$)	0.0421 (0.1053)
R_{int}	0.059
Min./max. diff map, Å^{-3}	–1.51, 0.93

^{a}Conventional $R = \Sigma||Fo| - |Fc||/\Sigma|Fo|$; $Rw = [\Sigma w(Fo^2 - Fc^2)^2/\Sigma w(Fo^2)^2]^{1/2}$; $S = [\Sigma w(Fo^2 - Fc^2)^2$/no. data – no. params)]$^{1/2}$ for all data.

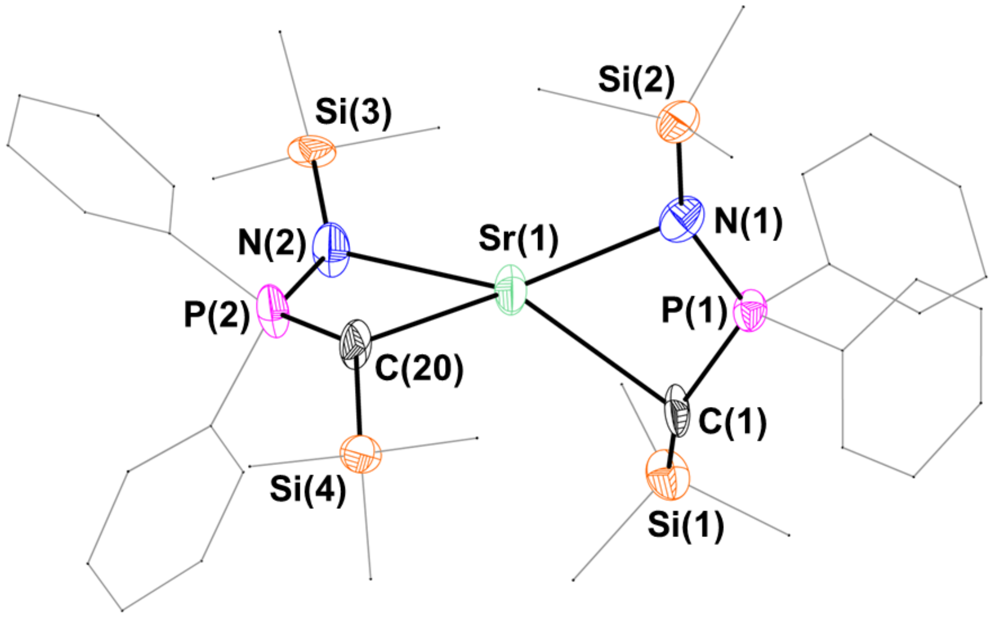

Figure S84: X-ray crystal structure of **1-Sr**. Ellipsoids set at 50% probability level; hydrogen atoms and disorder excluded, and phenyl and methyl of trimethylsilyl groups are shown as wireframe for clarity. C: black, N: blue, P: pink, Si: orange, Sr: aquamarine.

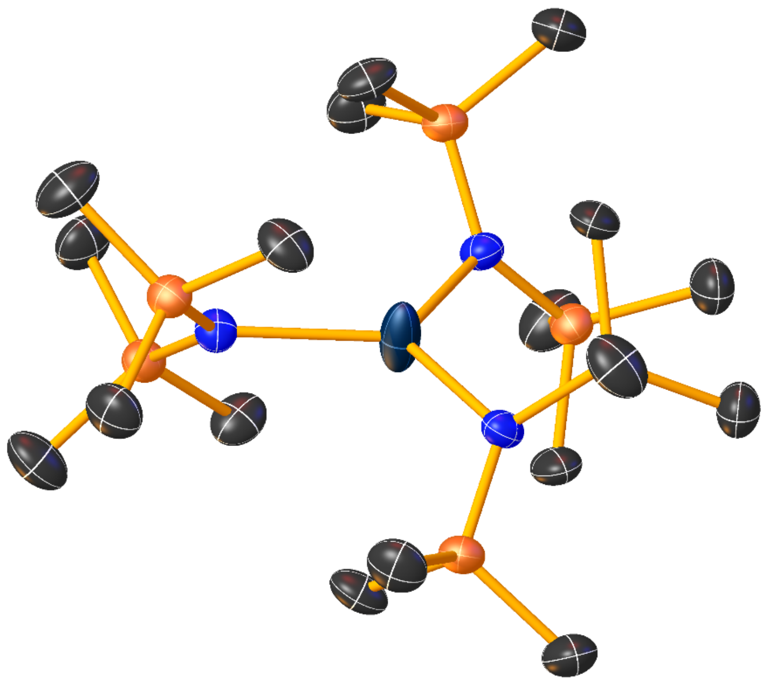

Figure S85: X-ray structure of **[La{N(SiMe₃)₂}₃]**. Ellipsoids are set at 50% probability level; hydrogen atoms have been omitted for clarity.

S5. Magnetic characterization

Magnetic measurements were made using a Quantum Design MPMS-XL7 superconducting quantum interference device (SQUID) magnetometer. Crystalline samples with mass 28.4 mg for **1-Eu** and 53.8 mg for **2-Sm** were crushed with a mortar and pestle under an inert atmosphere, and then loaded into a borosilicate glass NMR tube, which was then evacuated and flame-sealed to a length of ca. 5 cm. The measurements were corrected for the intrinsic diamagnetism of the sample using Pascals constants.[6] A final mass of 24 mg (relative weighing error of 15 %) was used to match the χT product at high temperature.

For 1**-Eu**, the magnetisation and susceptibility curves are consistent with what is to be expected of a spin-only S = 7/2 ground state ($\chi T = \left(g^2 S(S+1)\right)/8$, calculated as 7.85 $cm^3 K mol^{-1}$ at 300 K; $M_{sat} = gS$, calculated as 6.84 $N_A \mu_B$ at 3 K; with g = 2 and S = 7/2). For **2-Sm**, however, the weak magnetic moment of the sample prevented reproducible and consistent measurements – the magnetic properties of **2-Sm** are then not commented further.

S6. Theoretical calculations

The electronic structures of compounds **1-Eu** and **2-Sm** were studied by means of complete active space self-consistent field spin-orbit (CASSCF-SO) calculations, as implemented in OpenMolcas[7], where the active spaces are defined by considering seven and five 4f electrons in seven orbitals, for **1-Eu** and **2-Sm**, respectively. In a first step, we employed the molecular geometry from the single crystal XRD structure with no optimization, taking the largest disorder component only. **1-Eu** presented two almost equally populated conformers, where one of the axial ligands is displaced *ca* 0.5 Angstrom sideways, while the rest of the molecule remains the same. Thus, we have used an averaged structure (**1-Eu**) and both conformers (**1-Eu (A)** and **1-Eu (B)**) to study the effect of the geometry on the magnetic properties. **2-Sm** presented two unique molecules in the unit cell so we run the calculations in each of them ($\mathbf{2_1}$, $\mathbf{2_2}$). We use the second-order Douglas-Kroll-Hess relativistic decoupling[8] and approximate the two-electron integrals with a Cholesky (threshold 1.0d-8) resolution of the identity method.[9] Basis sets from ANO-RCC library[10,11] were employed with VTZP quality for Eu and Sm atoms, VDZP quality for the coordinating atoms and VDZP for all remaining atoms. The molecular orbitals (MOs) were optimized in a state-averaged calculations with equal weightings (Table S6) The resulting spin-only states were then mixed by spin orbit coupling using the RASSI module. The obtained spin-orbit wavefunctions (see Table S7-S9 and Table S11) were then used to calculate the magnetic susceptibility and magnetisation curves using SINGLE_ANISO.[12]

For **1-Eu**, magnetic anisotropy is introduced via spin-orbit interaction of the ground S=7/2 state with excited, low-spin states. The resulting anisotropy, that breaks the degeneracy of the ground $^8S_{7/2}$ term in four doublets, can be parametrised using a zero-field splitting Hamiltonian, which combined with a Zeeman Hamiltonian reads

$$\hat{H} = \hat{H}_{Zee} + \hat{H}_{ZFS} = \mu_B \hat{S}\bar{\bar{g}}\vec{B} + \left[D\left(\hat{S}_z^2 - \frac{\hat{S}^2}{3}\right) + E\left(\hat{S}_x^2 - \hat{S}_y^2\right)\right] \quad \text{Eq. (1)}$$

where D and E describe the axial and rhombic terms of the anisotropy, with $|D| \geq 3E \geq 0$. Importantly, the level of theory employed to describe the electronic states involved will change the obtained D and E values, which in turn will result in hugely different EPR spectra, and a careful evaluation is required. Starting with **1-Eu** and Approach I (Table S6), where we consider all spin states available and mix the 48 sextets with the ground octet, we observe four Kramers doublets (KDs) well-isolated from the rest of the spin-orbit coupled states at 52000 K

(Table S7) – parametrising these four KDs in terms of the ZFS Hamiltonian, one obtains D and E parameters (Table S10) that do not reproduce the EPR data (Figure S78 and S79). Inspection of the wavefunction composition of the four low-lying KDs reveals that they are made up of linear combinations of the ground $^8S_{7/2}$ free ion term and the states coming from the first excited ^6P term. This then allows us to simplify the calculation and consider just one S=8 and 3 S=6 states, denoted as Approach II. This results in an overall splitting of the low-lying KDs twice as large, resulting in a considerable larger D value and a non-vanishing E term (Table S10). Using these parameters the EPR data is still not reproduced (Figure S78 and S79), but the agreement with experiment is significantly better, highlighting the importance of employing a set of orbitals that have been optimised for the right number of states. We then assessed the effect of including dynamic electronic correlation by means of extended multi-state (XMS)[13,14] calculations within the CASPT2 formalism[15] (Approach III). Here, we had to employ an imaginary shift of 0.2 (Table S11, Figure S80) for the results to converge. Using the wavefunctions obtained with this approach, the ZFS values increase further but the agreement with experiment is still poor. All three approaches provide very similar profiles for the magnetisation curves and susceptibility traces, as expected since at high temperatures and high fields the differences in the calculated ZFS parameters are masked. For **2-Sm** we obtain the same results in the two unique molecules found in the unit cell, so we only present data for one of them.

Table S6. Summary of states used for each spin multiplicity for compounds **1-Eu** and **2-Sm**. For each cell under Spin Multiplicity, the first value refers to the number of states used to optimise the molecular orbitals in a state averaged calculation and the second value refers to the number of states used in the state interaction calculation to account for the spin-orbit coupling.

Compound	Method	Spin multiplicity				Approach
		8	6	4	2	
**1-Eu**	CAS(7,7)SCF-RASSI-SO	1 / 1	48 / 48	392 / 0	784 / 0	I
	CAS(7,7)SCF-RASSI-SO	1 / 1	3 / 3	-	-	II
	XMSCAS(7,7)PT2-RASSI-SO	1 / 1	3 / 3	-	-	III
**1-Eu (A)**	CAS(7,7)SCF-RASSI-SO	1 / 1	3 / 3	-	-	II
**1-Eu (B)**	CAS(7,7)SCF-RASSI-SO	1 / 1	3 / 3	-	-	II
**2-Sm**	CAS(5,7)SCF-RASSI-SO	-	21 / 21	224 / 128	490 / 130	IV

Table S7. Spin-free and spin-orbit coupled energies (in cm^{-1}) calculated at the crystal structure of **1-Eu**. For spin free states, only the spin octet and sextet states are shown.

Approach I							
Spin-free				Spin-orbit			
state	Energy (cm^{-1})	state	Energy (cm^{-1})	state	Energy (cm^{-1})	state	Energy (cm^{-1})
1	0.00	25	58311.16	1	0.00	25	37091.34
2	36446.47	26	58352.05	2	0.00	26	37091.34
3	36536.78	27	58649.09	3	1.07	27	39448.58
4	36554.47	28	58689.93	4	1.07	28	39448.58
5	38917.35	29	58814.41	5	1.78	[…]	[…]
6	38917.36	30	58879.54	6	1.78	296	67962.04
7	38969.33	31	58936.92	7	2.18		
8	38969.66	32	59683.17	8	2.18		
9	39033.07	33	59865.28	9	36185.84		
10	39035.18	34	59946.37	10	36185.84		
11	39093.99	35	60339.96	11	36298.76		
12	39097.68	36	60380.11	12	36298.76		
13	39139.84	37	60528.38	13	36444.39		
14	39155.60	38	60544.46	14	36444.39		
15	39175.65	39	65981.26	15	36648.15		
16	39198.16	40	65981.89	16	36648.15		
17	39214.42	41	66187.27	17	36657.57		
18	43623.26	42	66206.53	18	36657.57		
19	43726.45	43	66220.01	19	36823.10		
20	43760.52	44	66229.62	20	36823.10		
21	43950.47	45	66263.87	21	36849.46		
22	43979.80	46	66277.17	22	36849.46		
23	58136.94	47	66309.22	23	37048.45		
24	58198.79	48	66315.53	24	37048.45		
		49	66327.25				

Approach II			
Spin-free		Spin-orbit	
state	Energy (cm^{-1})	state	Energy (cm^{-1})
1	0.00	1	0.00
2	36411.91	2	0.00
3	36482.12	3	2.10
4	36497.21	4	2.10
		5	3.44
		6	3.44
		7	4.24
		8	4.24
		9	36883.88
		[…]	[…]
		26	37965.33

Approach III			
Spin-free		Spin-orbit	
state	Energy (cm^{-1})	state	Energy (cm^{-1})
1	0.00	1	0.00
2	30603.02	2	0.00
3	30775.62	3	2.63
4	30811.85	4	2.63
		5	4.28
		6	4.28
		7	5.31
		8	5.31
		9	31231.50
		[…]	[…]
		26	32440.87

Table S8. Spin-free and spin-orbit coupled energies (in cm^{-1}) calculated at the crystal structure of **1-Eu (A)**.

Approach II			
Spin-free		Spin-orbit	
state	Energy (cm^{-1})	state	Energy (cm^{-1})
1	0.00	1	0.00
2	36427.16	2	0.00
3	36492.72	3	1.98
4	36511.22	4	1.98
		5	3.20
		6	3.20
		7	4.51
		8	4.51
		9	36915.76
		[…]	[…]
		26	37965.58

Table S9. Spin-free and spin-orbit coupled energies (in cm^{-1}) calculated at the crystal structure of **1-Eu (B)**.

Approach II			
Spin-free		Spin-orbit	
state	Energy (cm^{-1})	state	Energy (cm^{-1})
1	0.00	1	0.00
2	36463.29	2	0.00
3	36554.64	3	2.93
4	36563.34	4	2.93
		5	4.85
		6	4.85
		7	5.84
		8	5.84
		9	36983.77
		[…]	[…]
		26	37987.56

Table S10. Comparison of the model spin Hamiltonian parameters (eq. 1) for the investigated conformers of **1-Eu** obtained at different levels of theory (see Table S6) and from simulating the Q-band EPR data at 298 K, respectively. The "Label" entry refers to the labels used in Figure S78 and S79.

conformer	Approach	g	D (MHz)	E (MHz)	Label
**1-Eu**	I	1.997	-5387.2	0.0	2
	II	1.997	-10417.9	-791.5	3
	III	1.996	-12948.0	-1106.2	4
**1-Eu (A)**	II	1.998	-10195.9	2167.5	5
**1-Eu (B)**	II	1.998	-14536.9	461.7	6
EPR		1.989	-7620	300	1

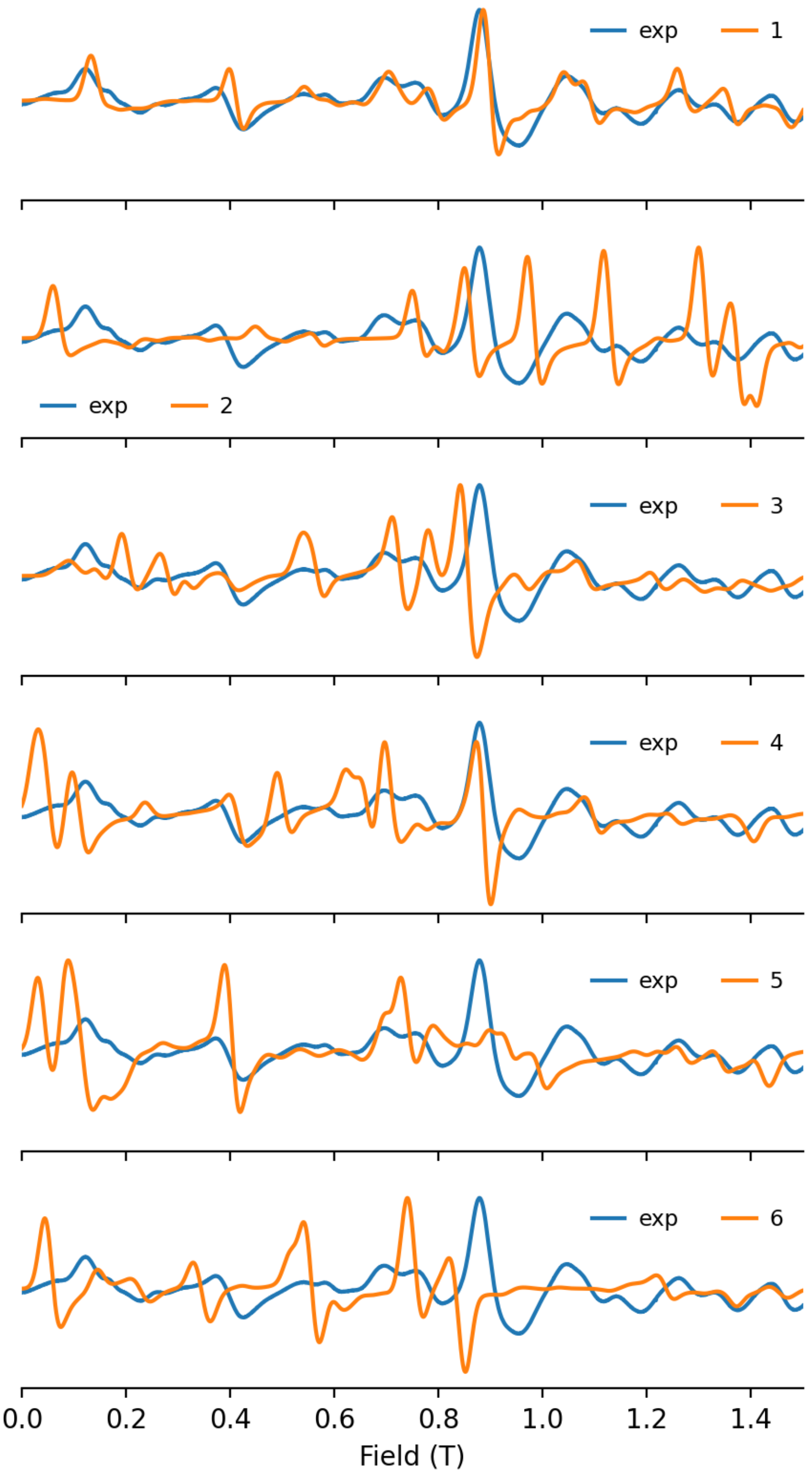

Figure S86: EPR spectra of **1-Eu.** Comparing normalised Q-band spectra using the ZFS parameters reported in Table S10, at 298 K.

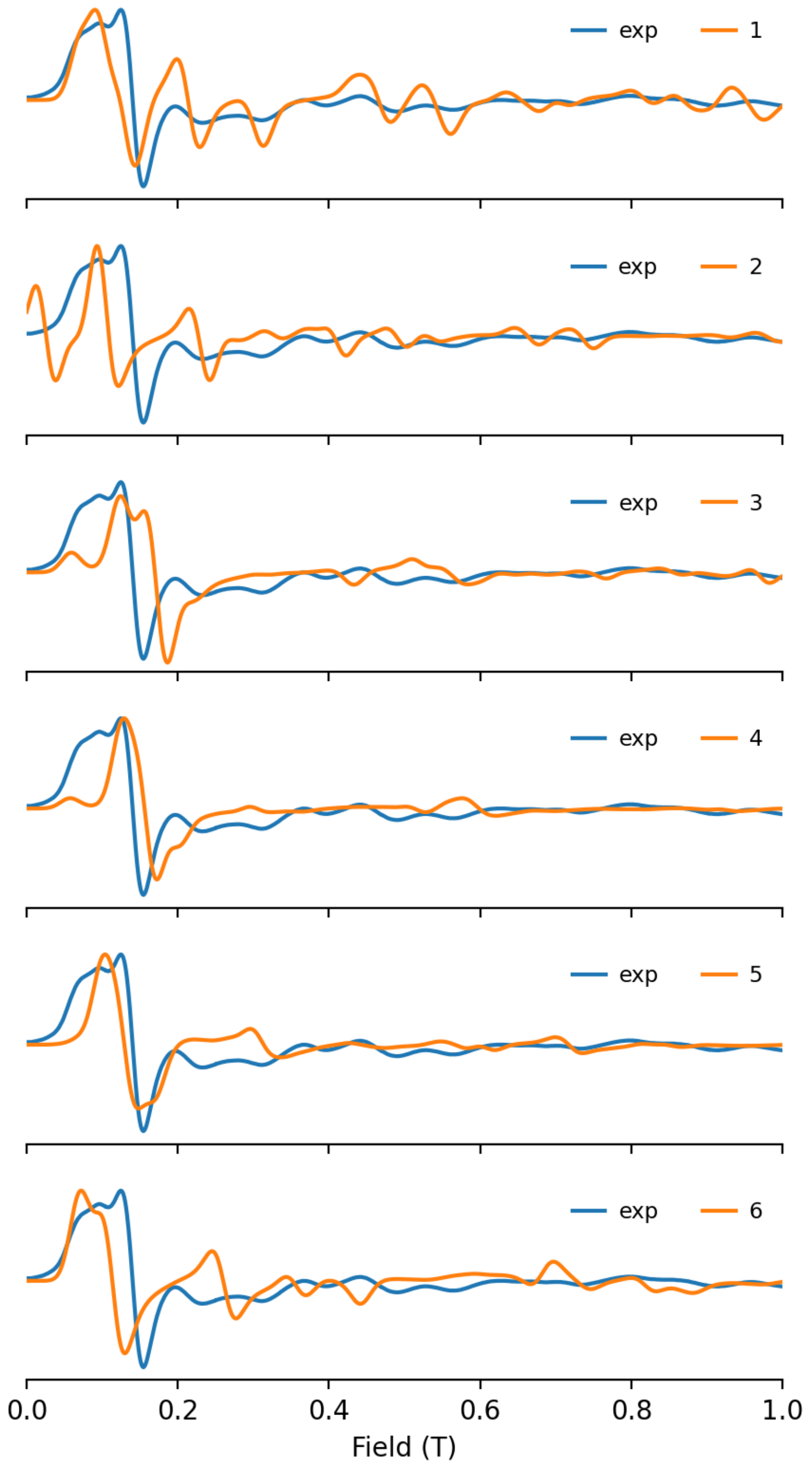

Figure S87: EPR spectra of **1-Eu.** Comparing normalised X-band spectra using the ZFS parameters reported in Table S10, at 298 K.

Table S11: Absolute energies of the ground and first three spin-free octet and sextet states, respectively, calculated using Approach III, as a function of the imaginary shift for **1-Eu**. These energies have been calculated using the orbitals optimised for the 48 sextets, but doing a CASCI of the three lowest states.

	Imaginary shift (a.u.)				
	0.1	0.2	0.3	0.4	0.6
$^{8}S_{7/2}$	-14262.4230	-14262.4182	-14262.4147	-14262.4090	-14262.3865
^{6}P	-14262.2892	-14262.2844	-14262.2802	-14262.2741	-14262.2507
	-14262.2884	-14262.2839	-14262.2798	-14262.2737	-14262.2503
	-14262.2882	-14262.2838	-14262.2798	-14262.2737	-14262.2503

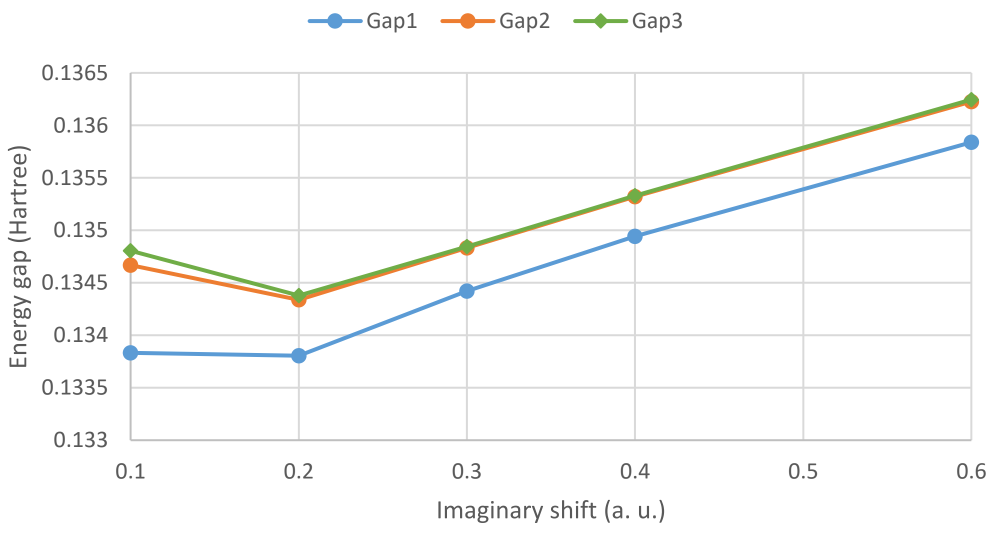

Figure S88: Energy gaps of the three sextet states with respect to the ground octet, as a function of imaginary shift for **1-Eu**.

Table S12: Spin-free and spin-orbit coupled energies (in cm^{-1}), calculated at the CAS(5,7)SCF-RASSI-SO level, of the low-lying states calculated at the crystal structure of **2-Sm**. For spin-only states, white, light grey and dark grey indicate sextet, quartet and doublet spin multiplicities, respectively. For spin-orbit coupled states, each row is a Kramers doublet, where the g-values of the first 7 doublets are also shown.

Spin-only	Spin-orbit			
E (cm^{-1})	E (cm^{-1})	g_x	g_y	g_z
0.00	0.00	0.693	0.199	0.134
17.19	170.92	1.042	0.607	0.230
297.05	270.23	1.455	0.423	0.112
341.46	1123.47	2.532	1.838	0.273
378.80	1194.77	2.041	1.860	0.517
422.73	1292.09	2.295	1.787	0.485
469.28	1366.70	4.077	1.360	1.012
490.23	2405.13			
649.53	[…] x 889			
683.54	50956.54			
734.68				
6968.63				
7023.57				
7026.67				
7095.61				
7125.48				
7144.76				
7210.41				
22279.53				
x 94				
31251.55				
31307.74				
31427.67				
31690.82				

33463.56
[…] x 9
33700.40
[…] x 607
159846.60

S7. Supplementary References

(1) Sheldrick, G. M. *Program for Area Detector Absorption Correction, Institute for Inorganic Chemistry*; University of Göttingen: Göttingen, Germany, Germany, 1996.

(2) *CrysAlisPro Software System*, Version 1.171.42a, Rigaku Corporation, Wroclaw, Poland, 2024.

(3) Sheldrick, G. M. A Short History of SHELX. *Acta Crystallogr. Sect. A* **2008**, *64* (1), 112–122.

(4) Sheldrick, G. M. Crystal Structure Refinement with SHELXL. *Acta Crystallogr. Sect. C* **2015**, *71*, 3–8.

(5) Dolomanov, O. V.; Bourhis, L. J.; Gildea, R. J.; Howard, J. A. K.; Puschmann, H. OLEX2: A Complete Structure Solution, Refinement and Analysis Program. *J. Appl. Crystallogr.* **2009**, *42* (2), 339–341.

(6) Bain, G. A.; Berry, J. F. Diamagnetic Corrections and Pascal's Constants. *J. Chem. Educ.* **2008**, *85* (4), 532.

(7) Fdez. Galván, I.; Vacher, M.; Alavi, A.; Angeli, C.; Aquilante, F.; Autschbach, J.; Bao, J. J.; Bokarev, S. I.; Bogdanov, N. A.; Carlson, R. K.; Chibotaru, L. F.; Creutzberg, J.; Dattani, N.; Delcey, M. G.; Dong, S. S.; Dreuw, A.; Freitag, L.; Frutos, L. M.; Gagliardi, L.; Gendron, F.; Giussani, A.; González, L.; Grell, G.; Guo, M.; Hoyer, C. E.; Johansson, M.; Keller, S.; Knecht, S.; Kovačević, G.; Källman, E.; Li Manni, G.; Lundberg, M.; Ma, Y.; Mai, S.; Malhado, J. P.; Malmqvist, P. Å.; Marquetand, P.; Mewes, S. A.; Norell, J.; Olivucci, M.; Oppel, M.; Phung, Q. M.; Pierloot, K.; Plasser, F.; Reiher, M.; Sand, A. M.; Schapiro, I.; Sharma, P.; Stein, C. J.; Sørensen, L. K.; Truhlar, D. G.; Ugandi, M.; Ungur, L.; Valentini, A.; Vancoillie, S.; Veryazov, V.; Weser, O.; Wesołowski, T. A.; Widmark, P.-O.; Wouters, S.; Zech, A.; Zobel, J. P.; Lindh, R. OpenMolcas: From Source Code to Insight. *J. Chem. Theory Comput.* **2019**, *15* (11), 5925–5964.

(8) Reiher, M. Douglas–Kroll–Hess Theory: A Relativistic Electrons-Only Theory for Chemistry. *Theor. Chem. Acc.* **2006**, *116* (1), 241–252.

(9) Aquilante, F.; Gagliardi, L.; Pedersen, T. B.; Lindh, R. Atomic Cholesky Decompositions: A Route to Unbiased Auxiliary Basis Sets for Density Fitting Approximation with Tunable Accuracy and Efficiency. *J. Chem. Phys.* **2009**, *130* (15), 154107.

(10) Roos, B. O.; Lindh, R.; Malmqvist, P.-Å.; Veryazov, V.; Widmark, P.-O. Main Group Atoms and Dimers Studied with a New Relativistic ANO Basis Set. *J. Phys. Chem. A* **2004**, *108* (15), 2851–2858.

(11) Roos, B. O.; Lindh, R.; Malmqvist, P.-Å.; Veryazov, V.; Widmark, P.-O. New Relativistic ANO Basis Sets for Transition Metal Atoms. *J. Phys. Chem. A* **2005**, *109* (29), 6575–6579.

(12) Ungur, L.; Chibotaru, L. F. Ab Initio Crystal Field for Lanthanides. *Chem. – Eur. J.* **2017**, *23* (15), 3708–3718.

(13) Granovsky, A. A. Extended Multi-Configuration Quasi-Degenerate Perturbation Theory: The New Approach to Multi-State Multi-Reference Perturbation Theory. *J. Chem. Phys.* **2011**, *134* (21), 214113.

(14) Shiozaki, T.; Győrffy, W.; Celani, P.; Werner, H.-J. Communication: Extended Multi-State Complete Active Space Second-Order Perturbation Theory: Energy and Nuclear Gradients. *J. Chem. Phys.* **2011**, *135* (8), 081106.

(15) Finley, J.; Malmqvist, P.-Å.; Roos, B. O.; Serrano-Andrés, L. The Multi-State CASPT2 Method. *Chem. Phys. Lett.* **1998**, *288* (2), 299–306. https://doi.org/10.1016/S0009-2614(98)00252-8.
